# Supplementary material for: Defect-enriched iron fluoride-oxide nanoporous thin films bifunctional catalyst for water splitting
Source: Nat Commun. 2018 May 4;9:1809. doi: 10.1038/s41467-018-04248-y (PMC5935708; doi:10.1038/s41467-018-04248-y)
Supplement: Supplementary file 1 — Supplementary Information [file 41467_2018_4248_MOESM1_ESM.pdf]

Defect-Enriched Iron Fluoride-Oxide Nanoporous Thin Films Bifunctional Catalyst for Water Splitting

Xiujun et al.

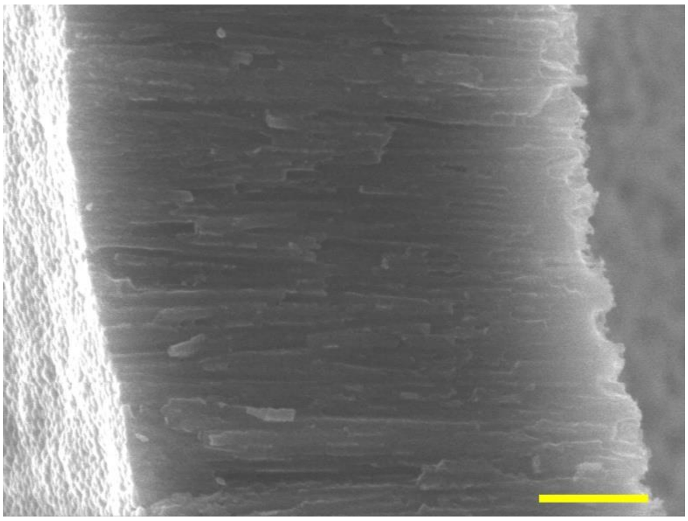

Supplementary Figure 1 | Cross section SEM image of the Fe-oxide nanoporous layers. After anodization, a self-organized and nanoporous structure with nanochannels was observed in SEM image. Scale bar, 1 μm.

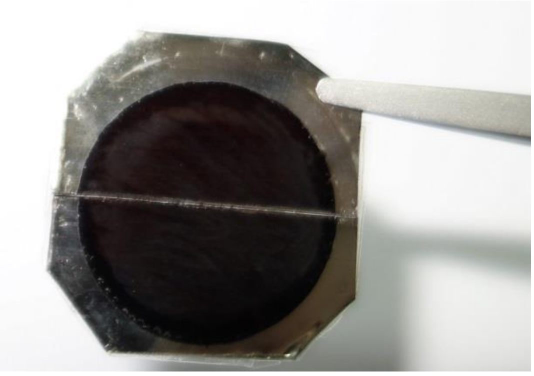

Supplementary Figure 2 | Optical image of IFONFs-45. Optical image of IFONFs-45 on a 2 inch wafer scale with fluorine vapor assisted fluorination at 350 ℃, indicating the process is scalable and the product holds a uniform surface. It should be noted that the mark line at the middle of the wafer is caused by cutting.

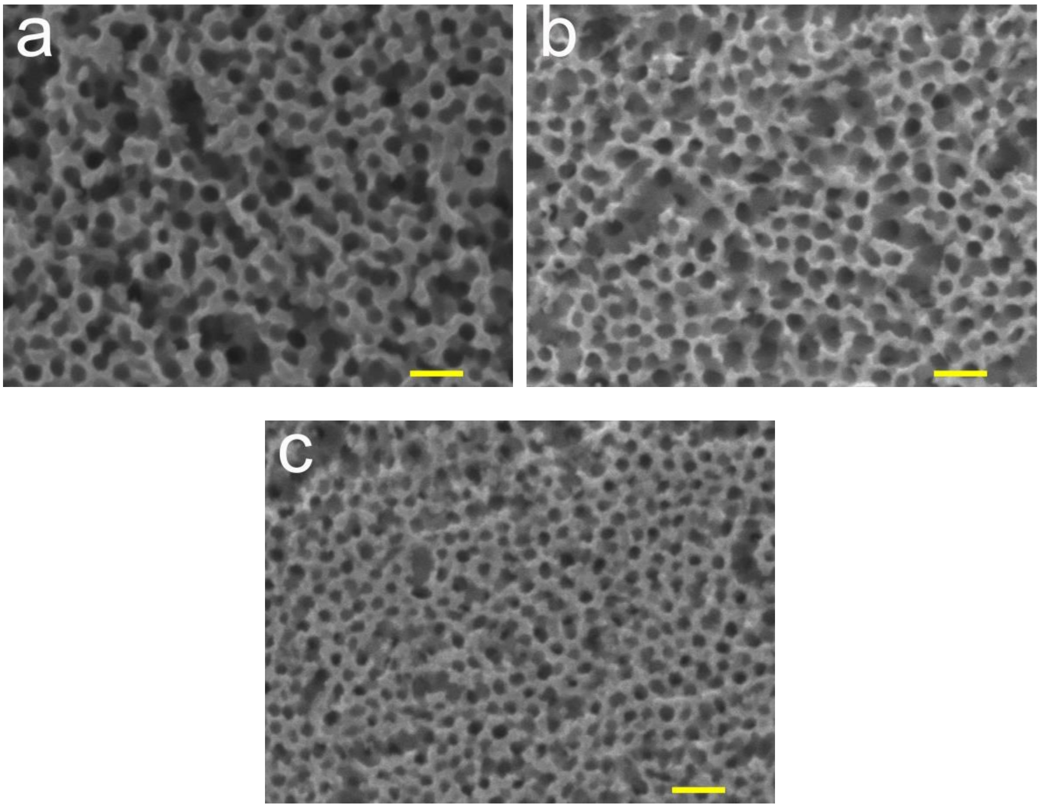

Supplementary Figure 3 | SEM characterization of IFONFs. SEM images of IFONFs fluorinated at 350 ^{o}C with various time, for **a** 15 min, **b** 30 min, **c** 90 min. Scale bar, **a-c** 100 nm.

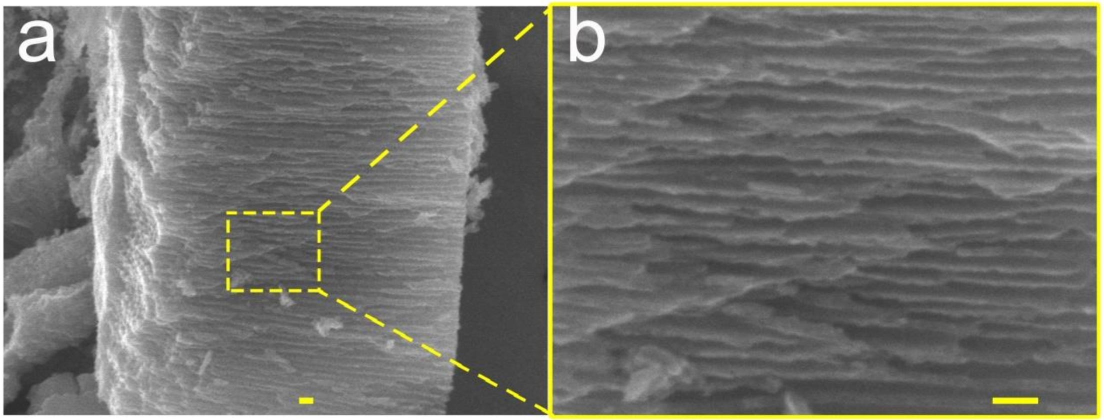

Supplementary Figure 4 | Cross-section SEM images of IFONFs-30. a,b Typical stripe-like nanoscale grains are clearly seen in SEM images. Scale bar, **a,b** 100 nm.

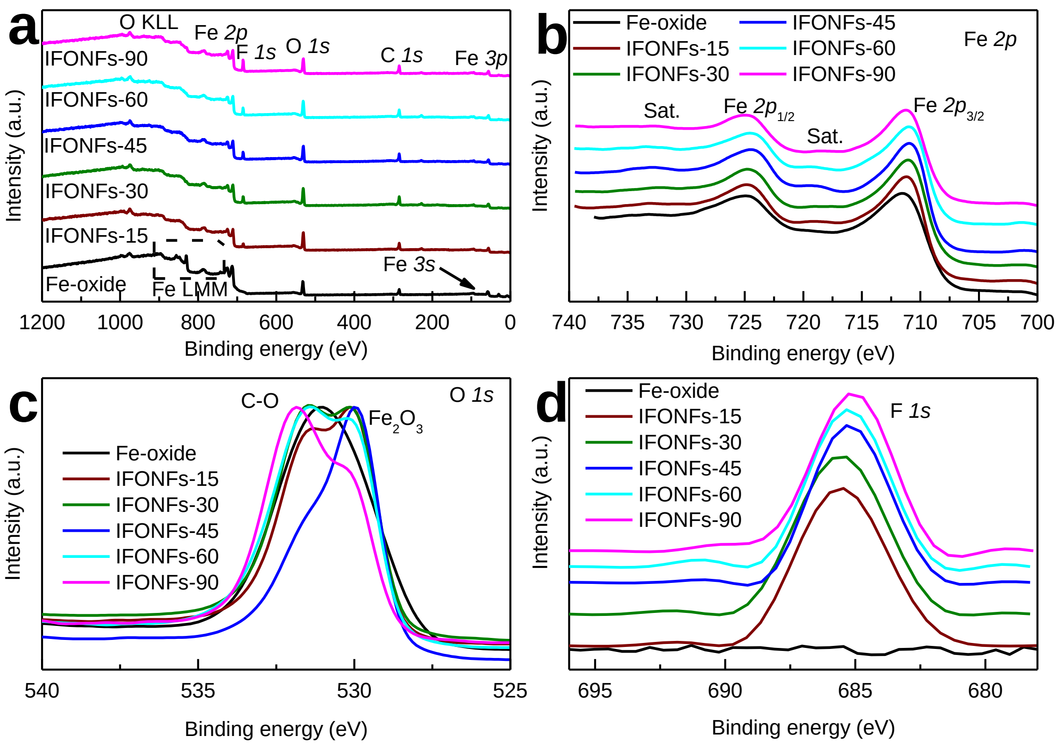

Supplementary Figure 5 | XPS results of Fe-oxide PTF and IFONFs. a XPS survey, **b** Fe *2p*, **c** O *1s*, and **d** F *1s* spectra of Fe-oxide PTF and IFONFs prepared at 350 °C with various time.

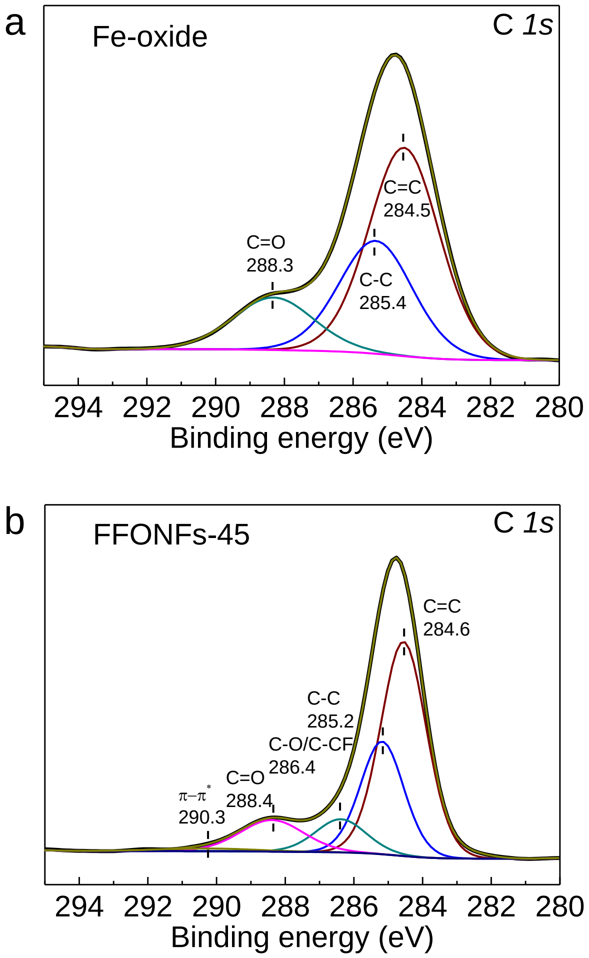

Supplementary Figure 6 | XPS spectra of Fe-oxide PTF and IFONFs-45. High-resolution XPS spectra in the C *1s* region of **a** Fe-oxide PTF and **b** IFONFs-45.

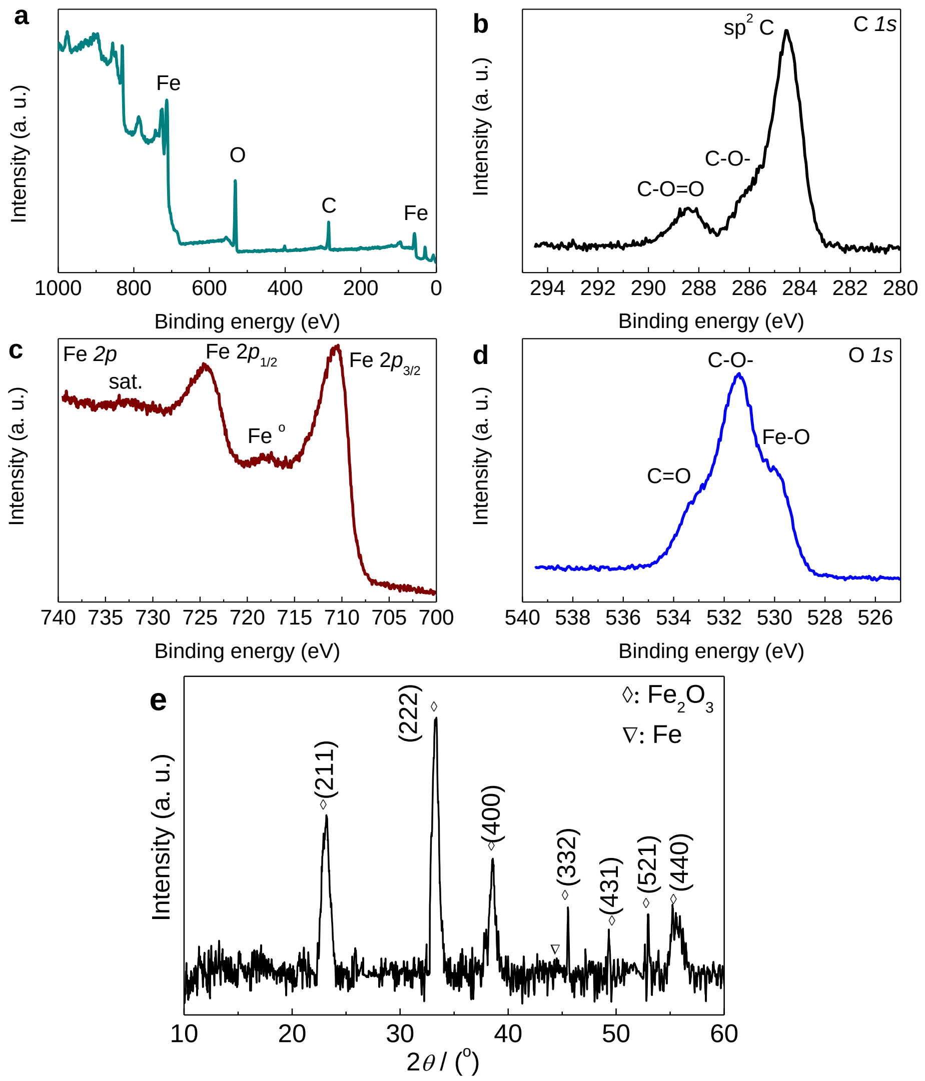

Supplementary Figure 7 | XPS spectra and crystal phase of Fe_2O_3 thin film. a XPS survey spectra **b** C *1s*, **c** Fe *2p*, and **d** O *1s* together with **e** XRD spectrum of Fe_2O_3 thin film.

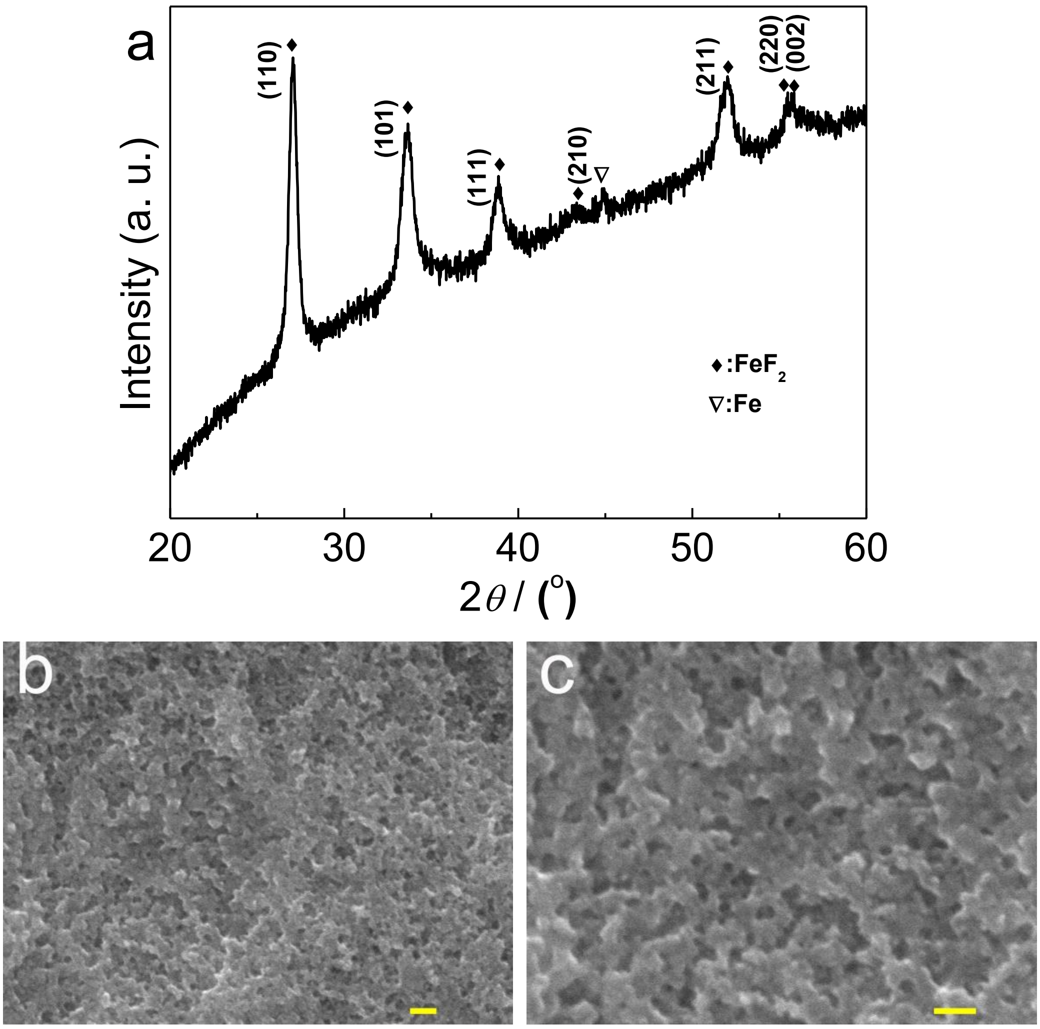

Supplementary Figure 8 | XRD result and SEM image of FeF_2 PTF. a XRD spectrum and **b,c** SEM image of FeF_2 PTF prepared through fluorination reaction with Fe_2O_3 PTF at 350 ^oC for 45 min. Scale bar, **b,c** 100 nm.

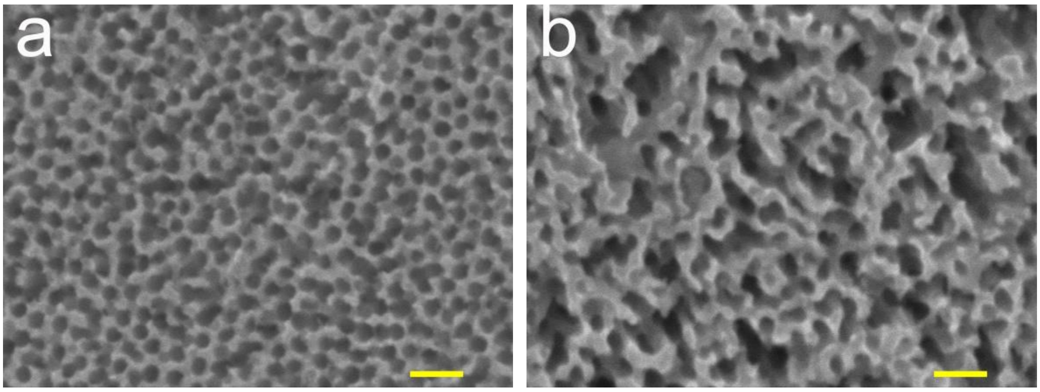

Supplementary Figure 9 | SEM images of different IFONFs catalysts. SEM images of IFONFs fluorinated at different temperature with 45 min, for **a** 300 ^{o}C and **b** 400 ^{o}C. Scale bar, **a,b** 100 nm. As shown in **b**, any attempt to further increase the fluorination temperature over 350 ^{o}C causes extreme sintering, which destroys the ordered porous structure of IFONFs, resulting in the reduction of the exposed edge sites[1].

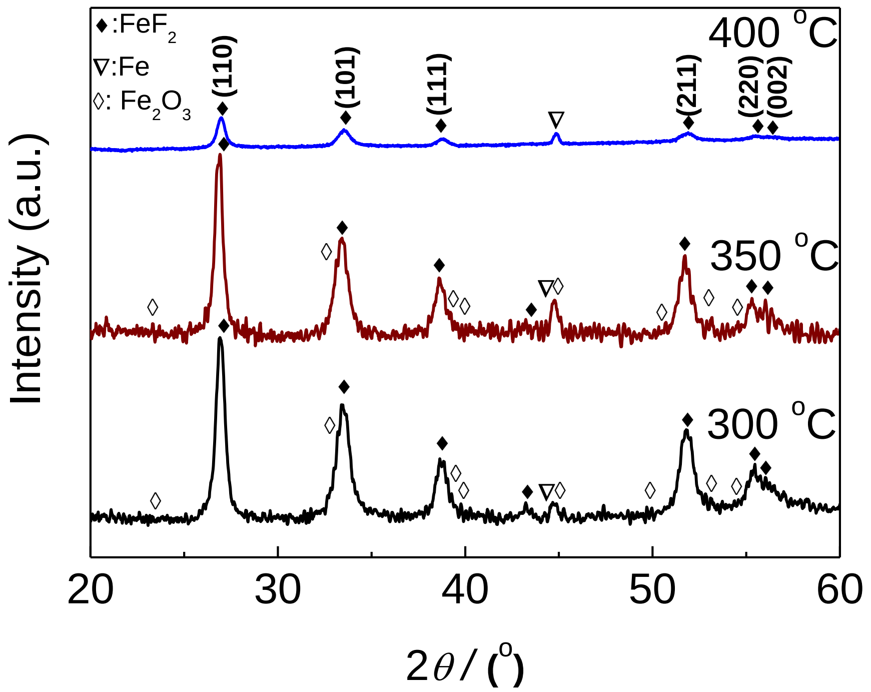

Supplementary Figure 10 | XRD patterns of different IFONFs catalysts. XRD patterns of IFONFs fluorinated at different temperature with 45 min, from 300 ^{o}C to 400 ^{o}C.

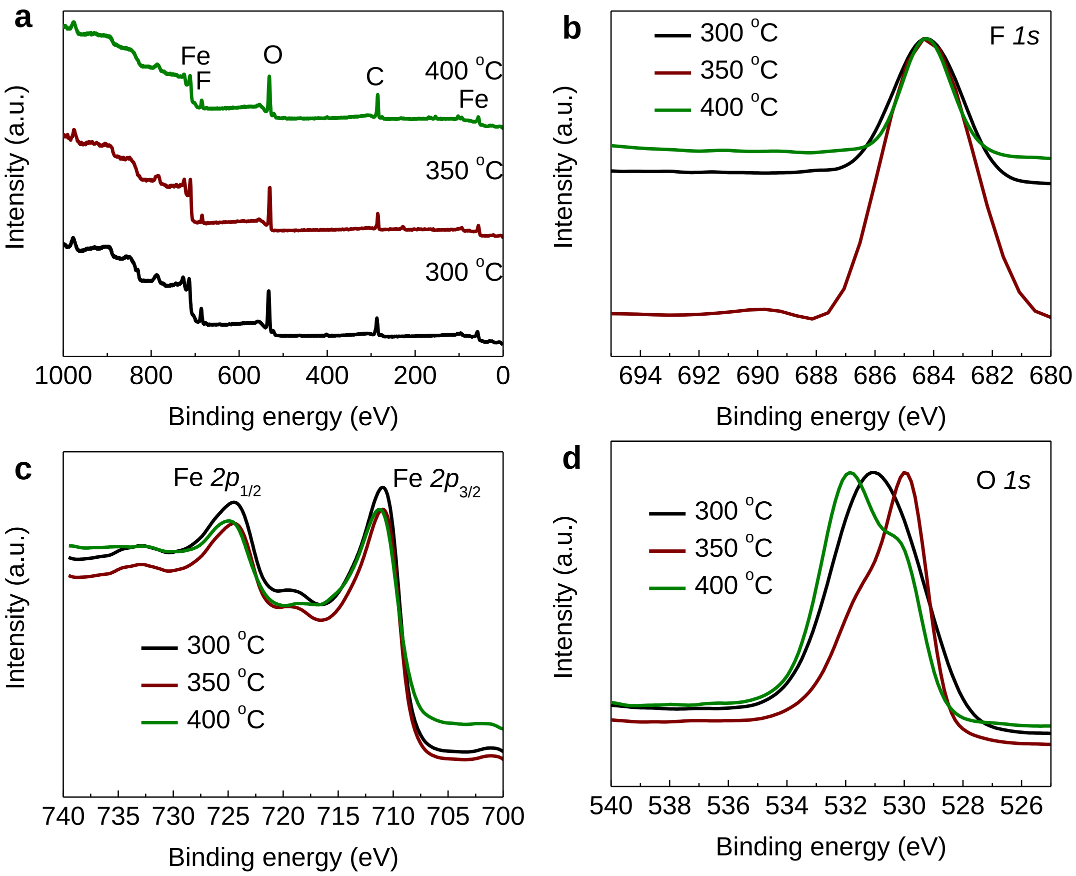

Supplementary Figure 11 | XPS spectra of different IFONFs catalysts. XPS spectrum of IFONFs fluorinated at different temperature with 45 min, from 300 °C to 400 °C, **a** XPS survey spectra **b** F *1s*, **c** Fe *2p*, and **d** O *1s*.

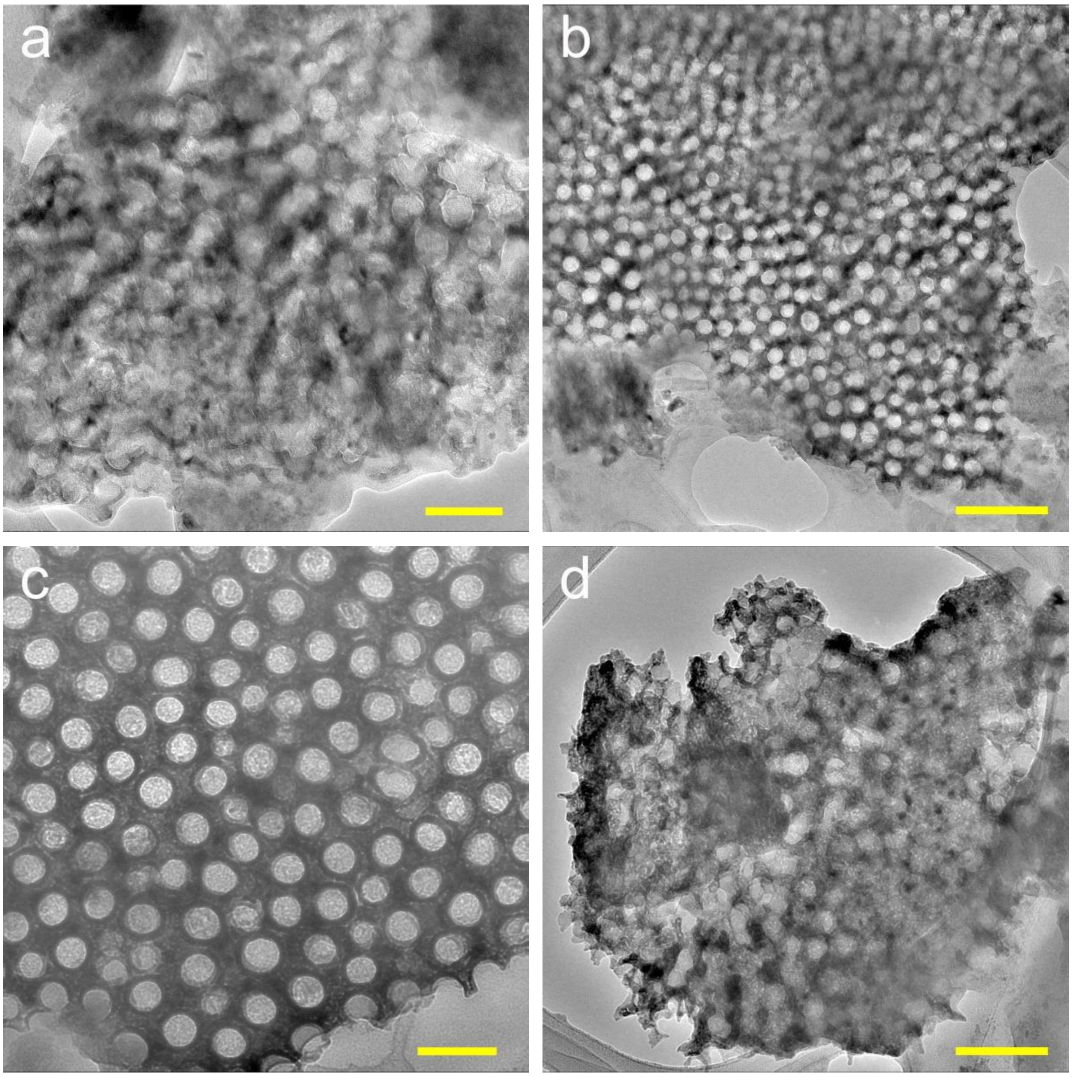

Supplementary Figure 12 | Morphological properties of of IFONFs. TEM image of IFONFs fluorinated at 350 °C with various time, for **a** 15, **b** 30, **c** 60, and **d** 90 min. Scale bar, **a,c** 100 nm; **b,d** 200 nm.

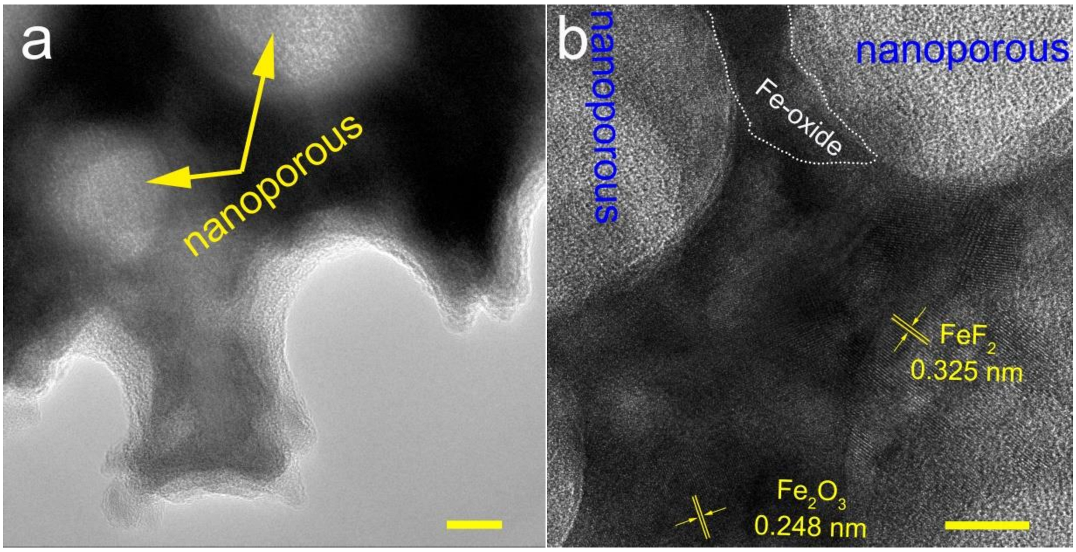

Supplementary Figure 13 | Typical HRTEM images of IFONFs. HRTEM image of the IFONFs-45 demonstrates the nanoporous structures for the iron fluoride-oxide thin film. Scale bar, **a,b** 10 nm. The amorphous nanodomain in **b** (marked by white dashed line) is the residuum of as-formed Fe-oxide nanoporous film

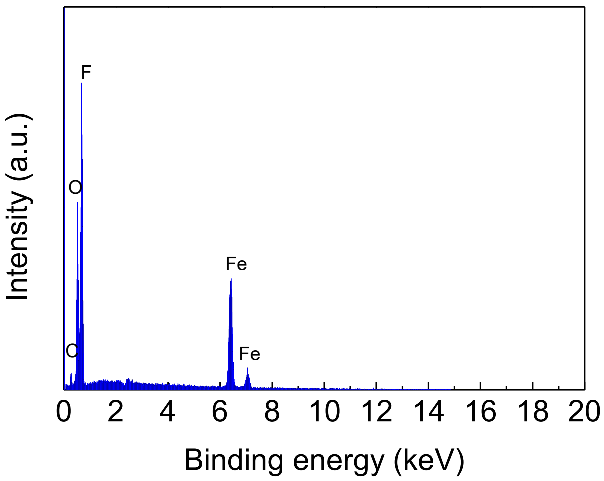

Supplementary Figure 14 | EDS of IFONFs-45. The analysis indicates the IFONFs-45 hybrid contains around 18.37 at% of Fe, 35.68 at% of C, 36.24 at% of O, and 9.71 at% of F. The atomic ratio of F to O is calculated to be around 0.3.

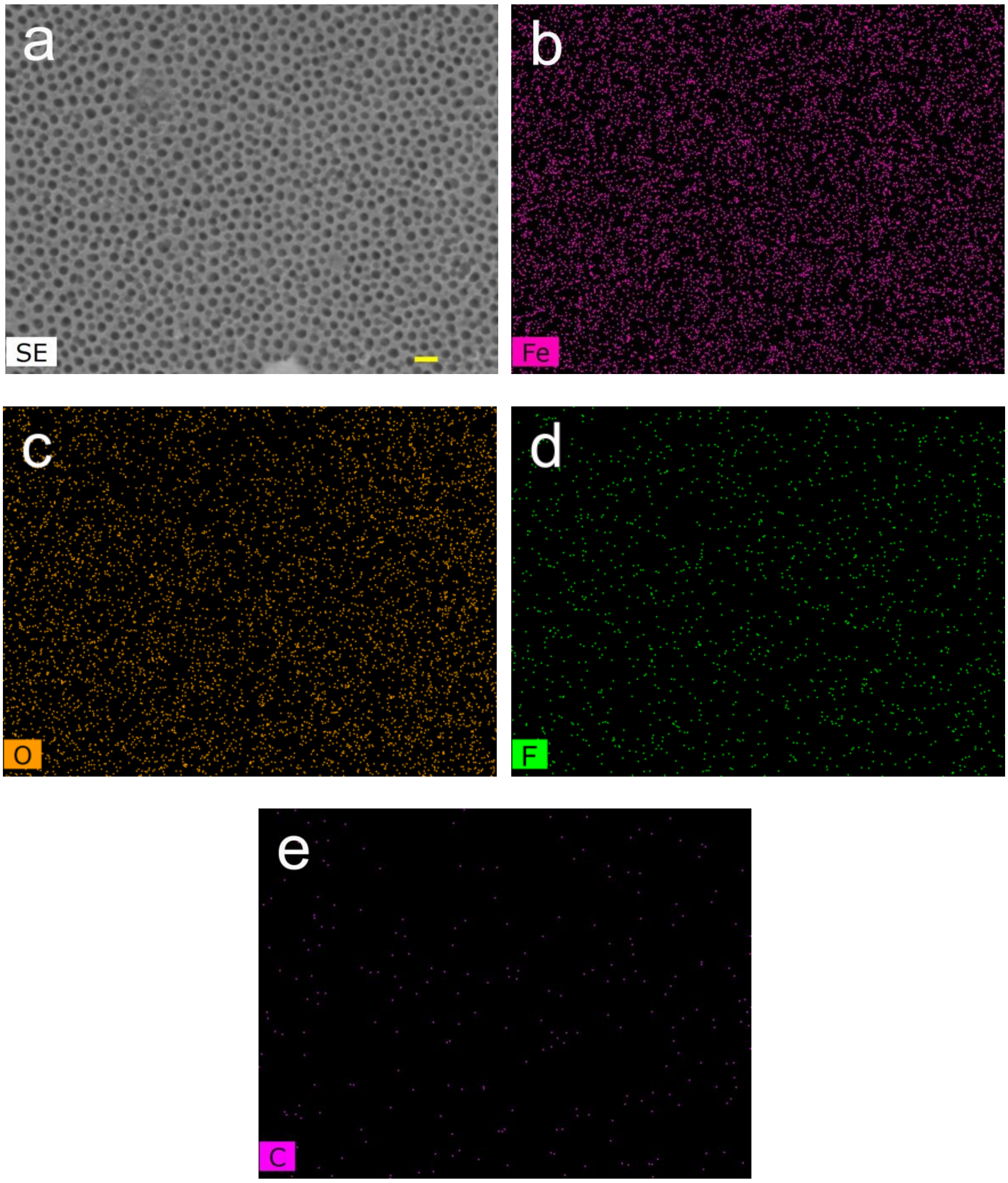

Supplementary Figure 15 | SEM and EDS mapping of IFONFs. **a** SEM image of iron fluoride-oxide nanoporous film, and EDS mapping of the thin film, **b** Fe, **c** O, **d** F, and **e** C. Scale bar, 100 nm.

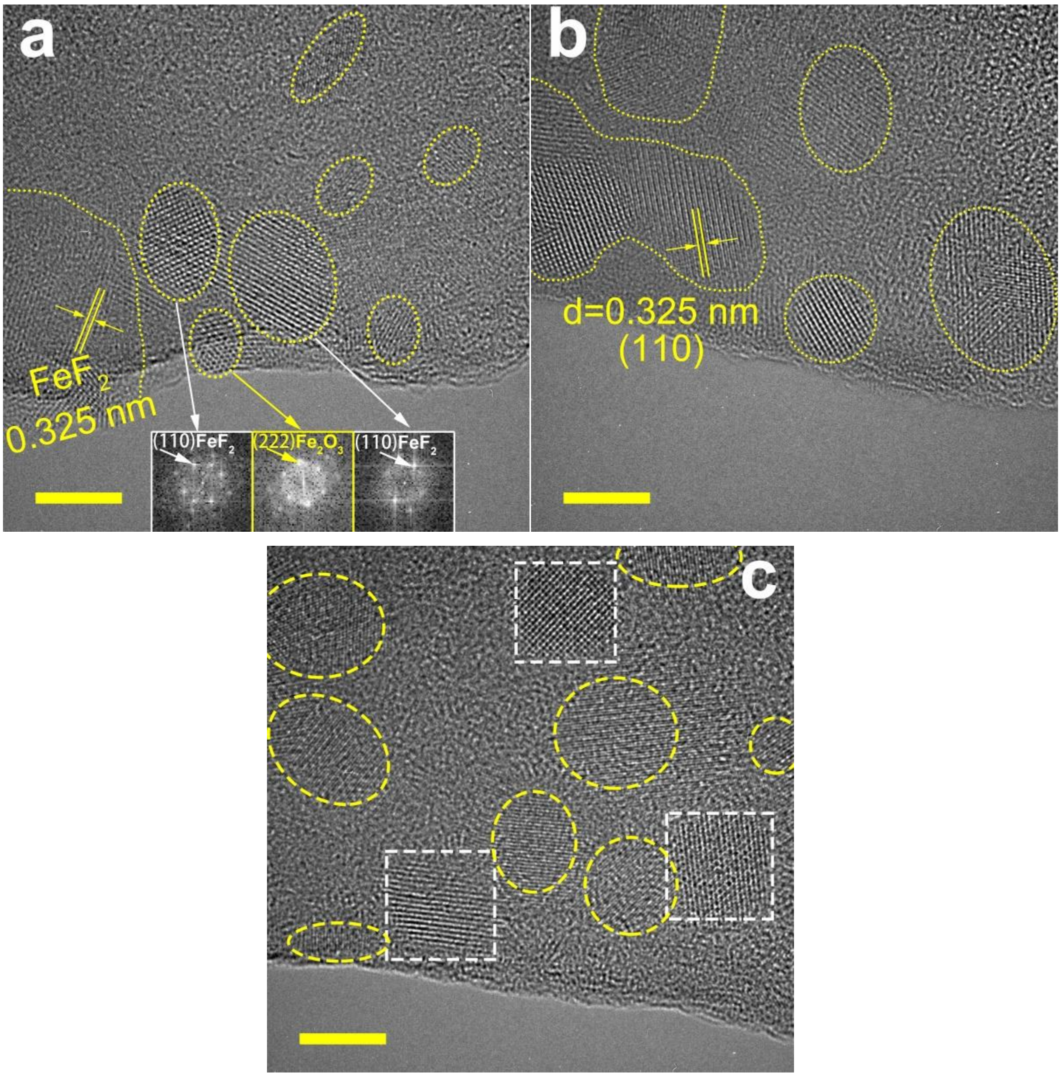

Supplementary Figure 16 | TEM images of IFONFs-15. a HRTEM images and corresponding FFT images of surface regions of iron fluoride-oxide nanoporous film. The corresponding FFT patterns recorded from the atomic scale image delineates the presence of (110) plane with inter-planar spacings of 0.325 (from single-crystalline grafted FeF_2 (PDF#81-2271) and 0.27 nm (from Fe_2O_3 support (PDF#32-0469)), respectively, in the reciprocal space. **b-c** STEM images of iron fluoride-oxide nanoporous film with ultra-small interconnected crystalline nanoparticles. Scale bar, **a-c** 5 nm.

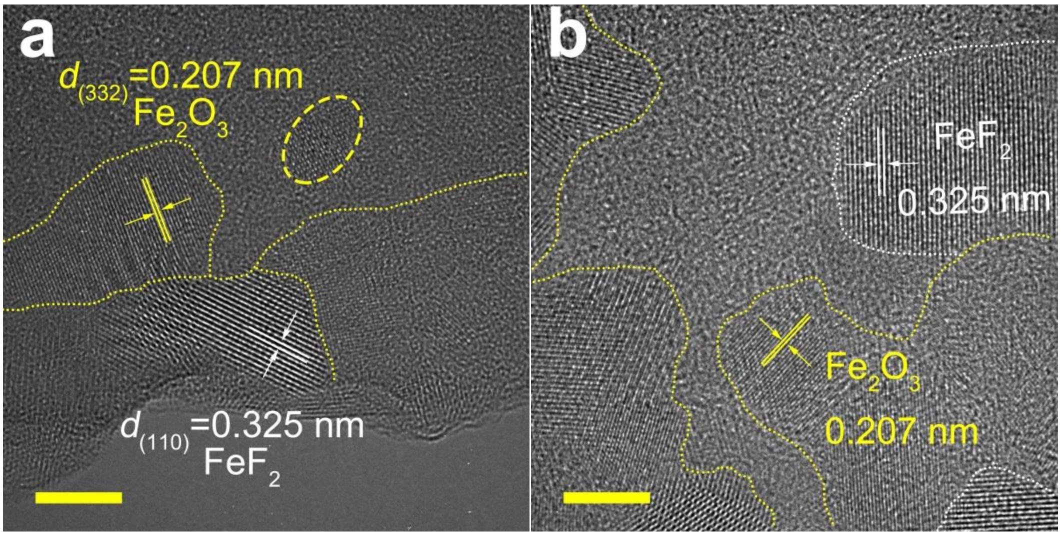

Supplementary Figure 17 | TEM images of IFONFs-30. a TEM image of IFONFs with small nanodomains. **b** HRTEM images of IFONFs, which further reveal the iron fluoride-oxide formed with fluorination prefer to expose its edges, leading to a maximal exposure of the edge sites on the substrate. Scale bar, **a,b** 5 nm.

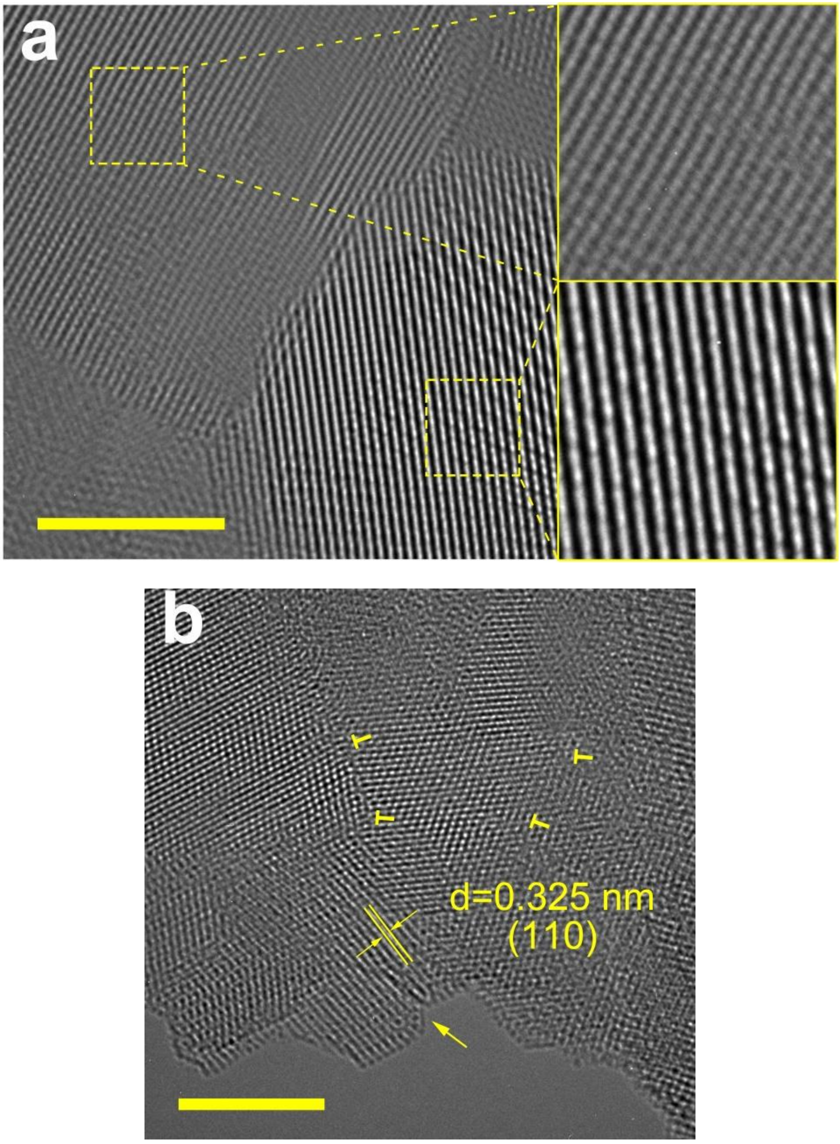

Supplementary Figure 18 | STEM images IFONFs-45. a The results of the STEM structural show that iron fluoride-oxide nanoporous film are made of two clearly distinct structural domains consisting of FeF_2 (right top) and Fe_2O_3 (right bottom), and they still maintain the original two-dimensional arrangement and electron conjugated system. **b** The presence of defects regions is also verified by a yellow arrow. Scale bar, **a,b** 5 nm.

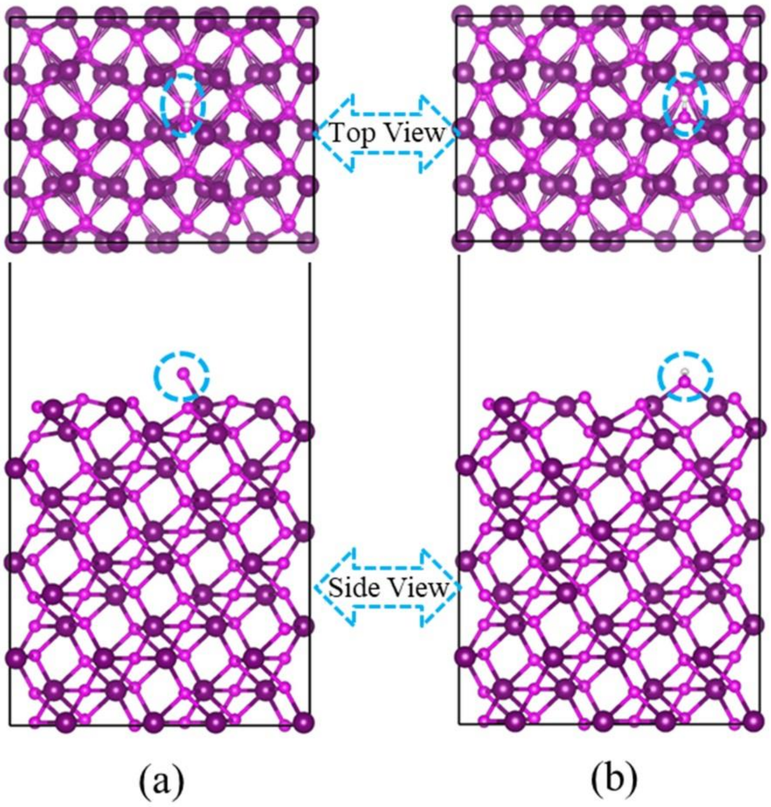

Supplementary Figure 19 | Theoretical calculations of Fe_2O_3 surface. Top and side views of OH adsorption on **a** the perfect Fe_2O_3 (100) surface, **b** the surface with one O vacancy. The OH groups are marked by blue circles. We selected (100) surface because it is the most stable and active surface[2]. We used a 2×2 supercell with ~15 Å vacuum layer, and the spin polarized DFT+U formalism with the U value of 4.3 eV for Fe[3]. The energy cut-off for the plane waves was set to 400 eV, and all atomic positions were fully relaxed with a Γ point until the final force on each atom was less than 0.01 eV/Å. Indeed, our calculations show that the adsorption energy is facilitated by 0.99 eV at the O vacancy, which is also advantageous for electrocatalysis.

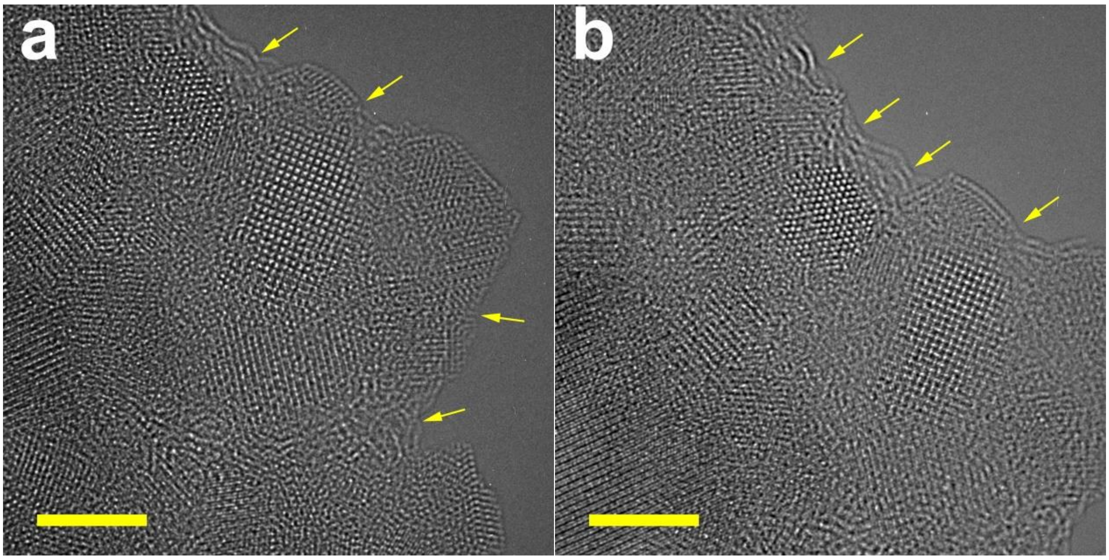

Supplementary Figure 20 | TEM characterization of IFONFs-45. a,b HRTEM images of typical iron fluoride-oxide nanoporous film with nanocracks. Scale bar, **a,b** 5 nm.

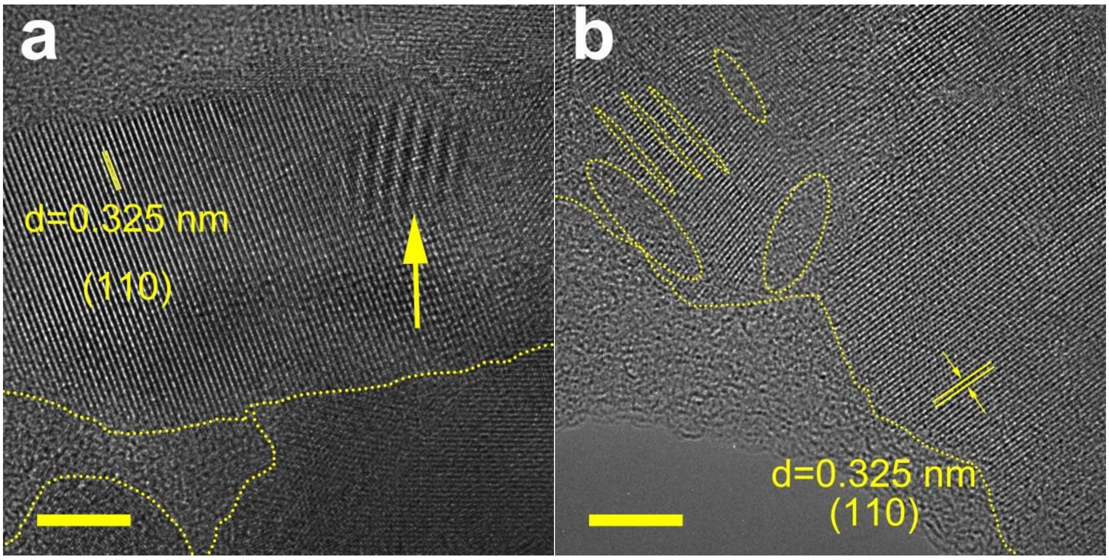

Supplementary Figure 21 | TME characterizations the IFONFs prepared at 350 °C for fluorinated with 45 min. a FeF_2 layered crystals are formed with fluorination, which gradually crystallize into larger FeF_2 nanodomains (45 min) with edge-oriented structure. The slight rotations that are marked with yellow arrows can be observed in the HRTEM image of iron fluoride-oxide. **b** HRTEM image shows discontinued crystal fringes (indicated by the dotted circles) with interlayer distance of about 0.325 nm, corresponding to the (110) plane of FeF_2 structure, indicating the presence of large amount structure distortion in the iron fluoride-oxide nanoporous film. Scale bar, **a,b** 5 nm.

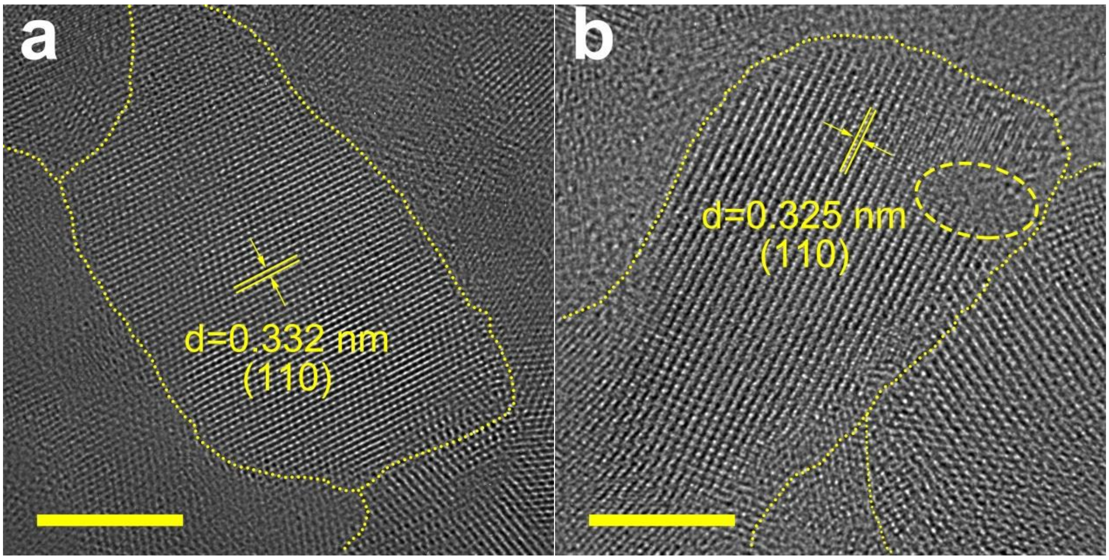

Supplementary Figure 22 | STEM characterization of IFONFs-45. a,b STEM images of IFONFs fluorinated with 60 min. Scale bar, **a,b** 5 nm.

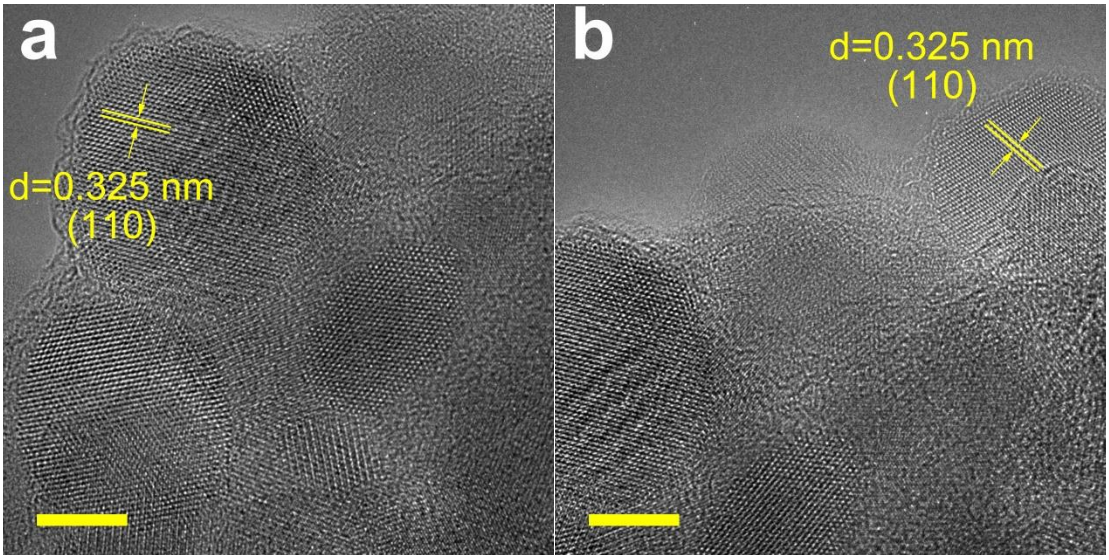

Supplementary Figure 23 | STEM characterizations of IFONFs fluorinated with 90 min. a,b The STEM images clearly show that the iron fluoride-oxide composites with big nanodomains and FeF_2 NCs are distributed with aggregated structure on the iron fluoride-oxide nanoporous film. Scale bar, **a,b** 5 nm.

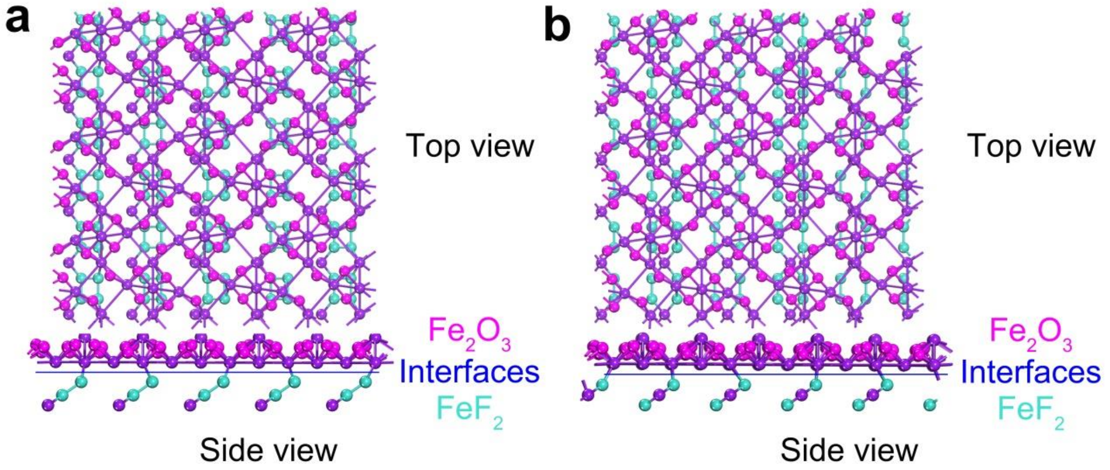

Supplementary Figure 24 | Structural models of FeF_2-F_2O_3 hybrid. a,b Different FeF_2 (101) surfaces with fluorine termination of FeF_2-Fe_2O_3 hybrids are got by cutting with different depths of bulk FeF_2. The F atoms of FeF_2 bond with Fe atoms in the Fe_2O_3 matrix on FeF_2-F_2O_3 heterostructures. Turquoise = F, purple = Fe, magenta = O. Fe_2O_3 (400) surface with iron termination is combined with FeF_2 (101) surface with fluorine termination in FeF_2-F_2O_3 hybrid.

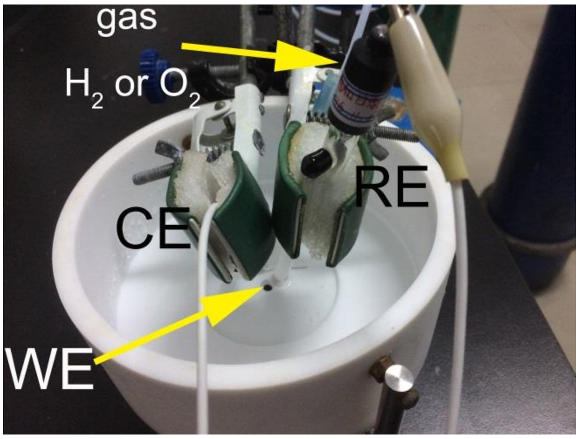

Supplementary Figure 25 | Optical image of 2-compartment three-electrode electrochemical cell. The working electrode (WE), counter electrode (CE), and the reference electrode (RE) are immerged in the water electrolysis and connected to the potentiostat for electrolysis characterization. Saturated calomel electrode (SCE) and platinum wire are used as RE and CE, respectively. A 3 mm dia polyethylene tube is used for bubbling the gas (H_2 or O_2) into the solution for required time.

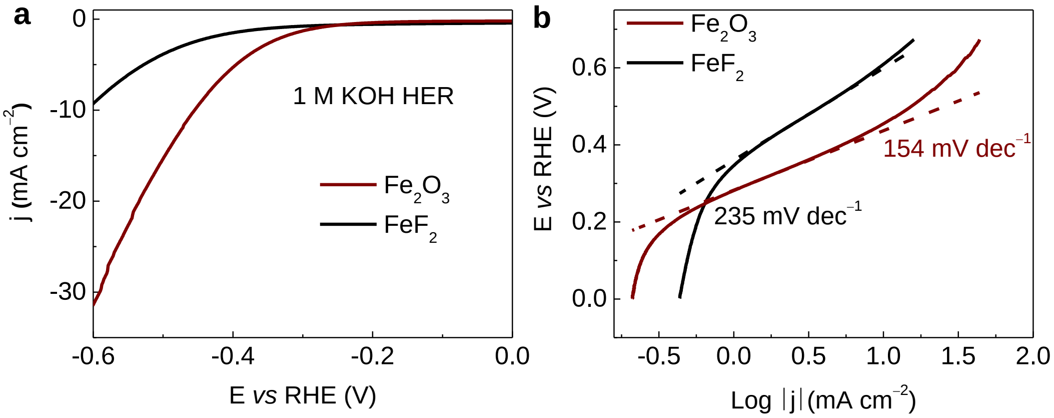

Supplementary Figure 26 | HER activity of Fe_2O_3 and FeF_2 PTF. a HER polarization curves and **b** the corresponding Tafel plots of Fe_2O_3 and FeF_2 PTF, respectively.

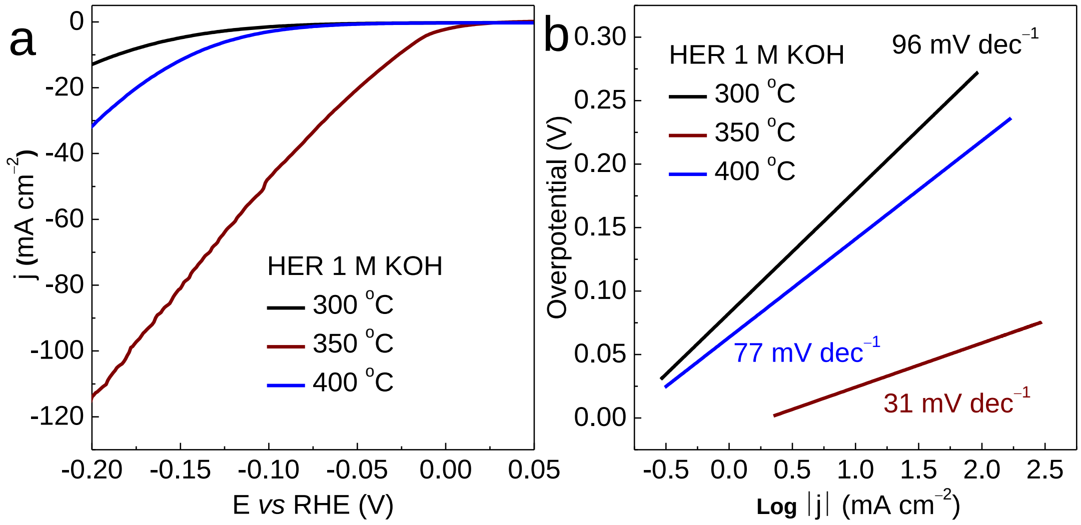

Supplementary Figure 27 | HER performance of IFONFs. a HER polarization curves and **b** the corresponding Tafel plots of IFONFs fluorinated at various temperature for 45 min, respectively.

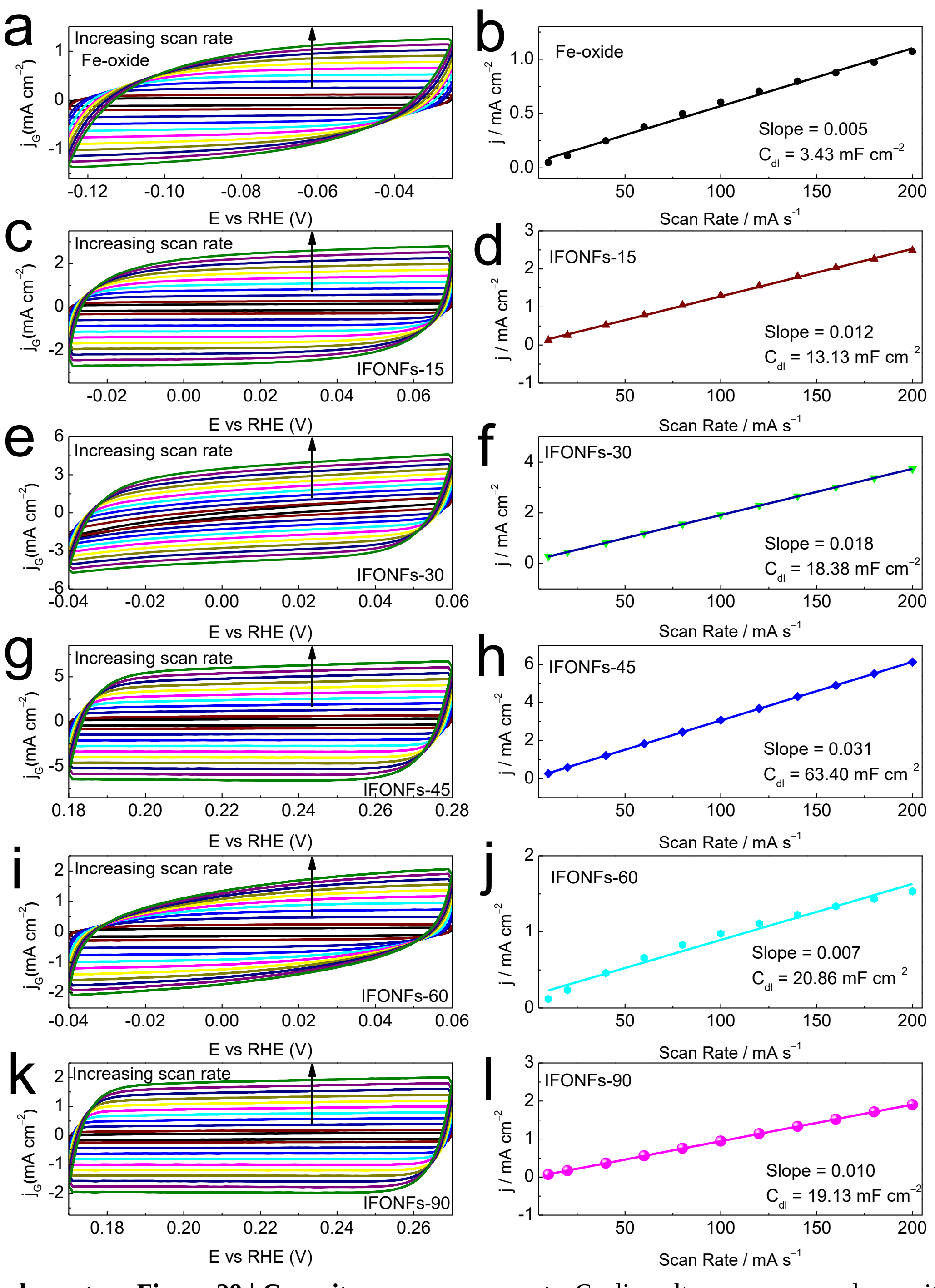

Supplementary Figure 28 | Capacitance measurements. Cyclic voltammograms and capacitive currents plotted as a function of scan rate in 1 M KOH at scan rates of 10, 20, 40, 60, 80, 100, 120, 140, 160, 180, and 200 mV s^{-1} for **a-b** the raw Fe-oxide PTF and IFONFs prepared with various time, **c-d** 15 min, **e-f** 30 min, **g-h** 45 min, **i-j** 60 min, and **k-l** 90 min, respectively, where no obvious electrochemical features corresponding to the Faradic current are observed.

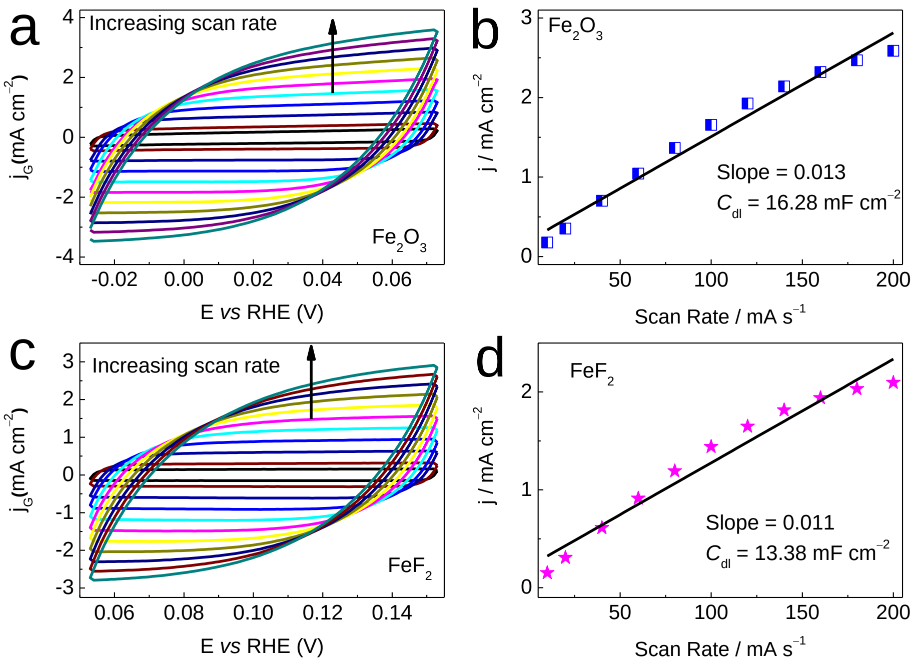

Supplementary Figure 29 | CV curves and ECSA of Fe_2O_3 and FeF_2 PTF. Cyclic voltammograms and capacitive currents plotted as a function of scan rate in 1 M KOH at scan rates of 10, 20, 40, 60, 80, 100, 120, 140, 160, 180, and 200 mV s^{-1} for **a-b** Fe_2O_3 PTF, **c-d** FeF_2 PTF.

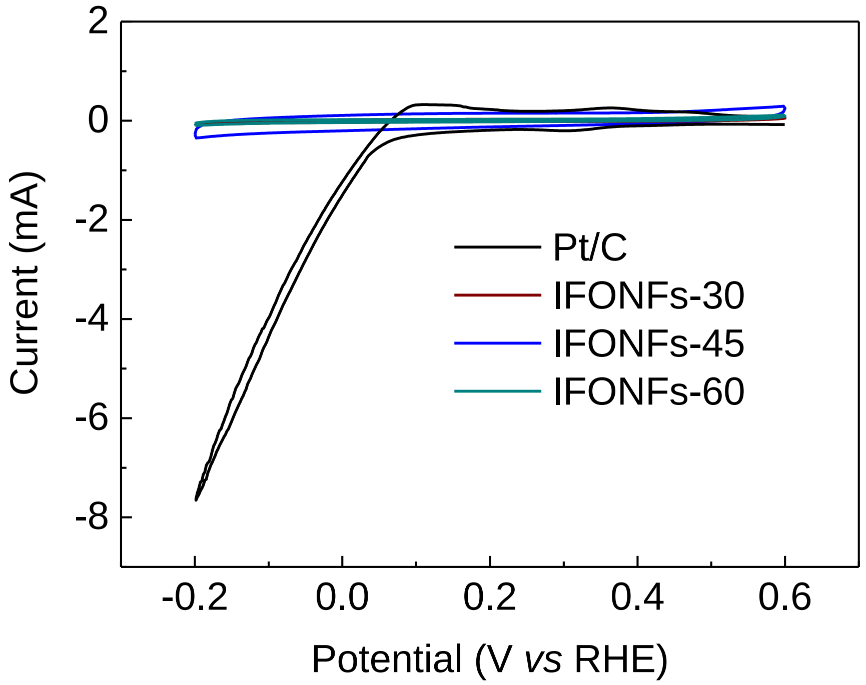

Supplementary Figure 30 | CVs of IFONFs-45 and Pt/C. CVs of IFONFs-45 and Pt/C in 1 M KOH (pH 14) with a scan rate of 50 mV s^{-1} in the region of −0.2 to 0.6 V *vs* RHE.

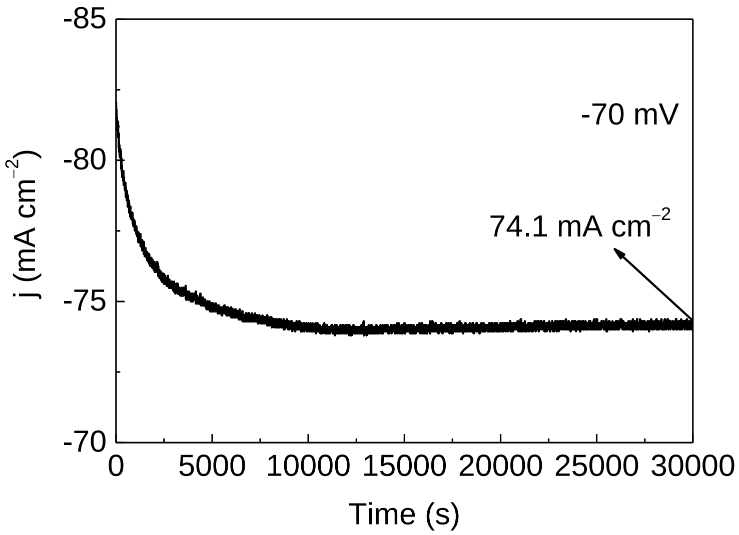

Supplementary Figure 31 | Stability test of Pt/C. Time-dependent current density curve of Pt/C at a fixed overpotential of -70 mV to drive 82 mA cm^{-2} for 30000 s in 1 M KOH.

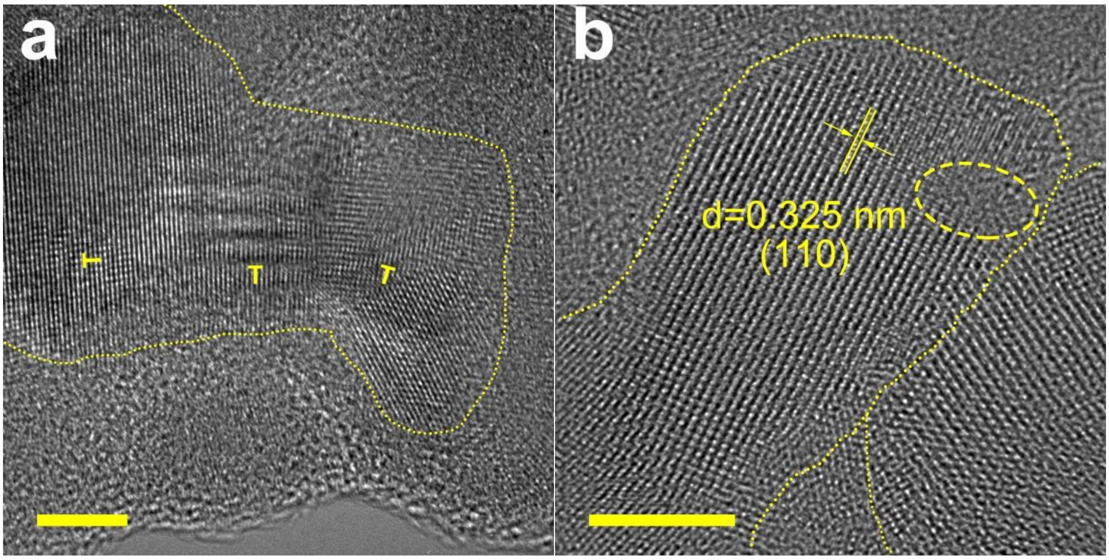

Supplementary Figure 32 | STEM characterizations of IFONFs-45 electrode. a,b STEM images of IFONFs-45 after 3000 cycles HER testing. Scale bar, **a,b** 5 nm.

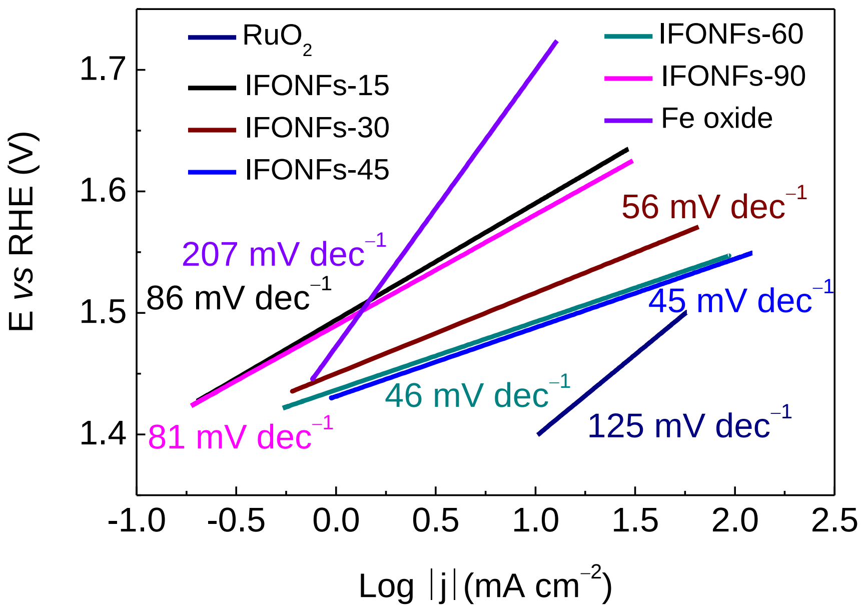

Supplementary Figure 33 | Tafel slopes of RuO_2, Fe-oxide and IFONFs hybrid catalysts.

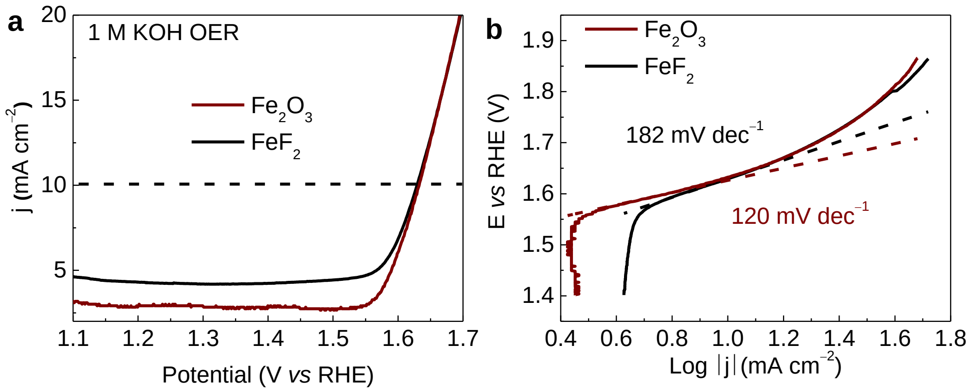

Supplementary Figure 34 | OER activity of Fe_2O_3 and FeF_2 PTF. a OER polarization curve and **b** the corresponding Tafel plot of Fe_2O_3 and FeF_2 PTF.

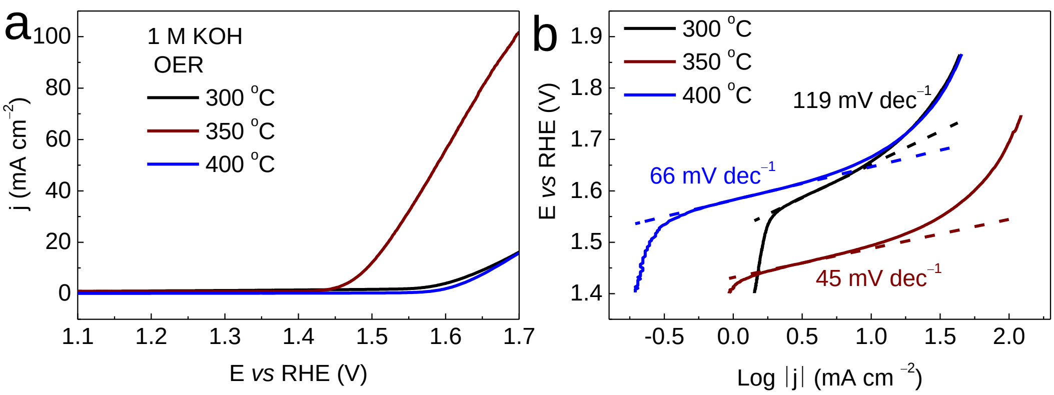

Supplementary Figure 35 | OER activity of IFONFs. a OER polarization curves and **b** the corresponding Tafel plots of IFONFs fluorinated at various temperature, respectively.

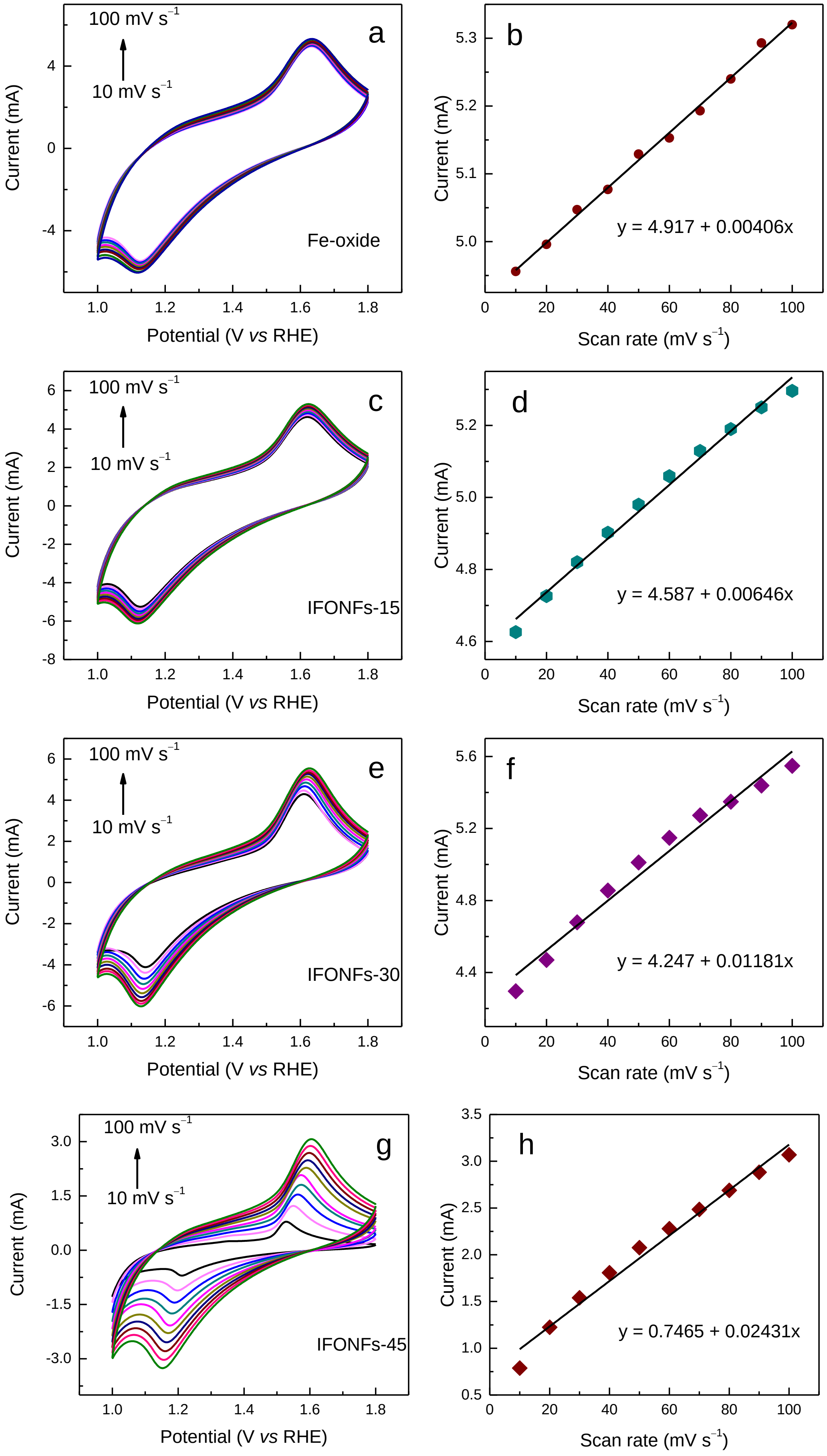
a
100 mV s−1
10 mV s−1
Fe-oxide
Current (mA)
Potential (V vs RHE)
b
y = 4.917 + 0.00406x
Current (mA)
Scan rate (mV s−1)
c
100 mV s−1
10 mV s−1
IFONFs-15
Current (mA)
Potential (V vs RHE)
d
y = 4.587 + 0.00646x
Current (mA)
Scan rate (mV s−1)
e
100 mV s−1
10 mV s−1
IFONFs-30
Current (mA)
Potential (V vs RHE)
f
y = 4.247 + 0.01181x
Current (mA)
Scan rate (mV s−1)
g
100 mV s−1
10 mV s−1
IFONFs-45
Current (mA)
Potential (V vs RHE)
h
y = 0.7465 + 0.02431x
Current (mA)
Scan rate (mV s−1)

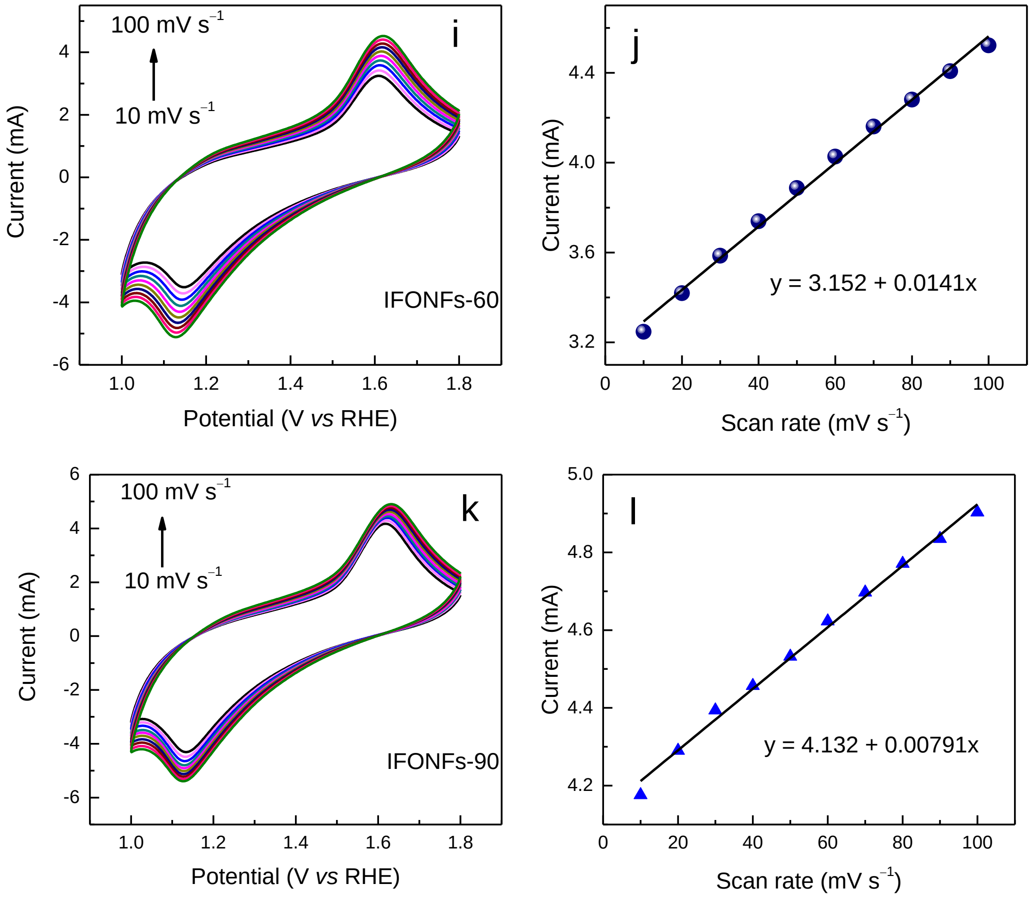

Supplementary Figure 36 | Calculation for the number of active sites. a, **c**, **e**, **g**, **I** and **k** Cyclic voltammograms of Fe-oxide PTF and IFONFs hybrids under different scan rates increasing from 10 to 100 mV s^{-1} in 1 M KOH. **b**, **d**, **f**, **h**, **j** and **l** Linear relationship of the peak current for the oxidation wave at the scan rate.

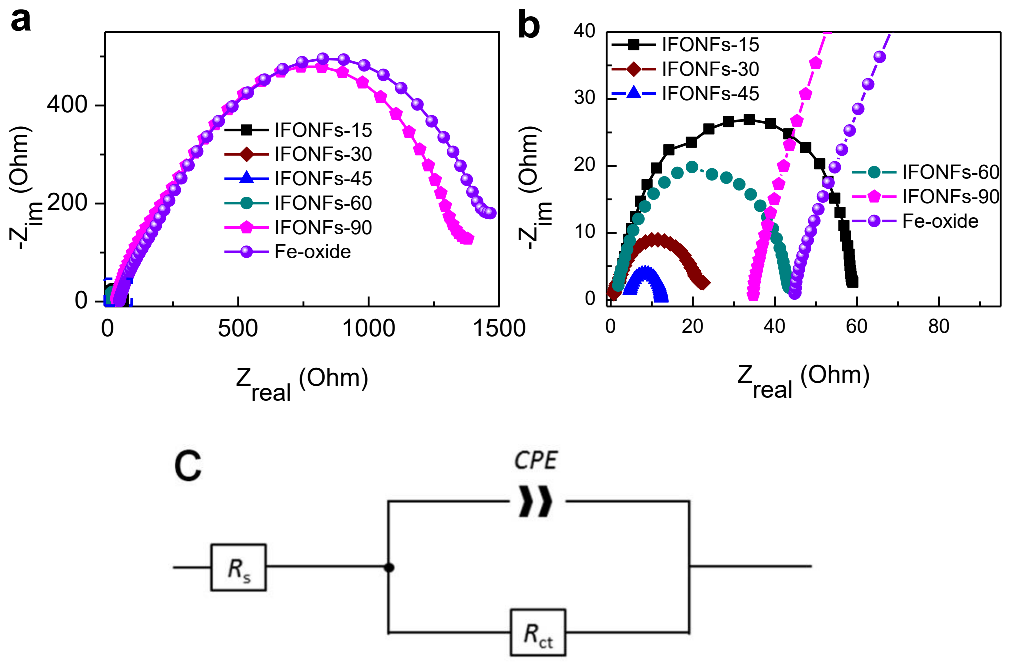

Supplementary Figure 37 | Impedance studies of Fe-oxide PTF and IFONFs. a EIS data for Fe-oxide PTF and IFONFs hybrids in 1 M KOH under OER overpotential of 500 mV, **b** the enlargement of area denoted by dash squares in **a, c** the equivalent circuit model for the impedance spectroscopy.

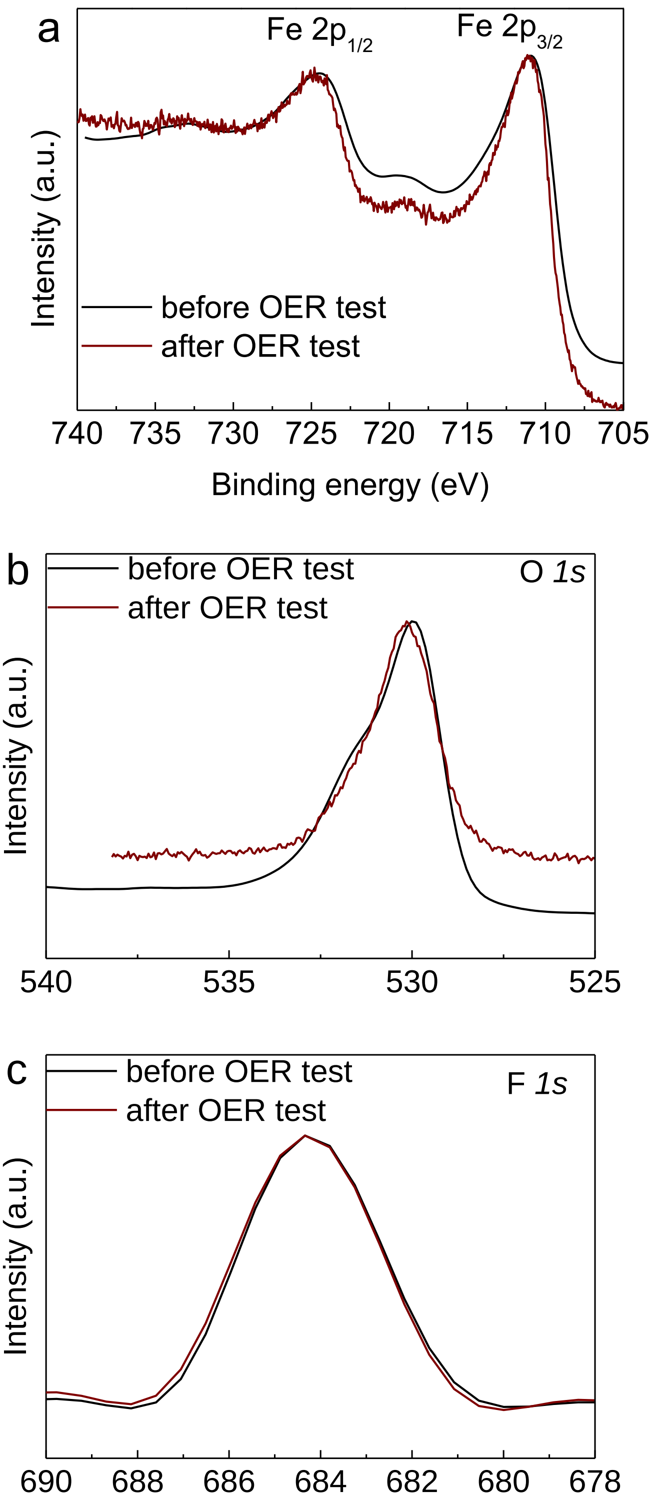

Supplementary Figure 38 | XPS spectra of IFONFs-45 before and after OER durability test. High-resolution XPS in the **a** Fe *2p*, **b** O *1s*, and **c** F *1s* regions of IFONFs-45 before and after 100000 s OER durability test.

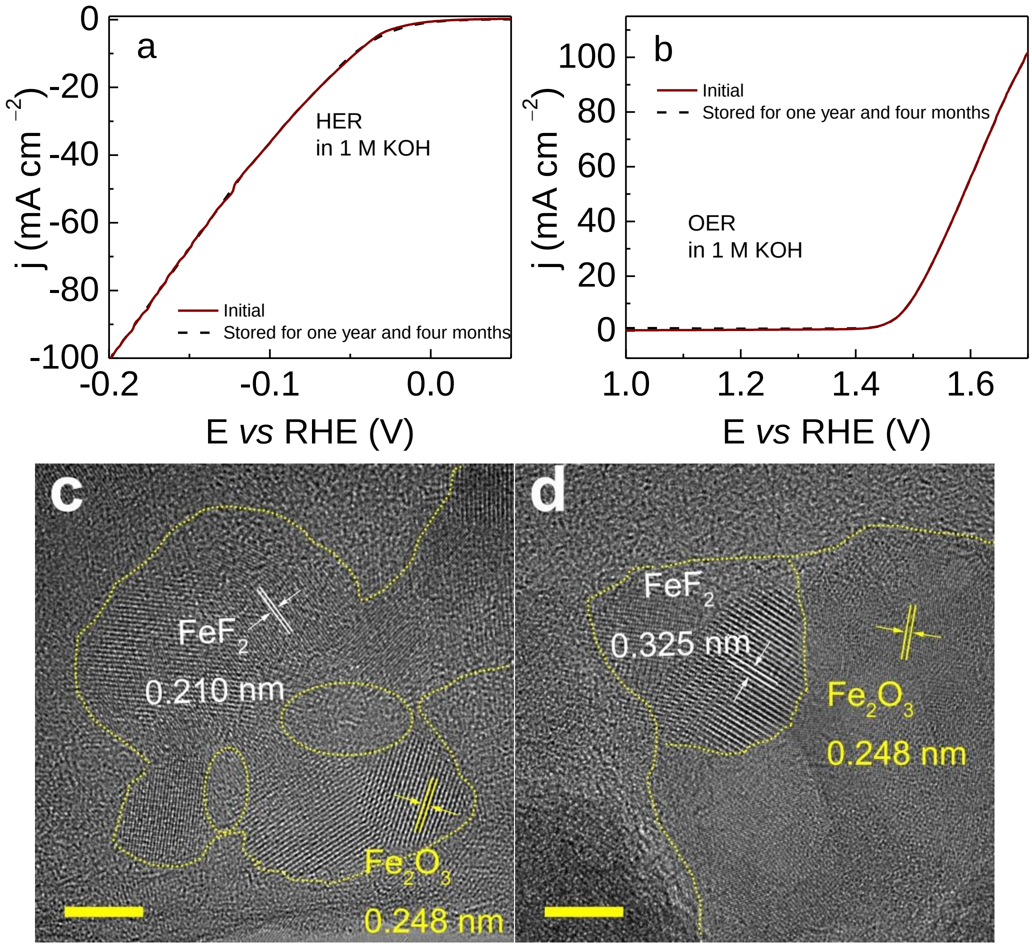

Supplementary Figure 39 | Chemical stability of IFONFs-45 electrode. LSV curves of IFONFs-45 electrode before (red curve) and after stored under ambient conditions for one year and four months (blak dash curve) for **a** HER and **b** OER, respectively. **c,d** HRTEM images for IFONFs-45 stored under ambient conditions for one year and four months. Scale bar, **c,d** 5 nm.

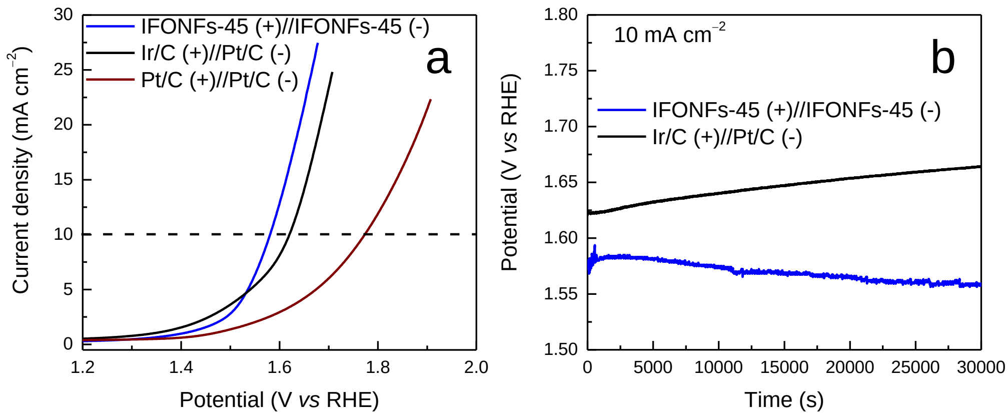

Supplementary Figure 40 | Water splitting performance characterization. a Polarization curves of IFONFs-45 (+ and −), Pt/C (+)∥Pt/C (−), and Ir/C (+)∥Pt/C (−) for overall water splitting in a two-electrode configuration (not iR-corrected). **b** Chronopotentiometry curves of IFONFs-45 and Ir/C (+)∥Pt/C (−) under a current density of 10 mA cm^{-2} without iR correction. All experiments were carried out in 1 M KOH.

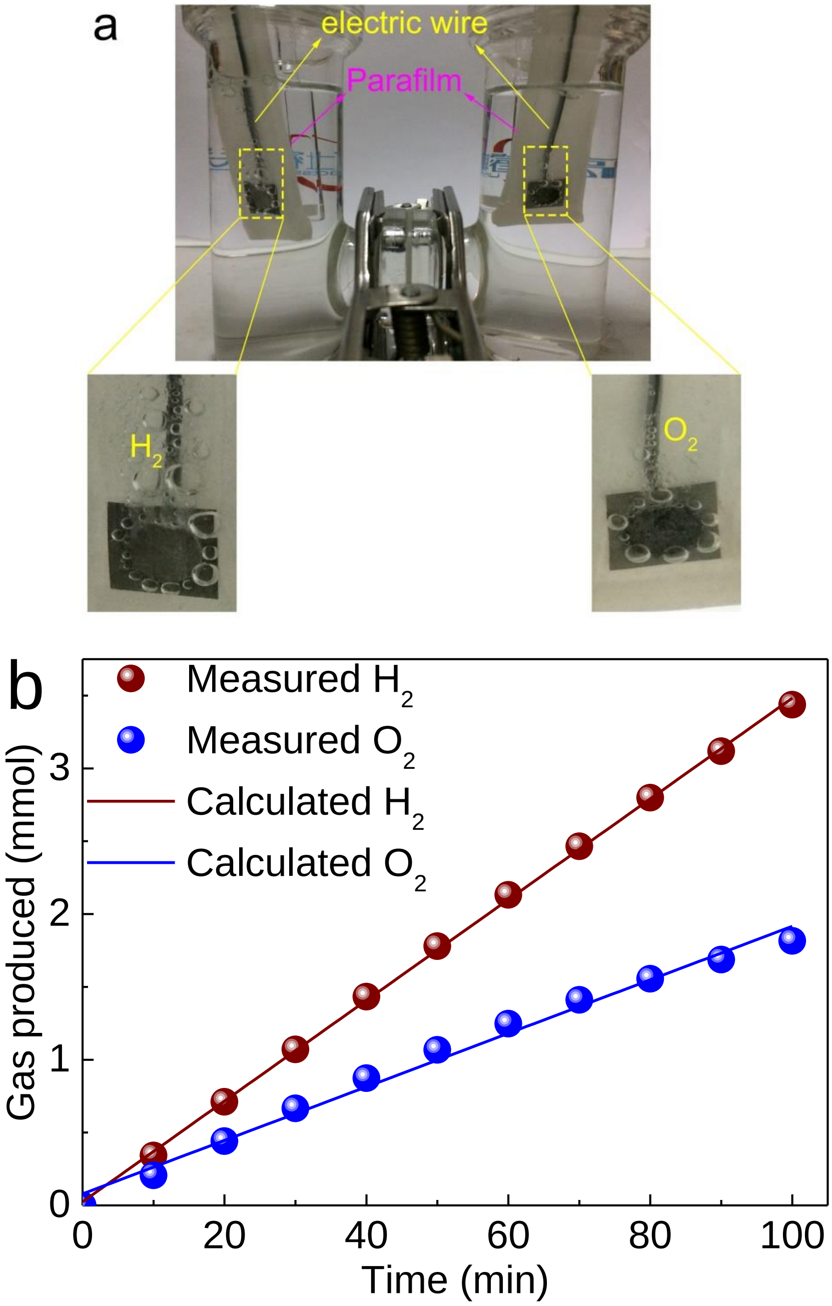

Supplementary Figure 41 | Faradic efficiencies calculations for both HER and OER. a Image showing the evolution of H_2 and O_2 gas on IFONFs-45 in a well-sealed H-type cell. The IFONFs-45 samples are sealed with parafilm using electric wires connected with electrochemical workstation. **b** Experimental and theoretical amounts of H_2 and O_2 by the IFONFs-45 electrode at a fixed current density of 40 mA cm^{-2}.

Supplementary Table 1. Fe content determined by ICP-MS and quantitative surface analysis by XPS.

Samples	Fe loading (wt%)	Surface atomic concentration (at%)			
		C	O	F	Fe
**Fe-oxide**	21.5	37.17	45.62	-	17.21
**IFONFs-15**	19.3	35.76	39.85	6.76	17.63
**IFONFs-30**	19.2	36.10	37.51	9.34	17.05
**IFONFs-45**	18.8	35.42	33.76	12.45	18.37
**IFONFs-60**	18.6	35.30	33.17	14.26	17.27
**IFONFs-90**	14.8	35.42	30.38	16.32	17.88

Fe-oxide PTF has a higher Fe content of 21.5 at% derived from the ICP-MS measurements. The surface O content decreases significantly from 45.62 to 30.38-39.85 at% from XPS measurements.

Supplementary Table 2. Comparison of HER and OER activity data among various catalysts.

Catalysts	Reactions	Tafel slope mV dec^{-1}	η_{10} mV	E_{onset} mV	j@200 mV mA cm^{-2}	j_0 mA cm^{-2}	TOF* s^{-1}
**Fe-oxide**	HER	209	505	94	0.6	0.0016	0.00374
	OER	207	1.71	1.42	-	-	0.0141
**Pt/C**	HER	30	13.7	0	610.0	0.7100	1.19000
**RuO_2**	OER	125	1.40	1.29	-	-	-
**IFONFs-15**	HER	96	269	85	2.3	0.0012	0.00259
	OER	86	1.61	1.44	-	-	0.0319
**IFONFs-30**	HER	68	219	63	5.7	0.0470	0.00244
	OER	56	1.52	1.43	-	-	0.1156
**IFONFs-45**	HER	31	47	20	101.4	0.0950	0.27700
	OER	45	1.49	1.39	-	-	0.2141
**IFONFs-60**	HER	55	92	40	84.9	0.0322	0.09588
	OER	46	1.51	1.40	-	-	0.1745
**IFONFs-90**	HER	63	100	55	62.0	0.0260	0.07740
	OER	81	1.61	1.42	-	-	0.0346
**Fe_2O_3**	HER	154	456	124	0.4	-	-
	OER	120	1.63	1.54	-	-	-
**FeF_2**	HER	235	610	147	0.3	-	-
	OER	182	1.64	1.45	-	-	-

* Note that the TOF for HER measured at η = 100 mV, for OER measured at η = 1.6 V, respectively.

Supplementary Table 3. Comparison of HER activity of Fe- and some non-Fe based catalysts.

Catalysts	Electrolyte	Tafel slope mV dec^{-1}	Onset overpotential mV	η_{10} mV	Metal precursor	ref
**IFONFs-45**	**1 M KOH**	**31**	**20**	**47**	**Fe foil**	**This work**
FeP nanorod arrays	1 M KOH	146	86	218	Fe_2O_3 nanorod arrays	*ACS Catal.*, 2014[4]
FeP	1 M KOH	75	-	194	Fe_2O_3 nanowires	*Chem. Commun.*, 2016[5]
FeP_2	1 M KOH	67	-	189	Fe_2O_3 nanowires	*Chem. Commun.*, 2016[5]
Porous Ni–Fe–P@C NRs	1 M KOH	92.6	~0	79	Iron(III) nitrate nonahydrate	J. Mater. Chem. A, 2017[6]
$Fe_{0.1}NiS_2$ NA/Ti	1 M KOH	108	-	η_{20} = 243	$Fe(NO_3)_3$ $6H_2O$	*Nano Res.*, 2016[7]
NiFe-LDH NA/Ti	1 M KOH	124	-	η_{20} = 476	$Fe(NO_3)_3$ $6H_2O$	*Nano Res.*, 2016[7]
Ni_3Fe LDHs/NF	1 M KOH	75	-	45	$Fe(NO_3)_3$ $9H_2O$	*ACS Appl. Mater. Interfaces*, 2016[8]
NiFe/NF	1 M KOH	112	-	139	$FeSO_4$ $7H_2O$	*Int. J. Hydrogen Energ.*, 2016[9]
Iron phosphide nanotubes (IPNTs)	1 M KOH	59.5	31	120	$(Fe(NO_3)_3 \cdot 9H_2O$	*Chem.–Eur. J.*, 2015[10]
EG/$Co_{0.85}$Se/NiFe-LDH	1 M KOH	57	240	260	$Fe(NO_3)_3 \cdot 9H_2O$	*Energ. Environ. Sci.*, 2016[11]
NiFe LDH-NS@DG10	1 M KOH	110	-	300	$Fe(NO_3)_3$ $9H_2O$	*Adv. Mater.*, 2017[12]
Ru@C_2N^*	1 M KOH	38	-	17	$RuCl_3$, $NaBH_4$	*Nat. Nano.*, 2017[13]
2.5H-PH NCMs*	1 M KOH	38.1	-	70	$(NH_4)_2MoS_4$	*Nat. Commun.*, 2017[14]
HNDDC-	1 M KOH	93.4	-	158	$Co(CH_3COO)_2$	*Nat.*

100,000-1,000/Co*						*Commun.*, 2017[15]
3.0 % S-CoO NRs*	1 M KOH	82	-	73	CoO	*Nat. Commun.*, 2017[16]
RuCo@NC (S-4)*	1 M KOH	31	-	28	$RuCl_3$	*Nat. Commun.*, 2017[17]

*Non-Fe based catalysts.

Supplementary Table 4. Comparison of OER activity of Fe- and some non-Fe based catalysts.

Catalysts	Electrolyte	Tafel slope mV dec^{-1}	Onset overpotential V	η_{10} V	Metal precursor	ref
**IFONFs-45**	**1 M KOH**	**45**	**1.39**	**1.49**	**Fe foil**	**This work**
[Ni,Fe]O	0.1 M KOH	36–48	-	300 mV	Metal chlorides	*ACS Appl. Mater. Interfaces*, 2015[18]
FeP–rGO (70 : 30)@Au	1 M KOH	49.6	1.44	290 mV	Trioctylphospine oxide (TOPO) and Trioctylphosphine (TOP)	*J. Mater. Chem. A*, 2016[19]
Iron phosphide nanotubes (IPNTs)	1 M KOH	43	1.48	1.52	$Fe(NO_3)_3$ $9H_2O$	*Chem.–Eur. J.*, 2015[10]
FeP NAs/CC	1 M KOH	146	86 mV	218 mV	Fe_2O_3 nanorod arrays	*ACS Catal.*, 2014[4]
De-LNiFeP/rGO	1 M KOH	33.6	1.47	1.50	$Fe(NO_3)_3$	*Energ. Environ. Sci.*, 2015[20]
Fe-Ni oxides	1 M KOH	51	-	-	Iron nitrate	*ACS Catal.*, 2012[21]
Ni-Fe LDH/CNT	1 M KOH	31	1.45	1.48	Ferrous nitrate ($Fe(NO_3)_3$)	*J. Am. Chem. Soc.*, 2013[22]
FeNi-rGO LDH	1 M KOH	39	1.44	1.436	Ferrous chloride ($FeCl_3$)	*Angew. Chem. Int. Ed.*, 2014[23]
NiFe LDHs	1 M KOH	40	-	300 mV	Iron nitrate ($Fe(NO_3)_3$ $9H_2O$	*Nat. Commun.*, 2014[24]
$Fe_6Ni_{10}O_x$	1 M KOH	48	-	286 mV	$Fe(NO_3)_3$	*Angew. Chem. Int. Ed.*, 2014[25]
Porous Ni–Fe–P@C NRs	1 M KOH	40	1.43	217 mV	Iron(III) nitrate nonahydrate	*J. Mater. Chem. A*, 2017[6]
$Fe_{0.1}NiS_2$ NA/Ti	1 M KOH	43	-	η_{100} = 231 mV	$Fe(NO_3)_3$ $6H_2O$	*Nano Res.*, 2016[7]
NSPM-Ni_3FeN/NF	1 M KOH	40	-	1.495	Iron nitrate $Fe(NO_3)_3$ $9H_2O$	*ACS Appl. Mater. Interfaces*, 2016[8]
NiFe/NF	1 M KOH	51	1.58	1.64	$FeSO_4$ $7H_2O$	*Int. J. Hydrogen Energ.*, 2016[9]
NiFe-LDH NA/Ti	1 M KOH	117	-	η_{100} = 431	$Fe(NO_3)_3$ $6H_2O$	*Nano Res.*, 2016[7]

				mV		
EG/$Co_{0.85}Se$/NiFe-LDH	1 M KOH	57	1.47	1.67	$Fe(NO_3)_3$ $9H_2O$	*Energ. Environ. Sci.*, 2016[11]
NiFe LDH-NS@DG10	1 M KOH	52	1.41	210 mV	$Fe(NO_3)_3$ $9H_2O$	*Adv. Mater.*, 2017[12]
$PrBa_{0.5}Sr_{0.5}Co_{1.5}Fe_{0.5}O_{5+\delta}$ (PBSCF-III)	0.1 M KOH	52	-	358 mV	$Fe(NO_3)_3$ $9H_2O$	*Nat. Commun.*, 2017[26]
2.5H-PH NCMs*	1 M KOH	45.7	-	1.465	$(NH_4)_2MoS_4$	*Nat. Commun.*, 2017[14]
Ni-NHGF*	1 M KOH	63	1.43	1.56	$NiCl_2$ $6H_2O$	*Nat. Catal.*, 2018[27]
HNDDC-100,000-1,000/Co*	1 M KOH	66.8	-	199 mV	$Co(CH_3COO)_2$	*Nat. Commun.*, 2017[15]

*Non-Fe based catalysts.

Supplementary Table 5. Comparison of HER and OER activity data among various catalysts.

Catalysts		Reactions	Tafel slope mV dec^{-1}	η_{10} mV	η_{100} mV	E_{onset} mV	ref
Fluoride	**IFONFs-45**	**HER**	**31**	**47**	**199**	**20**	**This work**
		**OER**	**45**	**1.49**	**1.69**	**1.39**	
Borides	FeB_2	HER	87.5	61	200*	20	28
		OER	52.4	296	1.63*	1.48	
	Fe_2B	HER	102.4	138	250*	-	28
		OER	78.7	-	1.65*	1.54	
Sulfides	$Fe_{0.1}NiS_2$ NA/Ti	HER	108	250*	350*	-	7
		OER	43	205*	231	-	
	$(Ni_{0.75}Fe_{0.25})Se_2$	HER	NA	NA	NA	NA	29
		OER	47.2	η_{35} =255	277	-	
Phosphides	FeP NAs/CC	HER	146	218	275*	86	4
		OER	NA	NA	NA	NA	
	FeP NWs	HER	75	194	NA	-	5
		OER	NA	NA	NA	NA	
	FeP_2 NWs	HER	67	189	NA	-	
		OER	NA	NA	NA	NA	
	Iron phosphide nanotube (IPNTs)	HER	59.5	120	180*	31	10
		OER	43	1.52	1.57*	1.48	
Nitrides/ Selenides	Nanoporous Fe_xN	HER	NA	NA	NA	NA	30
		OER	44.5	-238	1.53*	-	
	Co_3FeN_x	HER	94	23	147	-	31
		OER	46	η_{20}=222	253	-	
	Ni_3FeN-NPs	HER	42	158	310*	-	32
		OER	46	280	1.62*	-	
Carbides	Fe_3C@NG800-0.2	HER	NA	NA	NA	NA	33
		OER	62	1.59	1.61*	-	
	IP-IC@SWNT(P)	HER	87.6	301	NA	-	34
		OER	NA	NA	NA	NA	
	Fe@C-NG/NC NTs	HER	NA	NA	NA	NA	35
		OER	163	1.68	η_{20} =1.72*	-	

* The value is calculated from the curves shown in the literatures.

Supplementary Note 1: Synthesis of IFONFs.

Fe foils (0.05 mm, 99.5%, Advent Materials, UK) were used as substrates and cleaned and degreased by sonication in 2-propanol (~99.7%) and acetone (~99.9%) before use. Electrochemical anodic treatment was carried out at room temperature in a solution of 0.1 M NH_4F (98%, Alfa Aesar, USA) with 1 M deionized water in ethylene glycol (~99.9%) to grow Fe-oxide nanoporous layers on the Fe foils. Anodization was conducted at 40 V for 40 min in a two-electrode setup with platinum foil as a counter electrode. To grow thicker layers, extended growth duration is suggested. After that, the samples were immersed in ethanol overnight to remove the residual organic electrolyte in the nanoporous layer followed by fluorination. To convert the porous Fe-oxide to a porous iron fluoride-oxide film, the reaction with fluorine vapor was performed in a CVD apparatus. The Fe-oxide PTF was placed in the middle of a standard 1 in. quartz tube furnace and heated to 300-400 ℃ in Ar (100 sccm) at 6.4 Torr for different time (*e.g.* 15, 30, 45, 60, and 90 min), while ammonium fluoride (NH_4F, ~5 g) in a porcelain boat as a fluoride doping source was placed at the upstream side of the furnace far away from the center of heat region about 5 cm or heated by an external heating source at ~250 ℃. The excess NH_4F was employed to ensure complete reaction between the Fe-oxide PTF and NH_4F. Ar (100 sccm) was used as a protective gas during the reaction and cooling process. At the same time, Fe_2O_3 PTF was prepared according our previous study by annealing at 350 ^oC for 3 h in air for comparison[36], and FeF_2 PTF was prepared through fluorination reaction with Fe_2O_3 PTF at 350 ^oC for 45 min.

Supplementary Note 2: Material characterization.

A JEOL 6500F SEM was used to investigate the morphology. A JEOL 2010 HRTEM was used to observe the morphologies and lattice fringes of the samples. The atomic resolution TEM and STEM structural characterizations of IFONFs were carried out with a probe-corrected Titan G2 60-300 (FEI, USA) and Titan ChemiSTEM (FEI, USA) at acceleration voltages of 300 kV and 200 kV, respectively. The crystal structure of the sample was evaluated using XRD analysis, which was performed by a Rigaku Dmax-2000 X-ray powder diffractometer with Cu Kα radiation (λ = 1.5418 Å). The operating voltage and current were kept at 40 kV and 40 mA, respectively. A semiquantitative estimation of the composition of the heterogeneous thin film was performed by MDI Jade 9 software. XPS was conducted on a PHI Quantera SXM scanning X-ray microscope. An Al anode at 25 W was used as an X-ray source with a pass-energy of 26.00 eV, 45 take off angle and a 100 μm beam size. A field-emission TEM (JEOL 2010F) with an imaging filter (Gatan GIF) was used at 200 kV to characterize the morphology and the particle size distribution of the synthesized products.

Supplementary Note 3: Electrode preparation and electrochemical characterization, HER and OER.

The IFONFs grown on Fe foil were directly used as working electrodes for the electrochemical measurements in a custom made two-compartment three-electrode electrochemical cell (**Supplementary Fig. 25)**. Water electrolysis to generate H_2 and O_2 were performed at room temperature using a three-electrode configuration (CHI 660E) with IFONFs, a platinum wire, and a saturated calomel electrode (SCE) as working electrode, counter electrode, and reference

electrode, respectively. The polarization curves were obtained in 1 M KOH (pH = 14) alkaline solution with a scan rate of 50 mV s^{-1} at room temperature. The potential, measured against a SCE electrode, was converted to the potential versus the reversible hydrogen electrode (RHE) according to $E_{vs\ \mathrm{RHE}} = E_{vs\ \mathrm{SCE}} + E^{\theta}{}_{\mathrm{SCE}} + 0.059$ pH. The presented current density was normalized to the geometric surface area. EIS were carried out at overpotential of -5 mV (*vs* RHE) with a frequency range of 10^{-2} to 10^{6} Hz with AC signal amplitude of 5 mV. Prior to all measurements, the electrochemical cell was purged with H_2 bubbles for 30 min. The practical operations of the stability and durability were examined by electrolysis at fixed potentials in 1 M KOH over extended periods and long-term potential cycling.

OER. The polarization curves were obtained in O_2-saturated 1 M KOH (pH = 14) alkaline solution with a scan rate of 5 mV s^{-1} at room temperature. EIS measurements were performed by applying an AC voltage with 5 mV amplitude in a frequency range from 10^{-2} to 10^{6} Hz and recorded at 500 mV *vs* RHE. The stability test was performed by electrolysis at fixed potentials in 1 M KOH.

Supplementary Note 4: Capacitance measurements and comparison of electrochemically active surface areas.

The electrochemically active surface area (ECSA) was estimated from the electrochemical double-layer capacitance of the nanoporous layers. The C_{dl} was determined from the CV curves measured in a potential range without redox processes according to the following equation:

$$C_{dl} = \frac{I_c}{\nu} \quad (1)$$

where C_{dl}, I_{c}, and v are the double-layer capacitance (F cm^{-2}) of the electroactive materials, charging current (mA cm^{-2}), and scan rate (mV s^{-1}). The ECSA is then calculated from the double-layer capacitance according to equation:

$$\mathrm{ECSA} = \frac{\mathrm{C}_{dl}}{\mathrm{C_s}} \quad (2)$$

Where, C_{s} is the capacitance of an atomically smooth planar surface of the material per unit area under identical electrolyte conditions. An average value of $C_{\mathrm{s}} = 40$ μF cm^{-2} is used in this work. The roughness factor ($\boldsymbol{R}_{\mathrm{F}}$) is then calculated by dividing the estimated ECSA by the geometric area of the electrode.

An alternative way is to calculate the double layer capacitance (C_{dl}) that is linearly proportional to effective active surface area and therefore can provide a relative comparison. The C_{dl} value can be estimated by plotting the J (J_{a}-J_{c}) at **Supplementary Fig. 28a-b** -0.075 V *vs* RHE, **c-d** 0.02 V *vs* RHE , **e-f** 0.01 V *vs* RHE, **g-h** 0.23 V *vs* RHE, **i-j** 0.01 V *vs* RHE, and **k-l** 0.22 V *vs* RHE against the scan rate, where the slope is double C_{dl}. The linear relationships are observed with the slopes twice the C_{dl} value. Accordingly, the C_{dl} values for Fe-oxide, IFONFs-15, IFONFs-30 IFONFs-45 IFONFs-60, and IFONFs-90 are calculated to be 3.43, 13.13, 18.38, 63.40, 20.86, and 19.13 mF cm^{-2}, respectively. Clearly, the IFONFs-45 shows the largest C_{dl}, demonstrating its more catalytically active sites that profit from the defect enriched structure.

Calculated ECSA and R_{F} for the raw Fe-oxide PTF and IFONFs prepared with various time.

Fe-oxide PTF:

$$A_{\mathbf{ECSA}}^{\text{Fe-oxide}} = \frac{3.43\ mF\ cm^{-2}}{40\ \mu\text{F cm}^{-2}\ per\ cm^2\ ECSA} = 85.8\ cm^2 ECSA\ (3)$$

$$\mathrm{R}_F^{\text{Fe-oxide}} = \frac{85.8\ cm^2 ECSA}{1\ cm^2} = 85.8\ (4)$$

IFONFs-15:

$$A_{\mathbf{ECSA}}^{\text{IFONFs-15}} = \frac{13.13\ mF\ cm^{-2}}{40\ \mu\text{F cm}^{-2}\ per\ cm^2\ ECSA} = 328.3\ cm^2 ECSA\ (5)$$

$$\mathrm{R}_F^{\text{IFONFs-15}} = \frac{328.3\ cm^2 ECSA}{1\ cm^2} = 328.3\ \ (6)$$

IFONFs-30:

$$A_{\mathbf{ECSA}}^{\text{IFONFs-30}} = \frac{18.38\ mF\ cm^{-2}}{40\ \mu\text{F cm}^{-2}\ per\ cm^2\ ECSA} = 459.5\ cm^2 ECSA\ \ (7)$$

$$\mathrm{R}_F^{\text{IFONFs-30}} = \frac{459.5\ cm^2 ECSA}{1\ cm^2} = 459.5\ \ (8)$$

IFONFs-45:

$$A_{\mathbf{ECSA}}^{\text{IFONFs-45}} = \frac{63.40\ mF\ cm^{-2}}{40\ \mu\text{F cm}^{-2}\ per\ cm^2\ ECSA} = 1585.0\ cm^2 ECSA\ \ (9)$$

$$\mathrm{R}_F^{\text{IFONFs-45}} = \frac{1585\ cm^2 ECSA}{1\ cm^2} = 1585.0\ \ (10)$$

IFONFs-60:

$$A_{\mathbf{ECSA}}^{\text{IFONFs-60}} = \frac{20.86\ mF\ cm^{-2}}{40\ \mu\text{F cm}^{-2}\ per\ cm^2\ ECSA} = 521.5\ cm^2 ECSA\ \ (11)$$

$$\mathrm{R}_F^{\text{IFONFs-60}} = \frac{521.5\ cm^2 ECSA}{1\ cm^2} = 521.5\ \ (12)$$

IFONFs-90:

$$A_{\mathbf{ECSA}}^{\text{IFONFs-90}} = \frac{19.13\ mF\ cm^{-2}}{40\ \mu\text{F cm}^{-2}\ per\ cm^2\ ECSA} = 478.3\ cm^2 ECSA\ \ (13)$$

$$\mathrm{R}_F^{\text{IFONFs-60}} = \frac{478.3\ cm^2 ECSA}{1\ cm^2} = 478.3\ \ (14)$$

In Supplementary Fig. 28, the C_{dl} values for Fe_2O_3 PTF and FeF_2 PTF are calculated to be 16.28 and 13.38 mF cm^{-2}, respectively.

Calculated ECSA and R_F for Fe_2O_3 PTF and FeF_2 PTF.

Fe_2O_3 PTF:

$$A_{\text{ECSA}}^{\text{Fe2O3}} = \frac{16.28\ mF\ cm^{-2}}{40\ \mu\text{F cm}^{-2}\ per\ cm^2\ ECSA} = 407\ cm^2 ECSA\ \ (15)$$

$$\mathrm{R}_F^{\text{Fe2O3}} = \frac{407\ cm^2 ECSA}{1\ cm^2} = 407\ \ (16)$$

FeF_2 PTF:

$$A_{\mathbf{ECSA}}^{\mathrm{FeF2}} = \frac{13.38\ mF\ cm^{-2}}{40\ \mu\mathrm{F\ cm^{-2}}\ per\ cm^2\ ECSA} = 334.5\ cm^2 ECSA \quad (17)$$

$$\mathrm{R}_F^{\mathrm{FeF2}} = \frac{334.5\ cm^2 ECSA}{1\ cm^2} = 334.5 \quad (18)$$

The exchange current density (j_0) was calculated using extrapolation methods. When the overpotential value is 0, the log [j] values for IFONFs-45, IFONFs-30 and IFONFs-60 are -1.19, -0.78, and -0.63, respectively. Based on Tafel equations, j_0 for IFONFs-45, IFONFs-30 and IFONFs-60 was calculated to be 0.0950, 0.0470, and 0.0322 mA cm^{-2}, respectively.

Supplementary Note 5: Calculation of TOF and the number of active sites **for HER.**

For rough estimation of the active surface site density and per-site turn over frequency (TOF) in the IFONFs hybrid catalyst, we suppose that the contribution of the defect state plays a dominant role. This is reasonable since the catalytic performance of defect enriched IFONFs hybrid is far better than that of less defect catalyst. According to this approach adopted by Jaramillo et al[37], we carried out a similar calculation method by considering the relative roughness factor of the catalyst, the geometry of a IFONFs surface, and the hydrogen evolution current density. As shown in **Fig. 4c** of main text, we have determined the specific capacitance C_{dl} of IFONFs-45 to be 63.40 mF cm^{-2}. And the C_{dl} can be directly used to estimate the relevant electrochemical active surface area (ECSA) by using the specific capacitance value for a flat electrode with real surface area 1 cm^2. We assume 40 μF cm^{-2} for a flat electrode for calculation the TOF values (**Supplementary Table 2**). Thus, the number of electrochemically effective surface sites on the IFONFs catalyst was calculated as the following:

$$\frac{\#\ Surface\ sites\ (catalyst)}{cm^2\ geometric\ area} = \frac{\#\ Surface\ sites\ (flat\ standard)}{cm^2\ geometric\ area} \times Roughness\ factor \quad (19)$$

To further get insights into the per-site TOF, the following formula is utilized:

$$TOF\ \text{per site} = \frac{\#\ Total\ Hydrogen\ Turn\ Overs\ /\ cm^2\ geometric\ area}{\#\ Surface\ sites\ (catalyst)\ /\ cm^2\ geometric\ area} \quad (20)$$

The total number of hydrogen turn overs is related to the current density, and is calculated based on the following conversion:

$$\#_{H_2} = \left(j\frac{mA}{cm^2}\right)\left(\frac{1\ Cs^{-1}}{1000\ mA}\right)\left(\frac{1\ mole^{-1}}{96485.3\ C}\right)\left(\frac{1\ mol\ H_2}{2\ mol\ e^{-1}}\right)\left(\frac{6.022\times10^{23} H_2\ molecules}{2\ mol\ \ H_2}\right) =$$

$$3.12\ \times\ 10^{25}\ \frac{H_2/s}{cm^2}\ \text{per} \frac{mA}{cm^2} \quad (21)$$

The upper limit of the number of active sites was calculated based on the hypothesis that all iron atoms in the IFONFs catalyst formed active Fe_2O_3-FeF_2 centers and all of them were accessible to the electrolyte. The real number of active and accessible iron sites should be considerably lower than the calculated value. The bulk iron content of IFONFs revealed by the ICP-MS measurement was about 19 wt%. The geometrical area of the electrode was 0.4 cm in diameter (~0.125 cm^2), and the mass of the electrode was found to be ~0.025 mg (measured based on the entire films) for each piece. So, the iron loading is equal to ~0.2 mg cm^{-2}. Accordingly, the upper limit of active site density is

$$\frac{19\ mg}{100\ mg} \times 0.2\ \frac{mg}{cm^2} \times \frac{1}{55.85}\ \frac{mmol}{mg} \times 6.022 \times 10^{20}\ \frac{sites}{mmol} = 0.410 \times 10^{18}\ sites\ cm^{-2}\ (22)$$

Accordingly, at an overpotential of 100 mV, the HER current density for Fe-oxide, IFONFs-15, IFONFs-30, IFONFs-45, IFONFs-60, and IFONFs-90 were 0.370, 0.340, 0.320, 36.4, 12.6, and 10.16 mA cm^{-2}, respectively, and the TOF value of Fe-oxide was calculated to be

$$\mathrm{TOF}_{\mathrm{Fe-oxide}} = \frac{3.12 \times 10^{15} \times \frac{H_2/_s}{cm^2}\ per\ \frac{mA}{cm^2} \times 0.492\ \frac{mA}{cm^2}}{0.410 \times 10^{18}\ sites\ cm^2} = 0.00374\ s^{-1}\ (23)$$

$$\mathrm{TOF}_{IFONFs-15} = \frac{3.12 \times 10^{15} \times \frac{H_2/_s}{cm^2}\ per\ \frac{mA}{cm^2} \times 0.34\ \frac{mA}{cm^2}}{0.410 \times 10^{18}\ sites\ cm^2} = 0.00259\ s^{-1}\ (24)$$

$$\mathrm{TOF}_{IFONFs-30} = \frac{3.12 \times 10^{15} \times \frac{H_2/_s}{cm^2}\ per\ \frac{mA}{cm^2} \times 0.32\ \frac{mA}{cm^2}}{0.410 \times 10^{18}\ sites\ cm^2} = 0.00244\ s^{-1}\ (25)$$

$$\mathrm{TOF}_{IFONFs-45} = \frac{3.12 \times 10^{15} \times \frac{H_2/_s}{cm^2}\ per\ \frac{mA}{cm^2} \times 36.4\ \frac{mA}{cm^2}}{0.410 \times 10^{18}\ sites\ cm^2} = 0.27700\ s^{-1}\ (26)$$

$$\mathrm{TOF}_{IFONFs-60} = \frac{3.12 \times 10^{15} \times \frac{H_2/_s}{cm^2}\ per\ \frac{mA}{cm^2} \times 12.6\ \frac{mA}{cm^2}}{0.410 \times 10^{18}\ sites\ cm^2} = 0.09588\ s^{-1}\ (27)$$

$$\mathrm{TOF}_{IFONFs-90} = \frac{3.12 \times 10^{15} \times \frac{H_2/_s}{cm^2}\ per\ \frac{mA}{cm^2} \times 10.2\ \frac{mA}{cm^2}}{0.410 \times 10^{18}\ sites\ cm^2} = 0.07762\ s^{-1}\ (28)$$

Since the difficulty in attributing the observed peaks to a given redox couple, the number of active sites should be proportional to the integrated charge over the CV curve (**Supplementary Fig. 30**). Assuming a one-electron process for both reduction and oxidation, the upper limit of active sites (n) for IFONFs-45 could be calculated according to the follow equation:

n = Q/2F (29)

where F and Q are the Faraday constant and the whole charge of CV curve, respectively.

$$n_{\mathrm{Pt/C}} = \frac{0.2776}{2*96485} = 1.44 * 10^{-6}\ (30)$$

$$n_{IFONFs-30} = \frac{0.0374}{2*96485} = 1.94 * 10^{-7}\ (31)$$

$$n_{IFONFs-45} = \frac{0.2096}{2*96485} = 1.09 * 10^{-6}\ (32)$$

$$n_{IFONFs-60} = \frac{0.0436}{2*96485} = 2.26 * 10^{-7}\ (33)$$

Supplementary Note 6: Calculation of TOF the number of active sites **for OER.**

The values of TOF for OER were calculated by assuming that every metal atom was involved in the catalysis (lower limits):

$$TOF = \frac{j}{4 \times F \times n}\ (34)$$

where j is the current density (A cm^{-2}), F is Faraday's constant (96485.3 C mol^{-1}) and n is moles of electrocatalysts (mol cm^{-2}). Accordingly, at an overpotential of 1.6 V, the OER current density for Fe-oxide, IFONFs-15, IFONFs-30, IFONFs-45, IFONFs-60, and IFONFs-90 were 3.7, 8.4, 30.4, 56.3, 45.9, and 9.1 mA cm^{-2}, respectively, and the corresponding TOF values were calculated to be

$$\mathrm{TOF_{Fe-oxide}} = \frac{3.7 \times mA\,cm^{-2}}{4 \times 96485.3\ C\,mol^{-1}\frac{19\,mg}{100\,mg} \times 0.2\,\frac{mg}{cm^2} \times \frac{1}{55.85}\frac{mmol}{mg}} = 0.0141\,s^{-1} \quad (35)$$

$$\mathrm{TOF_{IFONFs-15}} = \frac{8.4 \times mA\,cm^{-2}}{4 \times 96485.3\ C\,mol^{-1}\frac{19\,mg}{100\,mg} \times 0.2\,\frac{mg}{cm^2} \times \frac{1}{55.85}\frac{mmol}{mg}} = 0.0319\,s^{-1} \quad (36)$$

$$\mathrm{TOF_{IFONFs-30}} = \frac{30.4 \times mA\,cm^{-2}}{4 \times 96485.3\ C\,mol^{-1}\frac{19\,mg}{100\,mg} \times 0.2\,\frac{mg}{cm^2} \times \frac{1}{55.85}\frac{mmol}{mg}} = 0.1156\,s^{-1} \quad (37)$$

$$\mathrm{TOF_{IFONFs-45}} = \frac{56.3 \times mA\,cm^{-2}}{4 \times 96485.3\ C\,mol^{-1}\frac{19\,mg}{100\,mg} \times 0.2\,\frac{mg}{cm^2} \times \frac{1}{55.85}\frac{mmol}{mg}} = 0.2141\,s^{-1} \quad (38)$$

$$\mathrm{TOF_{IFONFs-60}} = \frac{45.9 \times mA\,cm^{-2}}{4 \times 96485.3\ C\,mol^{-1}\frac{19\,mg}{100\,mg} \times 0.2\,\frac{mg}{cm^2} \times \frac{1}{55.85}\frac{mmol}{mg}} = 0.1745\,s^{-1} \quad (39)$$

$$\mathrm{TOF_{IFONFs-90}} = \frac{9.1 \times mA\,cm^{-2}}{4 \times 96485.3\ C\,mol^{-1}\frac{19\,mg}{100\,mg} \times 0.2\,\frac{mg}{cm^2} \times \frac{1}{55.85}\frac{mmol}{mg}} = 0.0346\,s^{-1} \quad (40)$$

A linear plot between the oxidation currents for redox species and scan rates can be derived from cyclic voltammograms, and the corresponding slopes can be obtained from the linear plots **(Supplementary Fig. 36)**. The quantity of active species (m) is calculated based on the formula: slope = $n^2F^2m/4RT$, where n is the number of electrons transferred, which is denoted as 1 in order to achieve the upper limit in the concentration of active sites, F is the Faradic constant (96485 C mol^{-1}), m is the number of active species, and R and T are the ideal gas constant (8.314 J mol^{-1} K^{-1}) and absolute temperature (298 K), respectively. The results showed that the number of active sites for IFONFs-45 is 2.59×10^{-8} mol, much larger than that of IFONFs-30 (1.26×10^{-8} mol) and IFONFs-60 (1.50×10^{-8} mol).

$$m_{Fe-oxide} = \frac{0.00406*4*8.314*298}{96485*96485} = 4.31*10^{-9}\ \mathrm{mol} \quad (41)$$

$$m_{IFONFs-15} = \frac{0.00646*4*8.314*298}{96485*96485} = 6.88*10^{-9}\ \mathrm{mol} \quad (42)$$

$$m_{IFONFs-30} = \frac{0.01181*4*8.314*298}{96485*96485} = 1.26*10^{-8}\ \mathrm{mol} \quad (43)$$

$$m_{IFONFs-45} = \frac{0.02431*4*8.314*298}{96485*96485} = 2.59*10^{-8}\ \mathrm{mol} \quad (44)$$

$$m_{IFONFs-60} = \frac{0.0141*4*8.314*298}{96485*96485} = 1.50*10^{-8}\ \mathrm{mol} \quad (45)$$

$$m_{IFONFs-90} = \frac{0.00791*4*8.314*298}{96485*96485} = 8.42*10^{-9}\ \mathrm{mol} \quad (46)$$

Supplementary Note 7: Determination of Faradaic efficiency

The generated gas was confirmed by gas chromatography (GC) analysis and measured quantitatively using a calibrated pressure sensor to monitor the pressure change in the cathode compartment of an H-type electrolytic cell. The Faradic efficiency was calculated by comparing the amount of measured hydrogen generated by galvanostatic electrolysis with calculated hydrogen (assuming 100% FE). The rough agreement of both values (**Supplementary Fig. 41**) suggests nearly 100% FE for HER and OER in 1 M KOH. GC analysis was carried out on GC–2014C (Shimadzu Co.) with thermal conductivity detector and nitrogen carrier gas. Pressure data during electrolysis were recorded using a CEM DT-8890 Differential Air Pressure Gauge

Supplementary Note 8: Computational methods.

We performed spin-polarized Density Functional Theory (DFT) calculations with the LDA+U approach by using Vienna *Ab-initio* Simulation Package (VASP)[38]. Specifically, the projector augmented wave (PAW) method for potentials at the core region [39, 40] and the generalized gradient approximation (GGA) of Perdew–Burke–Ernzerhof (PBE) exchange-correlation functional were adopted[41, 42, 43]. A U = 5 eV was used to correct the correlation of Fe d-electrons[44]. A kinetic energy cut-off of 400 eV was used for the plane-wave expansion, and all atomic positions were fully relaxed with a Γ point until the final force on each atom was less than 0.01 eV/Å.
Atomic coordinates of FeF_2 (110) surface, with stoichiometric termination, and with O-Fe dimer adsorption. The corresponding configurations with H adsorption are also provided. The coordinates are listed in VASP CONTCAR format.

per woH

1.00000000000000

13.3670000000000009	0.0000000000000000	0.0000000000000000
0.0000000000000000	13.5719200000000004	0.0000000000000000
0.0000000000000000	0.0000000000000000	25.0000000000000000

Fe F

64 128

Selective dynamics

Direct

0.1917535075949751	0.9998284089954753	0.3286088393706087	T	T	T
0.1926441589518459	0.2498520220547422	0.4724982727070881	T	T	T
0.1934791218792171	0.4998207196054237	0.6027933237550930	T	T	T
0.1965432843015685	0.2497170521389195	0.7467951620810844	T	T	T
0.4417658825201237	0.9998268869989784	0.3286709164978888	T	T	T
0.4426133079209648	0.2498251750247560	0.4725340292450542	T	T	T
0.4434429743644385	0.4997984649001586	0.6027863569464238	T	T	T
0.4464532365224838	0.2497360407034168	0.7468114762756118	T	T	T
0.1916551893038395	0.4998934972040415	0.3286232146927167	T	T	T
0.1926726831092073	0.7498634434599148	0.4725157269468892	T	T	T
0.1935018540562344	0.9997984378891674	0.6027802840024162	T	T	T

0.1966181110966958	0.7497247612810011	0.7467833654007688	T	T	T
0.4416748379431611	0.4998462284074759	0.3286968109045353	T	T	T
0.4426827491486817	0.7498114689286656	0.4725442327940723	T	T	T
0.4434431365356682	0.9998148048744212	0.6027816883998574	T	T	T
0.4465461806536836	0.7497365084412548	0.7468201228371779	T	T	T
0.6917316610424499	0.9999609656852549	0.3286486140381756	T	T	T
0.6926178536063807	0.2498713240921431	0.4725480088078292	T	T	T
0.6934849000812521	0.4998062171105593	0.6027945574081275	T	T	T
0.6965447962836663	0.2497182873510695	0.7468117225990775	T	T	T
0.9417435018451926	0.9999608253620136	0.3287497929237206	T	T	T
0.9426570963261146	0.2498160301573074	0.4725760794416402	T	T	T
0.9434345038915498	0.4998200185038321	0.6027782823893942	T	T	T
0.9464506359172280	0.2497172595278103	0.7468101928977660	T	T	T
0.6916881607501849	0.4999194419962800	0.3286151040370221	T	T	T
0.6926408831345848	0.7498598528312620	0.4725181792628916	T	T	T
0.6934712016717242	0.9998274801287134	0.6028116304872940	T	T	T
0.6966275213017922	0.7497325336258153	0.7467986127554890	T	T	T
0.9416805528497363	0.4999665157021388	0.3286952833876105	T	T	T
0.9426415060195806	0.7498384237738193	0.4725403379284195	T	T	T
0.9434407787385318	0.9998044186982933	0.6027855746494624	T	T	T
0.9465362022477783	0.7497204426240118	0.7467956939702208	T	T	T
0.0669393832736233	0.2500191449369707	0.3375365098242720	T	T	T
0.0677964725031350	0.4997870004494260	0.4680447789537612	T	T	T
0.0679013062817909	0.2496678730446231	0.6071524934939286	T	T	T
0.0711234273903660	0.9998783935563189	0.7380768645366991	T	T	T
0.3169502972052269	0.2497417948274141	0.3374827329829518	T	T	T
0.3177728854238150	0.4997688623913119	0.4680487390088382	T	T	T

0.3179500168985119	0.2496818654757503	0.6071470000671167	T	T	T
0.3211243683388344	0.9997951627730368	0.7380889857116422	T	T	T
0.0669472802049558	0.7500228170271869	0.3375411888844361	T	T	T
0.0677542995227141	0.9997586569469072	0.4680531269281242	T	T	T
0.0678991681286884	0.7496701430220248	0.6071401513716870	T	T	T
0.0711332805573857	0.4998781751349582	0.7380806631951254	T	T	T
0.3170039908596254	0.7497972962088467	0.3375228355989918	T	T	T
0.3177744310547566	0.9997616540226778	0.4680241664074176	T	T	T
0.3179815261380611	0.7496746009212180	0.6071340336613222	T	T	T
0.3211338443160160	0.4998111358036295	0.7380955260747399	T	T	T
0.5669198527677058	0.2499804117382181	0.3375440136389701	T	T	T
0.5677839334782265	0.4997653082501614	0.4680493815348043	T	T	T
0.5679003737379558	0.2496738115529065	0.6071742859400869	T	T	T
0.5711291903062230	0.9998915617235429	0.7380996643545273	T	T	T
0.8169773361867843	0.2499764001267319	0.3375881941193852	T	T	T
0.8178009688710469	0.4997863803651175	0.4680446239697580	T	T	T
0.8179813382477097	0.2496808737885215	0.6071601428694299	T	T	T
0.8211200600912598	0.9997825063792565	0.7381112784017800	T	T	T
0.5669780906949002	0.7499683268585492	0.3375340921836553	T	T	T
0.5677686418931335	0.9998050892812512	0.4680469997667638	T	T	T
0.5679023726524500	0.7496828826757752	0.6071558342465748	T	T	T
0.5711437225824280	0.4998843473741462	0.7380909326391243	T	T	T
0.8169845225964122	0.7499330242409890	0.3375272606505391	T	T	T
0.8177319250488406	0.9998004683419959	0.4680765393748467	T	T	T
0.8179489865309852	0.7496802986549264	0.6071410776027480	T	T	T
0.8211284443997124	0.4997982783048419	0.7380994678510432	T	T	T
0.0676277583293859	0.9993546203107568	0.3872545976183356	T	T	T

0.0677612833816070	0.2499206822515370	0.5259337100300673	T	T	T
0.0686947869062736	0.0005967376726773	0.6566723897930223	T	T	T
0.1914677958753090	0.3410003233460753	0.3290872984131647	T	T	T
0.1927754375610591	0.0999935299071189	0.4693140308645663	T	T	T
0.1929567511301877	0.3499999135016587	0.6055964867505885	T	T	T
0.1983506319039581	0.0908362167254250	0.7474793359222593	T	T	T
0.0663140554427427	0.0001630705008053	0.2846209807133346	T	T	T
0.0674758970399953	0.2500445798771526	0.4188708152583013	T	T	T
0.0682373454760996	0.4996938527577232	0.5493484333224253	T	T	T
0.0671930904378840	0.2485293014396303	0.6879490002697231	T	T	T
0.1924768678492033	0.1586188672442415	0.3295260082753690	T	T	T
0.1927680901355508	0.3996985508567003	0.4697561694843372	T	T	T
0.1929728040209146	0.1496186446104357	0.6050252828197187	T	T	T
0.1937356054229532	0.4083513650493423	0.7455087516528395	T	T	T
0.0729254264000086	0.2507322642752313	0.7908081163654882	T	T	T
0.3177804061124329	0.9993291520444274	0.3872142248016874	T	T	T
0.3177308862655148	0.2499026826865233	0.5259216234549347	T	T	T
0.3187499008570380	0.0005695380340841	0.6566855598191845	T	T	T
0.4414840750792287	0.3409705248653396	0.3290506879222617	T	T	T
0.4427193353851859	0.0999778319354414	0.4693496701926529	T	T	T
0.4429682199203659	0.3499856442395971	0.6057328350212997	T	T	T
0.4483135717139418	0.0908519001374144	0.7474896348039497	T	T	T
0.3164400487716008	1.0000335975404400	0.2845357967376157	T	T	T
0.3175569881232617	0.2502230401595517	0.4188244381281387	T	T	T
0.3182480861464659	0.4996468537764230	0.5493604698954725	T	T	T
0.3173210537713232	0.2486542889614005	0.6879704674539062	T	T	T
0.4424094385179723	0.1585802024687236	0.3295619430401234	T	T	T

0.4427281561396595	0.3996740116619801	0.4699694116349039	T	T	T
0.4429443758032360	0.1496311375539466	0.6050122754084738	T	T	T
0.4437933938452192	0.4083697590996802	0.7455186223306765	T	T	T
0.3228908659223078	0.2508709838310484	0.7908682781556431	T	T	T
0.0679516574568566	0.4993713648080856	0.3872380199271537	T	T	T
0.0677273279669021	0.7499132931374247	0.5259214172375215	T	T	T
0.0686627026639820	0.5006713713781823	0.6566765670641073	T	T	T
0.1916385246356772	0.8410140830563483	0.3291864735198887	T	T	T
0.1927937382678032	0.6000176860494872	0.4693653539044920	T	T	T
0.1929779649998231	0.8499770105798868	0.6055549429784115	T	T	T
0.1984439738497653	0.5908381859222038	0.7474836467759701	T	T	T
0.0661554620757956	0.5002278672385710	0.2845781362167785	T	T	T
0.0675170324331336	0.7502227178818200	0.4188737217875176	T	T	T
0.0682540933348270	0.9996171985163791	0.5493497192796827	T	T	T
0.0672116431629692	0.7485240531159567	0.6879370821315538	T	T	T
0.1923394753513061	0.6586918295694991	0.3295530685676358	T	T	T
0.1927925810125524	0.8996998055890075	0.4697678978732557	T	T	T
0.1929845067769082	0.6496371196515446	0.6049972214931277	T	T	T
0.1938154841086185	0.9083488250281380	0.7455205637774981	T	T	T
0.0730393921194966	0.7507223036297918	0.7907958161555388	T	T	T
0.3179212200568615	0.4993908536080167	0.3872444423333184	T	T	T
0.3178037269017414	0.7499005963993821	0.5259180370330861	T	T	T
0.3187574594047151	0.5006487309366148	0.6566940783033506	T	T	T
0.4416297271960118	0.8410311681953072	0.3291771334084417	T	T	T
0.4427745041720852	0.5999476458841430	0.4694139429505366	T	T	T
0.4429896402944282	0.8500033560893254	0.6056614717537222	T	T	T
0.4484256745579187	0.5908530612793446	0.7475397778211329	T	T	T

0.3162778351520767	0.5004110093440020	0.2845903985664170	T	T	T
0.3176219262636087	0.7500163719760098	0.4188619945646858	T	T	T
0.3182686046545439	0.9997187316150321	0.5493402675711343	T	T	T
0.3173236625925244	0.7486349570500910	0.6879510673978133	T	T	T
0.4424421181675193	0.6586518886428802	0.3295158851206005	T	T	T
0.4427905713003590	0.8996862039590011	0.4699653015068563	T	T	T
0.4429817162557794	0.6496122328220370	0.6049552603821452	T	T	T
0.4438858312429836	0.9083857193887468	0.7455379142322571	T	T	T
0.3229934692258419	0.7508818688519874	0.7908662561983916	T	T	T
0.5678340426863137	0.9993969591012232	0.3872285844483481	T	T	T
0.5676708864137142	0.2499109244131953	0.5259473846942025	T	T	T
0.5686722404483620	0.0005575267698437	0.6566932105352457	T	T	T
0.6916215093971931	0.3411469918481433	0.3292806184019646	T	T	T
0.6927335855630905	0.1000318916738155	0.4693978716455005	T	T	T
0.6929773862497667	0.3499895522670254	0.6055930467058535	T	T	T
0.6983205269870022	0.0908290653485181	0.7475449343239804	T	T	T
0.5663216620842989	0.0002307661447936	0.2845722328060017	T	T	T
0.5674648162714661	0.2500929162857772	0.4188807580845390	T	T	T
0.5682384147594295	0.4996056548617134	0.5493504853209609	T	T	T
0.5673345551925207	0.2485442402066211	0.6879722659242048	T	T	T
0.6923214725581994	0.1588171050346226	0.3295323599288967	T	T	T
0.6927974691810370	0.3997100940903646	0.4697735226260951	T	T	T
0.6929719077317749	0.1496430925577186	0.6050449300437405	T	T	T
0.6937497559638561	0.4083619487711203	0.7455271435874348	T	T	T
0.5728852151282473	0.2507998898534207	0.7908139116383780	T	T	T
0.8178232190340858	0.9995364005010333	0.3872824992838882	T	T	T
0.8177459723870398	0.2499430416565546	0.5259508199600507	T	T	T

0.8187555259692515	0.0006738081565186	0.6567073148851740	T	T	T
0.9414401115019583	0.3411999136299322	0.3292003730473957	T	T	T
0.9427588354295587	0.0999594865500355	0.4694700985507810	T	T	T
0.9429992143169221	0.3500103696899060	0.6056775357116951	T	T	T
0.9483022797113331	0.0908077923528833	0.7475449789222469	T	T	T
0.8164083051028693	0.0005518696995715	0.2846253577390207	T	T	T
0.8175871084243399	0.2500162112981177	0.4189204140964652	T	T	T
0.8182456479951365	0.4996948324872780	0.5493537203773914	T	T	T
0.8174208927396669	0.2486084168885133	0.6879765388866346	T	T	T
0.9425152458675271	0.1588050792756338	0.3295612611287150	T	T	T
0.9427675007894131	0.3996860017588950	0.4699772466344718	T	T	T
0.9429729571903857	0.1496172891827927	0.6049463611189531	T	T	T
0.9438191882821914	0.4083596471689280	0.7455293228521198	T	T	T
0.8228857213717569	0.2508930261923951	0.7908696607306221	T	T	T
0.5677712480382390	0.4993384439108956	0.3872330982124340	T	T	T
0.5677410693620932	0.7499656300513114	0.5259367152113531	T	T	T
0.5686855029678246	0.5005971168709021	0.6566841736112401	T	T	T
0.6916222669880523	0.8411166096462759	0.3291912517165845	T	T	T
0.6928045754896406	0.5999899152442558	0.4693614870365656	T	T	T
0.6929607685192800	0.8500065747341522	0.6055927455137292	T	T	T
0.6984395597374060	0.5908524009214955	0.7475054593882464	T	T	T
0.5661911116515459	0.5000460776958231	0.2845932047017235	T	T	T
0.5675274671424815	0.7501014152674390	0.4188694570883697	T	T	T
0.5682360627673473	0.9997412604497878	0.5493547445926469	T	T	T
0.5671972468881219	0.7485409868754213	0.6879569330094736	T	T	T
0.6923707278735455	0.6587453199871390	0.3295060890284872	T	T	T
0.6927156037526481	0.8997107539910408	0.4697527116682546	T	T	T

0.6929572941202671	0.6496275471615750	0.6050238607601280	T	T	T
0.6938405879025332	0.9083663528136687	0.7455301963216506	T	T	T
0.5730429258221638	0.7507958962944057	0.7908144536162698	T	T	T
0.8179858992497830	0.4994706364281405	0.3872315079518212	T	T	T
0.8177285928829264	0.7498362940696583	0.5259210801997456	T	T	T
0.8187567408619942	0.5006150615231991	0.6566949365633457	T	T	T
0.9414960266418378	0.8411577542738149	0.3291544534111324	T	T	T
0.9427869283877661	0.5999867868202573	0.4693647820180850	T	T	T
0.9429637467462408	0.8499947586031656	0.6056835621879487	T	T	T
0.9483942154426700	0.5908409281871149	0.7475217669942207	T	T	T
0.8162843554313153	0.5005254491305974	0.2845641168712997	T	T	T
0.8175607812744962	0.7500858225072773	0.4188622041670704	T	T	T
0.8182268361605285	0.9996856405017883	0.5493847716917167	T	T	T
0.8172891366288426	0.7486548173210940	0.6879571284884933	T	T	T
0.9424701010685564	0.6587666669760283	0.3295410353004434	T	T	T
0.9426642097926021	0.8996844340850672	0.4700006546771913	T	T	T
0.9429371042565502	0.6496389285183793	0.6049548640088731	T	T	T
0.9439130487730724	0.9083506260138228	0.7454912773418328	T	T	T
0.8230251582724574	0.7508928967659457	0.7908652121336062	T	T	T

per wH

1.00000000000000

13.3670000000000009	0.0000000000000000	0.0000000000000000
0.0000000000000000	13.5719200000000004	0.0000000000000000
0.0000000000000000	0.0000000000000000	25.0000000000000000

Fe F H

64 128 1

Selective dynamics

Direct

0.1917951254792667	0.9998249521814799	0.3283341279839310	T	T	T
0.1926745915647252	0.2498831746710497	0.4725900044550574	T	T	T
0.1943300150641639	0.4998372710750746	0.6036434999311172	T	T	T
0.1965497903515102	0.2496829406366773	0.7469720808756088	T	T	T
0.4417822411761595	0.9998360562700570	0.3284074993741303	T	T	T
0.4426350285156927	0.2498604908420114	0.4726392144642588	T	T	T
0.4426490117790164	0.4998044935244382	0.6036629019934611	T	T	T
0.4463358739827160	0.2496402564949123	0.7470102702854554	T	T	T
0.1917025868995014	0.4998692627246545	0.3283436799310159	T	T	T
0.1926931029379870	0.7498358908534599	0.4726032947752831	T	T	T
0.1934971193971030	0.9997961259108543	0.6027042367449558	T	T	T
0.1966325734342110	0.7498542734783656	0.7469353284942358	T	T	T
0.4417318046495201	0.4998467024017007	0.3284370842399023	T	T	T
0.4426857028965414	0.7497782838864302	0.4726502681038466	T	T	T
0.4434195981737010	0.9998097679837293	0.6027120414339844	T	T	T
0.4464232635847649	0.7498552251549742	0.7470213962359279	T	T	T
0.6918047673325677	0.9999550883000163	0.3283608677965357	T	T	T
0.6926413172260879	0.2498993893541530	0.4726216104764108	T	T	T
0.6933842646911484	0.4998014971804983	0.6026323027341425	T	T	T
0.6965026967906975	0.2497253998611320	0.7469847547762227	T	T	T
0.9417799710482637	0.9999556591764215	0.3284758181011318	T	T	T
0.9426771286025224	0.2498409253283063	0.4726684706423913	T	T	T
0.9435066535418620	0.4998235984658696	0.6026256778732777	T	T	T
0.9463836298264715	0.2496988964941619	0.7470017553036130	T	T	T
0.6917208747438683	0.4999236859459329	0.3283535291994759	T	T	T
0.6926694178558396	0.7498350058017276	0.4725984084431707	T	T	T

0.6934645250088228	0.9998092965148061	0.6027384735437630	T	T	T
0.6965732424531494	0.7497547875696244	0.7469634868916405	T	T	T
0.9417309023923851	0.4999626410195396	0.3284375398692541	T	T	T
0.9426704178522901	0.7498191319140144	0.4726346817849594	T	T	T
0.9434174756583652	0.9998047665890070	0.6027212997956374	T	T	T
0.9464763451518605	0.7497384636817713	0.7469884356423556	T	T	T
0.0669379852262806	0.2500022112406367	0.3375343159708528	T	T	T
0.0678549283020517	0.4997828889125411	0.4680267986945388	T	T	T
0.0678754542740758	0.2497118958577240	0.6073309377456905	T	T	T
0.0710276368844770	0.9998873952291712	0.7379898914839111	T	T	T
0.3169487308440337	0.2497550089545598	0.3374852016711267	T	T	T
0.3178143698087304	0.4997762364940171	0.4679818559435690	T	T	T
0.3179667542967566	0.2497836293263171	0.6073771215053729	T	T	T
0.3210791684295964	0.9997649519759426	0.7380104481052592	T	T	T
0.0669421633088497	0.7500061780210913	0.3375367654976105	T	T	T
0.0677741489798801	0.9997695467708363	0.4679175313046169	T	T	T
0.0678690886999923	0.7496583033481388	0.6073213902926534	T	T	T
0.0710754764236402	0.4998015782450335	0.7377525536866344	T	T	T
0.3169836279839013	0.7497362817742822	0.3375128001915956	T	T	T
0.3177994590891279	0.9997743249733321	0.4678883003689398	T	T	T
0.3179810014149687	0.7496053885747516	0.6073673739632944	T	T	T
0.3210248010133541	0.4997822380613908	0.7417956845566600	T	T	T
0.5669279981889098	0.2499221804863383	0.3375392569482821	T	T	T
0.5677870879360982	0.4997715518249073	0.4680329612482124	T	T	T
0.5679224152336739	0.2497238820355584	0.6073531304521310	T	T	T
0.5711560307982017	0.9999294479523710	0.7380097220256796	T	T	T
0.8169740708421254	0.2500045786558183	0.3375724612665116	T	T	T

0.8178367231617035	0.4997977885026121	0.4679267992061982	T	T	T
0.8179737839356052	0.2497074947391823	0.6073615140593657	T	T	T
0.8210751090913428	0.9997970822106802	0.7380444377787250	T	T	T
0.5669667448352791	0.7500563642734285	0.3375341090123684	T	T	T
0.5677843230371719	0.9997929470932013	0.4679118621318493	T	T	T
0.5679192951508087	0.7496547255979659	0.6073393629887592	T	T	T
0.5710835566738182	0.4999622236559944	0.7377517297732599	T	T	T
0.8169763192098285	0.7499257799602932	0.3375112785570561	T	T	T
0.8177622354583830	0.9998090605996103	0.4679304419894627	T	T	T
0.8179521918407004	0.7496763328940539	0.6073430317357471	T	T	T
0.8210769302655707	0.4997994411840858	0.7380360264911758	T	T	T
0.0676896194151322	0.9993745142138419	0.3870685921370049	T	T	T
0.0677736081968836	0.2499128698862595	0.5260899779358307	T	T	T
0.0686869949531228	0.0005583143395720	0.6566005717613812	T	T	T
0.1915400956249761	0.3409865975379087	0.3289787247369206	T	T	T
0.1927943710837646	0.0999950498155152	0.4692824910582123	T	T	T
0.1930345047301969	0.3500553738873347	0.6057822012971983	T	T	T
0.1982532651806167	0.0908442232205281	0.7473060603626734	T	T	T
0.0663746491044173	0.0001539700779222	0.2843318720308767	T	T	T
0.0674941960630630	0.2500389989205044	0.4188890476991539	T	T	T
0.0685498944434761	0.4996889356488801	0.5493439768077756	T	T	T
0.0672060588364316	0.2486209149577283	0.6881814663921569	T	T	T
0.1923929182699383	0.1586082737572528	0.3293980095365747	T	T	T
0.1928432330677525	0.3996883599917050	0.4696887961669627	T	T	T
0.1929928535005877	0.1496388882714470	0.6052102211932804	T	T	T
0.1939312546762333	0.4083365622000154	0.7448061558636271	T	T	T
0.0730497296567486	0.2507346738112543	0.7909762843360273	T	T	T

0.3178386497942398	0.9993523555629016	0.3870196374972600	T	T	T
0.3177713209660608	0.2499101897639741	0.5261163390336061	T	T	T
0.3187341780962293	0.0004947836490340	0.6566281616370346	T	T	T
0.4416054107226995	0.3409564601396709	0.3289595074033241	T	T	T
0.4427484117546385	0.0999708926610393	0.4693637614367189	T	T	T
0.4429159323893046	0.3500371490303615	0.6059247898169553	T	T	T
0.4482783488071908	0.0908626401059250	0.7473339631802084	T	T	T
0.3164860510692504	0.0000410031937061	0.2842602271940674	T	T	T
0.3176003479957034	0.2501756391229353	0.4188449713496049	T	T	T
0.3182424563590380	0.4996522351902173	0.5496066810020186	T	T	T
0.3173868856418902	0.2484878072489283	0.6882669127916486	T	T	T
0.4423125556648946	0.1585732193483632	0.3294336809610047	T	T	T
0.4427291370987029	0.3996700994874913	0.4699605051662658	T	T	T
0.4429301512545425	0.1496503955666328	0.6052238084809674	T	T	T
0.4434235037312957	0.4085048604146440	0.7446719410707223	T	T	T
0.3227552517432259	0.2506241866502724	0.7911246166817746	T	T	T
0.0679501390108657	0.4993858599815451	0.3870457267899652	T	T	T
0.0677463879781622	0.7499528191402030	0.5260755338569291	T	T	T
0.0685118341050033	0.5006310714999953	0.6565470866336421	T	T	T
0.1916732240983011	0.8409836023743243	0.3290491275093366	T	T	T
0.1928503363223013	0.6000333980997922	0.4693166423138639	T	T	T
0.1930012402322166	0.8499660411144200	0.6057659411147045	T	T	T
0.1985158226035757	0.5909699438433317	0.7462382837440253	T	T	T
0.0662340056552791	0.5002027964607011	0.2842971851463784	T	T	T
0.0675287466140538	0.7501917524516970	0.4188900611842807	T	T	T
0.0682452323873683	0.9996398610516986	0.5492545127278265	T	T	T
0.0671802512502329	0.7486995845334381	0.6881666096830248	T	T	T

0.1922845481405155	0.6586489254147092	0.3294149173184865	T	T	T
0.1928085374957537	0.8997164716854076	0.4697077859318963	T	T	T
0.1930384770625020	0.6495738873619806	0.6051641894633008	T	T	T
0.1937993505686756	0.9083606137290383	0.7455956262973581	T	T	T
0.0732114721711408	0.7504929779642964	0.7909631555505328	T	T	T
0.3179478668782176	0.4993749352419284	0.3870181519429346	T	T	T
0.3178139512596957	0.7499185363212204	0.5261098385858863	T	T	T
0.3188526143521026	0.5005265413753276	0.6592763326084712	T	T	T
0.4417247153226195	0.8410212044008037	0.3290716443686767	T	T	T
0.4427555529084248	0.5999637149018126	0.4694340982732041	T	T	T
0.4429692054786286	0.8499866942098437	0.6058950887985869	T	T	T
0.4483311377255734	0.5910166900660059	0.7464315071815218	T	T	T
0.3163484709627026	0.5003294133856300	0.2842958432337120	T	T	T
0.3176492638213930	0.7500461760884208	0.4188735424610199	T	T	T
0.3182637881170701	0.9997169160469220	0.5492356620796298	T	T	T
0.3173082181107618	0.7490051930563537	0.6882518160808686	T	T	T
0.4423026314359431	0.6586394160113344	0.3293908697533856	T	T	T
0.4427978345421458	0.8996932856806311	0.4699670474405263	T	T	T
0.4428974468012795	0.6495678302718277	0.6051386449994582	T	T	T
0.4438186108698381	0.9084070845009001	0.7456584629041281	T	T	T
0.3228367860218387	0.7508801566772326	0.7911343617274170	T	T	T
0.5678285855607980	0.9994039346191527	0.3870478127263535	T	T	T
0.5677170367121936	0.2499059686203296	0.5260992744567706	T	T	T
0.5686342453802424	0.0004777029921691	0.6566220912321930	T	T	T
0.6916858754409918	0.3411314027148077	0.3291284455124491	T	T	T
0.6927519439397484	0.1000381214771154	0.4693512880967089	T	T	T
0.6930055019940314	0.3499395628658766	0.6058025612243176	T	T	T

0.6983020935926342	0.0909002393799945	0.7473781432302858	T	T	T
0.5663817514189612	0.0001947385864667	0.2842940582484714	T	T	T
0.5674834554004559	0.2501313375089966	0.4188957073003144	T	T	T
0.5679066522655415	0.4996246293358453	0.5493487951193546	T	T	T
0.5673721106971021	0.2486329937043327	0.6882071348441530	T	T	T
0.6922565483850612	0.1588073182216348	0.3294017188650002	T	T	T
0.6928479353978896	0.3996930968074408	0.4697010135610665	T	T	T
0.6929592207472965	0.1496283888956996	0.6052309013083859	T	T	T
0.6937063459105417	0.4084065672271113	0.7456420380444445	T	T	T
0.5725769688748540	0.2508097466164654	0.7909834279335329	T	T	T
0.8178335167645150	0.9995411356553972	0.3870826132438329	T	T	T
0.8177712576173026	0.2499327797211071	0.5260951525717186	T	T	T
0.8187305992123828	0.0005653902223708	0.6566434455252635	T	T	T
0.9415000919257810	0.3411975239427188	0.3290940470935801	T	T	T
0.9427648688880786	0.0999681074043611	0.4694834094137921	T	T	T
0.9429901798605557	0.3499666061174035	0.6059151613135829	T	T	T
0.9482392758361227	0.0908382019850080	0.7474031703976344	T	T	T
0.8164826910267778	0.0005088163724579	0.2843263864263946	T	T	T
0.8176208716242741	0.2500017335128817	0.4189300061530757	T	T	T
0.8182430445904967	0.4996879386309117	0.5492480830481684	T	T	T
0.8174674743763086	0.2487089987850107	0.6882038902433724	T	T	T
0.9424542391970745	0.1588026827752097	0.3294182468614767	T	T	T
0.9427846553573155	0.3996671126947788	0.4699754028285447	T	T	T
0.9429725616200187	0.1496191239909538	0.6051700932057991	T	T	T
0.9438860795191182	0.4083905497388659	0.7456516938889048	T	T	T
0.8227890825948271	0.2508230290077400	0.7910684483762861	T	T	T
0.5678329514224879	0.4993590778915200	0.3870484876644282	T	T	T

0.5677763502349288	0.7499782652743509	0.5260895784670550	T	T	T
0.5688671672870245	0.5005363882033071	0.6565377270750485	T	T	T
0.6917776421583177	0.8411604906360091	0.3290739241932333	T	T	T
0.6928278139038471	0.6000205995913305	0.4693027634278727	T	T	T
0.6929743791359770	0.8500057592972424	0.6057940077241761	T	T	T
0.6982993989390469	0.5908683351145054	0.7473699845038978	T	T	T
0.5662491602357321	0.5000619197864240	0.2843094578395503	T	T	T
0.5675289514117964	0.7499823550363047	0.4188874193433755	T	T	T
0.5682236983834902	0.9997233821381204	0.5492527530309217	T	T	T
0.5672849936009524	0.7487092235696894	0.6881853177265618	T	T	T
0.6921858344539652	0.6587979234047338	0.3293533829825606	T	T	T
0.6927735901669666	0.8997053650103733	0.4697037893612631	T	T	T
0.6929755073200822	0.6496677549427973	0.6052118911071471	T	T	T
0.6937305730863366	0.9084282492212525	0.7456523975364858	T	T	T
0.5727651124246496	0.7506125377335129	0.7909981128862921	T	T	T
0.8180073745678729	0.4994962410794942	0.3870427094188518	T	T	T
0.8177762295650756	0.7498934585148590	0.5260666073612060	T	T	T
0.8187427788824259	0.5005285404703239	0.6566343322954334	T	T	T
0.9415661491418640	0.8411578690878129	0.3290560306956395	T	T	T
0.9428006937838393	0.6000052715737089	0.4693754955630892	T	T	T
0.9429733934501943	0.8500057269268511	0.6059168045322816	T	T	T
0.9483367793869527	0.5908316850381100	0.7473989422037998	T	T	T
0.8163382474535806	0.5004877972006572	0.2842885587225244	T	T	T
0.8176142485885882	0.7500682808091447	0.4188784645791170	T	T	T
0.8182175547794783	0.9996791191539316	0.5492756302077765	T	T	T
0.8173554891814481	0.7487659964121376	0.6881870008219343	T	T	T
0.9424044427114610	0.6587752470778132	0.3294033783618361	T	T	T

0.9427062234764571	0.8996980569885102	0.4700116720551216	T	T	T
0.9429322100248643	0.6496869310243768	0.6051790263390608	T	T	T
0.9439290695705211	0.9083935319371346	0.7456128455544944	T	T	T
0.8229105343576950	0.7507895775069755	0.7910498662251338	T	T	T
0.3210917893623301	0.4994414188424790	0.8075991399379868	T	T	T

ad-FeO woH

1.00000000000000

13.3670000000000009	0.0000000000000000	0.0000000000000000
0.0000000000000000	13.5719200000000004	0.0000000000000000
0.0000000000000000	0.0000000000000000	25.0000000000000000

Fe	F	O
65	128	1

Selective dynamics

Direct

0.1918044147121147	0.9999348970701737	0.3275042114322455	T	T	T
0.1926790934719207	0.2498735616695843	0.4729658566072150	T	T	T
0.1943931228511732	0.4998660839398307	0.6050775872202877	T	T	T
0.1954639736497388	0.2509606632995597	0.7478800605349279	T	T	T
0.4418053872323448	0.9999510358887891	0.3273335791590290	T	T	T
0.4427407541441320	0.2499148537881269	0.4729057350784288	T	T	T
0.4440058873314512	0.4998371264677146	0.6088952174134132	T	T	T
0.4461296781828308	0.2475052877055610	0.7444800039221869	T	T	T
0.1919134723947347	0.4998620154666334	0.3285869746396574	T	T	T
0.1924413293492314	0.7499213216813103	0.4722169775136846	T	T	T
0.1930660661171057	0.0000077145418966	0.6022348154889552	T	T	T
0.1949307112899515	0.7464942421413501	0.7477389309495984	T	T	T
0.4418659107992711	0.4998481434898999	0.3294973412497448	T	T	T

0.4428939684511540	0.7497388200856262	0.4712208420247015	T	T	T
0.4433732332780011	0.9997340040348480	0.6022916849802397	T	T	T
0.4448623524094148	0.7643242854017209	0.7280728533684532	T	T	T
0.6918593711091049	0.9999705731313607	0.3275066659598562	T	T	T
0.6928654264372852	0.2499340663259692	0.4729843522504074	T	T	T
0.6932694754425689	0.4995757190677307	0.6043972265017558	T	T	T
0.6966877552799409	0.2509451318106835	0.7479706098936761	T	T	T
0.9418174530278112	0.9999623349901552	0.3274838668834991	T	T	T
0.9428536786112556	0.2499004692155445	0.4730414090173926	T	T	T
0.9437060348203368	0.4997927751896968	0.6031032362415344	T	T	T
0.9460983615046530	0.2506418738347593	0.7476697288504170	T	T	T
0.6917813957206197	0.4998761841348726	0.3285954000864262	T	T	T
0.6933085819519472	0.7498269615092998	0.4722577110476768	T	T	T
0.6938679962058284	-0.0000214376392692	0.6021166471705175	T	T	T
0.6951773105719857	0.7467452773039863	0.7476621511539380	T	T	T
0.9420171261913013	0.4998826921953031	0.3278833078334452	T	T	T
0.9429950354778537	0.7497070987587066	0.4728626615429631	T	T	T
0.9434864551841255	0.9999884604426139	0.6027531707857250	T	T	T
0.9449220086444583	0.7481423366363381	0.7477051950155194	T	T	T
0.0670410275803757	0.2499309377088794	0.3376725088528305	T	T	T
0.0683685912258975	0.4997639188982613	0.4682425614234380	T	T	T
0.0679660470775999	0.2500170228514068	0.6080716845327996	T	T	T
0.0703598813035919	0.9981504315517189	0.7383838572392574	T	T	T
0.3170454317012767	0.2499322166193368	0.3377060426616833	T	T	T
0.3182680877360576	0.4997880578477176	0.4696404812973760	T	T	T
0.3177869269814759	0.2496461916260086	0.6076727124695125	T	T	T
0.3185287119330211	0.0050335553358544	0.7378496454856839	T	T	T

0.0669658444453592	0.7499931531342603	0.3373296574716142	T	T	T
0.0678135303881989	0.9998241317649161	0.4673216879581681	T	T	T
0.0676669837059250	0.7498576727242049	0.6079033961327347	T	T	T
0.0729038658792778	0.5000062657830034	0.7389154503483737	T	T	T
0.3169831802254663	0.7498315908013913	0.3368672961152553	T	T	T
0.3179252018379826	0.9998573615994479	0.4671918883815496	T	T	T
0.3164005295821622	0.7498207882509486	0.6056049897288954	T	T	T
0.3333801517620274	0.4915417900160237	0.7443440516514518	T	T	T
0.5670407275983512	0.2499983309338681	0.3377226135848587	T	T	T
0.5676328041648221	0.4997692760981590	0.4693882382279750	T	T	T
0.5682834597964577	0.2494128739612030	0.6077097194219361	T	T	T
0.5736952546498252	0.0045534275027261	0.7376804120223232	T	T	T
0.8170714537284868	0.2499501609849770	0.3376994696367778	T	T	T
0.8180328564307490	0.4998189259329434	0.4680759509492569	T	T	T
0.8181886747399778	0.2498273989845151	0.6080309731118363	T	T	T
0.8214712401575067	0.9984569489831390	0.7383194425122180	T	T	T
0.5671096091212103	0.7499243437353008	0.3368761585982424	T	T	T
0.5679617600232332	0.9998513357249952	0.4671868176422943	T	T	T
0.5708882427842459	0.7498884321456056	0.6057443743566872	T	T	T
0.5551887551963316	0.4923404782191704	0.7438841879719544	T	T	T
0.8171217727011912	0.7498712031657029	0.3373747897975397	T	T	T
0.8180522482563313	0.9998398755574963	0.4673074780715014	T	T	T
0.8192805190740973	0.7498085462229946	0.6077905090897402	T	T	T
0.8162195749293945	0.4994160442106523	0.7387331370751793	T	T	T
0.4481950336355713	0.6393282329861248	0.8140134381644479	T	T	T
0.0679030174182824	0.9995053941590545	0.3863009482695984	T	T	T
0.0678138480190671	0.2498636606067420	0.5265529385090155	T	T	T

0.0691122616754808	0.9986078396146261	0.6569420022500014	T	T	T
0.1916012423172550	0.3409310307465589	0.3289542488021320	T	T	T
0.1929701165201047	0.0998404044681665	0.4691651981286845	T	T	T
0.1931976850985566	0.3498398270945212	0.6066291050554580	T	T	T
0.1936308810876965	0.0903142469659901	0.7481236736650985	T	T	T
0.0664473495185207	0.0000999546279541	0.2832734011434976	T	T	T
0.0677031435473781	0.2499256243234842	0.4190807922658384	T	T	T
0.0696242381573817	0.4999968169707158	0.5497777891129921	T	T	T
0.0663080530851902	0.2492333609104369	0.6888121929181448	T	T	T
0.1924960352758374	0.1586015809407030	0.3291197190267744	T	T	T
0.1931471243550758	0.3997767756265594	0.4700146686651661	T	T	T
0.1921521022051595	0.1489688443731375	0.6047382010143836	T	T	T
0.1949086630696296	0.4076825439268558	0.7471362963349893	T	T	T
0.0720401675787637	0.2498217772107710	0.7921520386191162	T	T	T
0.3180455012365368	0.9995450334414905	0.3862072287721619	T	T	T
0.3178427631240689	0.2497295672378967	0.5263115113249168	T	T	T
0.3189072834655518	0.9990139598186069	0.6567194329689079	T	T	T
0.4416499387575261	0.3409724800969561	0.3292359709261136	T	T	T
0.4429037371726286	0.0998138312353408	0.4690765311222526	T	T	T
0.4431151187329470	0.3494728221625591	0.6054216617071165	T	T	T
0.4476979133940779	0.0873845100642689	0.7496804259197721	T	T	T
0.3163853655486628	0.0000500214712088	0.2831840530315032	T	T	T
0.3175535386160137	0.2496975586191832	0.4190749834199818	T	T	T
0.3200602927048752	0.5010288634070791	0.5516202709690982	T	T	T
0.3122913944382332	0.2386395836959456	0.6877736596746806	T	T	T
0.4425011800989044	0.1586165556491218	0.3290498152293885	T	T	T
0.4427932341673886	0.3996451257924741	0.4697845389937313	T	T	T

0.4428709795817367	0.1488295500866046	0.6053847673705386	T	T	T
0.4453370550068332	0.3905406231755291	0.7192766434210391	T	T	T
0.3241256838951909	0.2482890353540277	0.7902084720197095	T	T	T
0.0680586620275368	0.4995240883018182	0.3868821376233492	T	T	T
0.0682312111985253	0.7501226687082252	0.5263517611702266	T	T	T
0.0679295951562079	0.5012728803427545	0.6573226442781910	T	T	T
0.1915730724846236	0.8411469966764840	0.3285969883682798	T	T	T
0.1932771353622324	0.6002148842983659	0.4695668274924144	T	T	T
0.1925313165534378	0.8503446749745441	0.6049072738112553	T	T	T
0.1975956890170917	0.5921535131867111	0.7495846238949379	T	T	T
0.0667558639062871	0.5001759934919616	0.2837923801655630	T	T	T
0.0674660173875944	0.7500857666721990	0.4187472135539064	T	T	T
0.0678407514288192	0.9994929559744560	0.5488351384235443	T	T	T
0.0663498954172623	0.7498362725294875	0.6890224190786434	T	T	T
0.1923113185866422	0.6588298791751686	0.3289525380403989	T	T	T
0.1929363119082524	0.8997400996316302	0.4691053647740279	T	T	T
0.1932130284767897	0.6506860363054725	0.6064279526178734	T	T	T
0.1975765521589971	0.9069659027173651	0.7481515239777246	T	T	T
0.0731563698561030	0.7521963774285073	0.7924228874663005	T	T	T
0.3180233029180309	0.4996120961609127	0.3880168458362084	T	T	T
0.3188869727549069	0.7501500255560143	0.5251452023922807	T	T	T
0.3145462033792799	0.5029344487179873	0.6612807311675658	T	T	T
0.4416273103443942	0.8410989403708160	0.3282298660366744	T	T	T
0.4428726824470909	0.6001956145031845	0.4692054954139128	T	T	T
0.4436359538616918	0.8489395465087431	0.6082921015973368	T	T	T
0.4514134450916845	0.6208686456313278	0.7198436252705763	T	T	T
0.3167750627625242	0.5002511577147906	0.2848184137163979	T	T	T

0.3172740641817884	0.7503857156409088	0.4182039863898878	T	T	T
0.3186083276043876	0.9984529856408947	0.5488735441550026	T	T	T
0.3061349185766287	0.7609975251257136	0.6876627660778001	T	T	T
0.4425090263354260	0.6588282174452067	0.3290235929921385	T	T	T
0.4429420300635817	0.8996551876490217	0.4691533750176175	T	T	T
0.4437058444355232	0.6521273002317509	0.6068849461186518	T	T	T
0.4431183429613939	0.9076976134937623	0.7447827717348620	T	T	T
0.3370181350857896	0.7383297160649311	0.7935931210939621	T	T	T
0.5678741408182367	0.9995250001640299	0.3862113050895847	T	T	T
0.5677707431371658	0.2496284193377765	0.5263200828969243	T	T	T
0.5682933972682194	0.9989399356057804	0.6566808050613032	T	T	T
0.6916839860941716	0.3410170289503173	0.3290409445892716	T	T	T
0.6928734366951114	0.0998679036954403	0.4691693239799840	T	T	T
0.6931431488916936	0.3493621990067426	0.6060578451810175	T	T	T
0.7016603184342414	0.0900704017083119	0.7482130153906835	T	T	T
0.5665308371680295	0.0000261989508040	0.2832141159008302	T	T	T
0.5677045053232852	0.2496994296856826	0.4190943675695305	T	T	T
0.5665848786086415	0.5009268543187331	0.5511745112355989	T	T	T
0.5723286918925019	0.2361118826430464	0.6878084423065315	T	T	T
0.6924923606988043	0.1587006875842046	0.3291179833628201	T	T	T
0.6927278409368081	0.3998047875138709	0.4698682349835429	T	T	T
0.6940084299283584	0.1487983360833635	0.6047022628954702	T	T	T
0.6935534367844017	0.4075834241138712	0.7468533425659628	T	T	T
0.5711949130575529	0.2479079324383020	0.7904705846952622	T	T	T
0.8179542392451475	0.9995456084365398	0.3862886589716896	T	T	T
0.8181073842685068	0.2497136170852049	0.5264824219483646	T	T	T
0.8181769204041485	0.9985253192461886	0.6569228010796317	T	T	T

0.9416182405704520	0.3410411996107999	0.3288787101922335	T	T	T
0.9429391669509138	0.0999582049424829	0.4690603738858200	T	T	T
0.9430375701893166	0.3500134614641296	0.6061332410648297	T	T	T
0.9479492445589921	0.0900182280373385	0.7470795439266177	T	T	T
0.8164652298924377	0.0003184535202701	0.2832578012233538	T	T	T
0.8177379607256243	0.2499637870268647	0.4190978696932508	T	T	T
0.8178498561616255	0.5001212832669673	0.5494407779701285	T	T	T
0.8184051184541957	0.2490279808837783	0.6888251237089881	T	T	T
0.9425509826877287	0.1586650671529594	0.3291035640166535	T	T	T
0.9432878882177889	0.3997298933469368	0.4697514789634329	T	T	T
0.9431420748761647	0.1492981873468816	0.6060094403089153	T	T	T
0.9442011846066253	0.4096350196459404	0.7461202658026550	T	T	T
0.8228218912727828	0.2503871436497722	0.7921261196920977	T	T	T
0.5682176972820157	0.4995993083935983	0.3878413800114531	T	T	T
0.5669779188311808	0.7501011449640664	0.5251753064091851	T	T	T
0.5745393468915833	0.5012684261483745	0.6602354057194254	T	T	T
0.6918239543760855	0.8411373282523089	0.3286199521404285	T	T	T
0.6927865548972209	0.6001321763439553	0.4693944577305975	T	T	T
0.6945165813783688	0.8504393920117381	0.6044425870010686	T	T	T
0.6921810838169321	0.5920210925618260	0.7499824162290888	T	T	T
0.5660277394446782	0.5001525023988631	0.2847432002237117	T	T	T
0.5682357358885871	0.7503546089103349	0.4182118117999280	T	T	T
0.5678320927831529	0.9985429284315971	0.5488479605929921	T	T	T
0.5838219734119645	0.7638184070841952	0.6878582343363803	T	T	T
0.6925579254636842	0.6588041921417154	0.3289800885162713	T	T	T
0.6929489467443861	0.8996897940384566	0.4690898843809315	T	T	T
0.6938112511917033	0.6506357054698071	0.6059797050213581	T	T	T

0.6896681345895823	0.9067041697137022	0.7489570734541965	T	T	T
0.5548399563854518	0.7402989239803944	0.7953618726420508	T	T	T
0.8182330633680079	0.4996077073824713	0.3869716097004334	T	T	T
0.8179856203977381	0.7499775002359904	0.5262406742809698	T	T	T
0.8194400220595269	0.5014967486356555	0.6570874216428361	T	T	T
0.9416226190810097	0.8411738859907469	0.3286127103481750	T	T	T
0.9435667271936048	0.5999427077561241	0.4693406360234390	T	T	T
0.9434889606950881	0.8503507137825971	0.6062160490566706	T	T	T
0.9452042826867182	0.5913410909120829	0.7478541342820296	T	T	T
0.8162699865278309	0.5002749520716647	0.2840308974087530	T	T	T
0.8181837163570289	0.7498684618282444	0.4187822314469213	T	T	T
0.8188918829010040	0.9996005791221919	0.5488116311138125	T	T	T
0.8212497493154394	0.7497748468599214	0.6888159163154812	T	T	T
0.9425146165190933	0.6587533994114794	0.3290169677833099	T	T	T
0.9429039070123751	0.8995960531753993	0.4693238373845993	T	T	T
0.9435269783836430	0.6498904578674437	0.6054029253384765	T	T	T
0.9434674934736645	0.9070001483226915	0.7470693530647633	T	T	T
0.8174813009239675	0.7509987123583798	0.7923081135375081	T	T	T
0.4425816565163402	0.5058946232659222	0.8009953207076076	T	T	T

ad-FeO wH

1.00000000000000

13.3670000000000009	0.0000000000000000	0.0000000000000000
0.0000000000000000	13.5719200000000004	0.0000000000000000
0.0000000000000000	0.0000000000000000	25.0000000000000000

Fe	F	O	H
65	128	1	1

Selective dynamics

Direct

0.1935618921876849	0.9999998876280370	0.3268344764099149	T	T	T
0.1933064537463780	0.2499103472351525	0.4732825317968535	T	T	T
0.1943779818067293	0.4998042147747645	0.6048390192057976	T	T	T
0.1996106707814301	0.2520729273421254	0.7483737053093060	T	T	T
0.4436186983108943	0.9998839765123622	0.3266290575658959	T	T	T
0.4432396142535295	0.2499225748811911	0.4730074764037518	T	T	T
0.4436235053265735	0.5003038518809462	0.6064462789267967	T	T	T
0.4497024152834659	0.2513672557864232	0.7457789897844188	T	T	T
0.1914584428196163	0.4998163509326400	0.3288357805471260	T	T	T
0.1928049141853057	0.7499977715813684	0.4720615212038768	T	T	T
0.1933827545237575	0.0002103959431007	0.6017567365092423	T	T	T
0.1919771538027257	0.7504480358294077	0.7481594671925753	T	T	T
0.4413354003220340	0.4997282325610963	0.3291926857586625	T	T	T
0.4435515665917009	0.7499176431790585	0.4714886143260708	T	T	T
0.4437541536137228	0.9999901625323735	0.6018901511270135	T	T	T
0.4385866175208177	0.7542159444301132	0.7365845267749882	T	T	T
0.6936275084161372	0.9999888291880840	0.3269291082152812	T	T	T
0.6935181164474449	0.2500259710061570	0.4729704483230041	T	T	T
0.6947107235876395	0.4998896318196621	0.6022008692654391	T	T	T
0.7002992948523756	0.2501323876264316	0.7482090848852748	T	T	T
0.9436436520163123	0.0000785854069426	0.3269720374876003	T	T	T
0.9435437690921166	0.2500300219156493	0.4731332982845712	T	T	T
0.9446258197238606	0.4998847783227377	0.6023274211074557	T	T	T
0.9500219102613038	0.2497419963249468	0.7483688348241295	T	T	T
0.6913428160047688	0.4997718821673537	0.3278444981332448	T	T	T
0.6933715406243246	0.7499136111953140	0.4723018568829617	T	T	T

0.6940130442987321	1.0000468695488820	0.6019532128024305	T	T	T
0.6938906894147460	0.7482618630186878	0.7468877827889631	T	T	T
0.9413752399836348	0.4998646201831746	0.3274990102607163	T	T	T
0.9431932912584097	0.7498749731796737	0.4725024836578725	T	T	T
0.9437356060817452	1.0000135029427528	0.6025468153376733	T	T	T
0.9422245869627747	0.7501168529927003	0.7478059894259548	T	T	T
0.0675400815872965	0.2499398779954157	0.3378102728766358	T	T	T
0.0687568789798950	0.4999714901391504	0.4681189675249482	T	T	T
0.0686011675078874	0.2499204834577347	0.6084076353815612	T	T	T
0.0707863706700364	0.0010600874463009	0.7379319081814675	T	T	T
0.3175651814579555	0.2498238623490494	0.3378559174254793	T	T	T
0.3185484419651924	0.4999323617268976	0.4693814978603156	T	T	T
0.3186078031365101	0.2498366261504250	0.6081276689418664	T	T	T
0.3199280097825026	0.0012740935487997	0.7372503532036817	T	T	T
0.0675534935896262	0.7499162037788559	0.3369065691877934	T	T	T
0.0681490070952340	1.0000036300368393	0.4668455976296199	T	T	T
0.0683305006158348	0.7499720562213018	0.6077867983519623	T	T	T
0.0724872298926814	0.5000938018623852	0.7383865489115911	T	T	T
0.3176509761662652	0.7499502904773867	0.3366367604056467	T	T	T
0.3182954765218022	0.9999050799356213	0.4666330003379748	T	T	T
0.3169615778335474	0.7506925517722567	0.6056536656292458	T	T	T
0.3234913749856250	0.5014541578917269	0.7435429310874856	T	T	T
0.5675112513625871	0.2497927647844226	0.3377412608649557	T	T	T
0.5680644838330159	0.4999052214575437	0.4684350769559482	T	T	T
0.5690045292617241	0.2499091074243641	0.6076451582331034	T	T	T
0.5712592918050591	0.0032075529213395	0.7372734361691904	T	T	T
0.8176058930382819	0.2499986927628257	0.3377170689734471	T	T	T

0.8184760717884010	0.4999408091998924	0.4672743775153370	T	T	T
0.8188723341378109	0.2498838947730855	0.6081009484199908	T	T	T
0.8205290614678712	0.9986094921373863	0.7380820471926568	T	T	T
0.5677188833295409	0.7497341572538313	0.3366902503651838	T	T	T
0.5683668276406787	0.9999779127584495	0.4666955468284897	T	T	T
0.5696918676104739	0.7502249670814172	0.6071201740385349	T	T	T
0.5737149592599166	0.4994276311822126	0.7380292290828453	T	T	T
0.8177365355145267	0.7500860743925917	0.3369613922292429	T	T	T
0.8183877821181388	0.9999654122343524	0.4669097066562353	T	T	T
0.8195619425852837	0.7502396580770181	0.6076462544375073	T	T	T
0.8236521604436029	0.4988844346663966	0.7370682192018341	T	T	T
0.4777838699161492	0.6146122250526977	0.8221709095593013	T	T	T
0.0686064004556665	0.9998598974253110	0.3857453939724617	T	T	T
0.0683884254570674	0.2495395077241291	0.5268475171785939	T	T	T
0.0692705913901760	0.9989444160803914	0.6564326021797025	T	T	T
0.1914600958519075	0.3410132908430490	0.3294166651851717	T	T	T
0.1933101872519057	0.1000053744895657	0.4689094375979941	T	T	T
0.1938313062968973	0.3497877201718658	0.6067996275839936	T	T	T
0.1995884909418076	0.0925680754553055	0.7467631121876448	T	T	T
0.0685500046447819	0.0005552926583676	0.2826499461550975	T	T	T
0.0684072155699889	0.2503257536685151	0.4192525617872360	T	T	T
0.0699552095653649	0.5001932438016777	0.5494293742132930	T	T	T
0.0679613824113800	0.2488976008698039	0.6891673230889228	T	T	T
0.1937257479329166	0.1587131997188615	0.3289107991374573	T	T	T
0.1937784043759868	0.3998013910319063	0.4709249372146663	T	T	T
0.1934026761973632	0.1495157847712477	0.6054582335830255	T	T	T
0.1949607344276172	0.4076654945777800	0.7464223770179838	T	T	T

0.0766083427792201	0.2474696918526656	0.7927433219061579	T	T	T
0.3186791048246521	0.9997466405809059	0.3855998656798250	T	T	T
0.3185882300269741	0.2499119952671871	0.5267152754747411	T	T	T
0.3189023406725146	-0.0003670155691109	0.6558544210532649	T	T	T
0.4414704216876286	0.3408761112405628	0.3292989410550763	T	T	T
0.4433716211415423	0.0999769158117480	0.4687097599057438	T	T	T
0.4436077006236281	0.3500375467719237	0.6071878621118810	T	T	T
0.4483120595619420	0.0901294972645494	0.7481246890496103	T	T	T
0.3185309152640310	0.0001308548258509	0.2825264309136059	T	T	T
0.3180684250427031	0.2500174537838394	0.4192762226456539	T	T	T
0.3198742514611067	0.5009539418166340	0.5513560274305892	T	T	T
0.3185519920586435	0.2452008034153013	0.6888551897222939	T	T	T
0.4436418942774210	0.1585151563725272	0.3289466104515963	T	T	T
0.4431864065933631	0.3997404895980865	0.4703622249441017	T	T	T
0.4437449718630881	0.1494471361843518	0.6052728998392355	T	T	T
0.4557063135666616	0.4059539513360828	0.7371626747014318	T	T	T
0.3283460149261850	0.2566774726160092	0.7917659922702071	T	T	T
0.0685948784952250	0.4997423535632453	0.3870256905891246	T	T	T
0.0681315189154723	0.7497117832814595	0.5259647381185518	T	T	T
0.0677929390007101	0.5011491746202887	0.6566488200755670	T	T	T
0.1942874486199718	0.8411634182954757	0.3279341549618160	T	T	T
0.1937620695264970	0.6002463783442358	0.4696992988725551	T	T	T
0.1923588022532259	0.8507504960341151	0.6034225085838665	T	T	T
0.1894650940614836	0.5948230123159902	0.7463003549071809	T	T	T
0.0661240016273626	0.4998140162384544	0.2839710958565071	T	T	T
0.0678434573029561	0.7498221348616436	0.4183141777480132	T	T	T
0.0678717794613256	0.0005512059235764	0.5483496906258375	T	T	T

0.0668245357638345	0.7516197460295789	0.6886319629881177	T	T	T
0.1908656862628508	0.6587412157450047	0.3288964403333605	T	T	T
0.1931488064967637	0.8998173320883267	0.4685540491788466	T	T	T
0.1934518773152904	0.6500491971793129	0.6055204653498386	T	T	T
0.1922500985531176	0.9100182620646411	0.7478147640924876	T	T	T
0.0668112682701950	0.7536339543415800	0.7921231419553721	T	T	T
0.3183932161288525	0.4994209842151503	0.3880234237225632	T	T	T
0.3192029490902649	0.7500267128911887	0.5249799055479001	T	T	T
0.3163453391087486	0.4987631026703865	0.6610615280290789	T	T	T
0.4445399813182154	0.8409245940722189	0.3273281366857621	T	T	T
0.4432135503546061	0.6003143492923115	0.4692700623285056	T	T	T
0.4445614460952381	0.8496813708057460	0.6068459557047667	T	T	T
0.4480499948769975	0.6048006504127786	0.7263319073895623	T	T	T
0.3158329968194219	0.5000008551144314	0.2848719171697394	T	T	T
0.3179398169884383	0.7503490582423391	0.4179732074175030	T	T	T
0.3190033514533566	0.9992563775992014	0.5481994130215969	T	T	T
0.3092126642689997	0.7588800668624421	0.6860244940382468	T	T	T
0.4410246881748981	0.6585900156277018	0.3289763127384651	T	T	T
0.4434926880479275	0.8997445079579820	0.4683936065759211	T	T	T
0.4439784514742536	0.6515375016060981	0.6074252972787827	T	T	T
0.4431365625970359	0.9070982131732443	0.7437636657794744	T	T	T
0.3243199292415339	0.7531463380352527	0.7892278118588908	T	T	T
0.5686343562152734	0.9997133262119200	0.3856407603258583	T	T	T
0.5683301812944118	0.2498934719824014	0.5263684696939820	T	T	T
0.5690025639252042	1.0003429025602963	0.6560562372814974	T	T	T
0.6914944318510818	0.3410154291809898	0.3292349882707317	T	T	T
0.6932825244351758	0.1000524053610036	0.4689128341598360	T	T	T

0.6942077181027543	0.3495796341164075	0.6051151449757565	T	T	T
0.7014302730874171	0.0900109467561978	0.7470711802236810	T	T	T
0.5687432183518952	0.0003005443829967	0.2825573757499107	T	T	T
0.5682129144711279	0.2500998021081587	0.4191330360469607	T	T	T
0.5672785495397241	0.5013966976888204	0.5497419967848152	T	T	T
0.5710740522841339	0.2437518918781474	0.6882060915102965	T	T	T
0.6936957245360454	0.1587208304962673	0.3289454659204155	T	T	T
0.6933317165152522	0.3998966897844601	0.4698209684005276	T	T	T
0.6941863071121839	0.1492753284634096	0.6050170391825267	T	T	T
0.7042576272232187	0.4079708591581488	0.7470240418214285	T	T	T
0.5759110880039194	0.2485048313860314	0.7911871672973533	T	T	T
0.8185541857977069	0.9997409948949597	0.3858351297127441	T	T	T
0.8187739135915097	0.2494278970115872	0.5265222116639401	T	T	T
0.8182299861387548	0.9985457888150213	0.6566630491121292	T	T	T
0.9415335120171927	0.3411931267115729	0.3291310438651656	T	T	T
0.9432366741666270	0.1001344855867818	0.4686805210449183	T	T	T
0.9438490638659114	0.3498355265026166	0.6060385696641801	T	T	T
0.9498323140792848	0.0908992905928381	0.7464779529189971	T	T	T
0.8186660059550499	0.0002471473315030	0.2827576523278872	T	T	T
0.8184264699641056	0.2503908981982043	0.4191269754300076	T	T	T
0.8191499369226543	0.5006843112429993	0.5485394724575138	T	T	T
0.8188813927250811	0.2493045405017777	0.6890501726551707	T	T	T
0.9436648148733973	0.1588224762849826	0.3288714491056703	T	T	T
0.9436809249374464	0.3998697706114624	0.4698930797907521	T	T	T
0.9435081783391879	0.1493847169034022	0.6065557513005738	T	T	T
0.9507813820929948	0.4081310916707711	0.7467069487608510	T	T	T
0.8269138699657501	0.2484085770618583	0.7925181369475398	T	T	T

0.5684119691957459	0.4995299192105289	0.3872692491185675	T	T	T
0.5678007819458764	0.7500083905336012	0.5259472016530430	T	T	T
0.5734474065955593	0.4986044365234159	0.6559807086370940	T	T	T
0.6944438234828262	0.8410709315882067	0.3279115742783641	T	T	T
0.6932369840331083	0.6002201459972580	0.4692025042857798	T	T	T
0.6936245435497361	0.8500865288370089	0.6062946262282419	T	T	T
0.6971761374174851	0.5938379954539548	0.7418430068526736	T	T	T
0.5651438584620684	0.4998474461094043	0.2843434578254682	T	T	T
0.5686704117314988	0.7501318951643192	0.4181568980756243	T	T	T
0.5686045216558646	0.9989847984477002	0.5483264522120532	T	T	T
0.5747613102657023	0.7572843328831280	0.6908131873639356	T	T	T
0.6910901674931792	0.6586311694462185	0.3284863348457633	T	T	T
0.6932954095856750	0.8997424928103286	0.4687837941185688	T	T	T
0.6938132861245845	0.6509288707953189	0.6067429942931245	T	T	T
0.6867546350461139	0.9063763755356505	0.7493817275599172	T	T	T
0.5588497379518617	0.7267084770475790	0.7984044965146657	T	T	T
0.8185270448160947	0.4998598741052648	0.3863126089697059	T	T	T
0.8179780882417345	0.7501364517316432	0.5261585125731751	T	T	T
0.8203523333051825	0.4998420413000355	0.6555683899365615	T	T	T
0.9445487776427143	0.8412512060048922	0.3280951282130787	T	T	T
0.9435095142668617	0.6001239536409405	0.4689083244771572	T	T	T
0.9436693024535308	0.8505008187733774	0.6054202476978668	T	T	T
0.9425277389573929	0.5921470567161212	0.7473642081982245	T	T	T
0.8155118961444628	0.4994506622755490	0.2833674455095520	T	T	T
0.8182472720508048	0.7499410288825282	0.4184113582939726	T	T	T
0.8189180482728036	0.9995969441895727	0.5485164521502958	T	T	T
0.8217517871779569	0.7529554828893215	0.6887553774088090	T	T	T

0.9407144836375338	0.6587415435142561	0.3282890516128317	T	T	T
0.9432406790167386	0.8997163778232292	0.4686003661080552	T	T	T
0.9436098308109628	0.6501156980767807	0.6057009604219368	T	T	T
0.9402664359566913	0.9084213108059529	0.7473489791681174	T	T	T
0.8135568973588702	0.7513532991627957	0.7925385272542803	T	T	T
0.3708365366948887	0.5214848483374701	0.8210576795258118	T	T	T
0.3788693549721692	0.4606095967497770	0.8412246462020575	T	T	T

Supplementary Discussion 1: Material characterization.

IFONFs almost have a constant Fe content of ~19 wt% in ICP-MS, whereas F content increase from 6.76 to 16.32 at% and the surface O content decrease significantly from 45.62 to 30.38 at% in XPS (**Supplementary Table 1**), suggesting that O atoms are partial substituted by F atoms with fluorination processing.

Note that, as $T_{fluorinated}$ prolonged from 15 to 90 min, the Fe 2$p_{3/2}$ and Fe 2$p_{1/2}$ peaks both exhibit positive shift to higher binding energy, confirming the existence of strong electronic interactions between FeF_2 and Fe_2O_3 in the hybrid (**Supplementary Fig. 5b**). While, the observed C peaks should arise from the residual organic electrolyte in the nanoporous layer (**Supplementary Fig. 6**). Therefore, the above results confirm the successful transformation of Fe-oxide into iron fluoride-oxide.

These results prove that fluorination reaction with NH_4F is a facile pathway to successfully convert iron compounds including Fe_2O_3 and FeO(OH) into FeF_2. Such a 3D nanoporous filmed and interconnected hybrid with integrated advantages of high surface area and short electron-transfer pathways may facilitate electrochemical reactions. Moreover, the fluorination method reported here is much easier and faster than other reported iron fluoride preparation methods[45, 46] because of no requirement for highly toxic materials or high-temperature growth process.

As $T_{fluorinated}$ prolonging, FeF_2 phase perfection increasing and FeF_2-F_2O_3 interfaces reducing occur simultaneously as more Fe_2O_3 nanodomains transfer into FeF_2 phase, which result in a much reduced defect state. Thereby, defect states including interphase boundary and phase junction could reach top amount with medium $T_{fluorinated}$ (*i.e.*, 45 min).

In **Supplementary Fig. 24,** Fe_2O_3 (400) surface with iron termination is combined with FeF_2 (101) surface with fluorine termination in FeF_2-F_2O_3 hybrid, and different FeF_2 (101) surfaces with fluorine termination can be got by cutting with different depths of bulk FeF_2. All of the above reasons will induce the formation of interfaces between FeF_2 and Fe_2O_3, which could significantly increase the exposure of active edge sites for heterocatalyst. In general, the heterogeneous iron fluoride-oxide nanoporous films were prepared by controlling the fluorination process. Rational $T_{fluorinated}$ is the prerequisite for the formation of defect-enriched IFONFs heterostructure. With medium $T_{fluorinated}$, the fluorination process keeps insufficient, remaining Fe-O bonds inherited from the anodized Fe-oxide, realizing partial conversion of nanoporous Fe-oxide to iron fluoride-oxide through reaction with fluorine vapor. Thus, the heterogeneous nanocomposites

possess embedded disorder phases in crystalline lattices, containing numerous scattered defects such as interphase boundaries, stacking faults, V_O and dislocations on the surfaces/interface. The defect-enriched architectures benefit for the hybrids to catalytically evolve H_2 and O_2 as they expose more interior sites derived from basal-plane/edge activity[47].

Supplementary Discussion 2: Electrochemical characterization

We hypothesize that the open and porous heterostructures of IFONFs-45 with numerous defect states and high electrical conductivity facilitate exposure of more active basal-plane/edge sites and provide more pathways for ion and mass transport. Thus, the synergistic effect from dense interior sites at the iron fluoride-oxide hybrid surface/interface endows the heterocatalyst with the significant improvement for HER kinetics.

The defect-rich feature can ensure an isotropic electron transport from Fe foil substrate to iron fluoride-oxide edges and significantly decrease the resistance for traversed layers. In addition, the 3D nanoporous filmed heterostructure promotes the release of gas bubbles, enhancing the contact between electrolyte and active sites.

Although Pt and RuO_2 are well recognized as among the most efficient catalysts to generate H_2 and O_2, respectively, they cannot perform any bifunctional activity due to the detrimental catalyst deactivation caused by either oxidation of Pt or reduction of RuO_2. The mixed phases of iron fluoride-oxide in IFONFs work for H_2 and O_2 generation catalysis during cathodic and anodic sweeps. And the highly porous morphology enhances the HER/OER activities by affording abundant active sites in an extremely low loading mass.

Given the above, the FeF_2-Fe_2O_3 direct bonding heterojunctions are successfully obtained with anodization/fluorination process. The surface contact region between the individual Fe_2O_3 and FeF_2 phases, as well as the synergetic effect of both rich active sites and good conductivity of nanoporous heterostructure lead to remarkably improved activity for electrocatalysis.

On the whole, the IFONFs delivers superior catalytic activity, which benefits from synergistic effects: unique 3D nanoporous structure with numerous defect states that ensures the sufficient exposure and better utilization of electroactive sites, and facilitates electrolyte penetration/diffusion; strong interfacial coupling and interface reconstruction between Fe_2O_3 and grafted FeF_2 by forming Fe−F bonds, which results in the charge redistribution between the Fe_2O_3 and FeF_2, and thus lowers the adsorption energy of the reactant and product; together with highly conductive support of Fe foil for efficient charge transfer.

Supplementary References

1. Qu Y, *et al.* Wafer scale phase-engineered 1T- and 2H-$MoSe_2$/Mo core–shell 3D-hierarchical nanostructures toward efficient electrocatalytic hydrogen evolution reaction. *Adv Mater* **28**, 9831-9838 (2016).

2. Zhang X, Cao C, Bieberle-Hütter A. Orientation sensitivity of oxygen evolution reaction on hematite. *J Phys Chem C* **120**, 28694-28700 (2016).

3. Liao P, Keith JA, Carter EA. Water oxidation on pure and doped hematite (0001) surfaces: Prediction of Co and Ni as effective dopants for electrocatalysis. *J Am Chem Soc* **134**,

13296-13309 (2012).

4. Liang Y, Liu Q, Asiri AM, Sun X, Luo Y. Self-supported FeP nanorod arrays: A cost-effective 3D hydrogen evolution cathode with high catalytic activity. *ACS Catal* **4**, 4065-4069 (2014).

5. Son CY, Kwak IH, Lim YR, Park J. FeP and FeP_2 nanowires for efficient electrocatalytic hydrogen evolution reaction. *Chem Commun* **52**, 2819-2822 (2016).

6. Ahn SH, Manthiram A. Direct growth of ternary Ni-Fe-P porous nanorods onto nickel foam as a highly active, robust bi-functional electrocatalyst for overall water splitting. *J Mater Chem A* **5**, 2496-2503 (2017).

7. Yang N, Tang C, Wang K, Du G, Asiri AM, Sun X. Iron-doped nickel disulfide nanoarray: A highly efficient and stable electrocatalyst for water splitting. *Nano Res* **9**, 3346-3354 (2016).

8. Wang Y, Xie C, Liu D, Huang X, Huo J, Wang S. Nanoparticle-stacked porous nickel–iron nitride nanosheet: A highly efficient bifunctional electrocatalyst for overall water splitting. *ACS Appl Mater Interfaces* **8**, 18652-18657 (2016).

9. Luo Q, Peng M, Sun X, Luo Y, Asiri AM. Efficient electrochemical water splitting catalyzed by electrodeposited NiFe nanosheets film. *Int J Hydrogen Energ* **41**, 8785-8792 (2016).

10. Yan Y, Xia BY, Ge X, Liu Z, Fisher A, Wang X. A flexible electrode based on iron phosphide nanotubes for overall water splitting. *Chem–Eur J* **21**, 18062-18067 (2015).

11. Hou Y, Lohe MR, Zhang J, Liu S, Zhuang X, Feng X. Vertically oriented cobalt selenide/NiFe layered-double-hydroxide nanosheets supported on exfoliated graphene foil: An efficient 3D electrode for overall water splitting. *Energ Environ Sci* **9**, 478-483 (2016).

12. Jia Y, *et al.* A heterostructure coupling of exfoliated Ni–Fe hydroxide nanosheet and defective graphene as a bifunctional electrocatalyst for overall water splitting. *Adv Mater* **29**, 1700017 (2017).

13. Mahmood J, *et al.* An efficient and pH-universal ruthenium-based catalyst for the hydrogen evolution reaction. *Nat Nano* **12**, 441-446 (2017).

14. Li H, *et al.* Amorphous nickel-cobalt complexes hybridized with 1T-phase molybdenum disulfide via hydrazine-induced phase transformation for water splitting. *Nat Commun* **8**, 15377 (2017).

15. Wang H, *et al.* Synthesis of single-crystal-like nanoporous carbon membranes and their application in overall water splitting. *Nat Commun* **8**, 13592 (2017).

16. Ling T, *et al.* Activating cobalt(II) oxide nanorods for efficient electrocatalysis by strain

engineering. *Nat Commun* **8**, 1509 (2017).

17. Su J, Yang Y, Xia G, Chen J, Jiang P, Chen Q. Ruthenium-cobalt nanoalloys encapsulated in nitrogen-doped graphene as active electrocatalysts for producing hydrogen in alkaline media. *Nat Commun* **8**, 14969 (2017).

18. Bau JA, Luber EJ, Buriak JM. Oxygen evolution catalyzed by nickel–iron oxide nanocrystals with a nonequilibrium phase. *ACS Appl Mater Interfaces* **7**, 19755-19763 (2015).

19. Masud J, Umapathi S, Ashokaan N, Nath M. Iron phosphide nanoparticles as an efficient electrocatalyst for the OER in alkaline solution. *J Mater Chem A* **4**, 9750-9754 (2016).

20. Liu Y, *et al.* Electrochemical tuning of olivine-type lithium transition-metal phosphates as efficient water oxidation catalysts. *Energ Environ Sci* **8**, 1719-1724 (2015).

21. Landon J, *et al.* Spectroscopic characterization of mixed Fe–Ni oxide electrocatalysts for the oxygen evolution reaction in alkaline electrolytes. *ACS Catal* **2**, 1793-1801 (2012).

22. Gong M, *et al.* An advanced Ni–Fe layered double hydroxide electrocatalyst for water oxidation. *J Am Chem Soc* **135**, 8452-8455 (2013).

23. Long X, *et al.* A strongly coupled graphene and FeNi double hydroxide hybrid as an excellent electrocatalyst for the oxygen evolution reaction. *Angew Chem Int Ed* **126**, 7714-7718 (2014).

24. Song F, Hu X. Exfoliation of layered double hydroxides for enhanced oxygen evolution catalysis. *Nat Commun* **5**, 4477 (2014).

25. Kuai L, *et al.* A reliable aerosol-spray-assisted approach to produce and optimize amorphous metal oxide catalysts for electrochemical water splitting. *Angew Chem Int Ed* **53**, 7547-7551 (2014).

26. Zhao B, *et al.* A tailored double perovskite nanofiber catalyst enables ultrafast oxygen evolution. *Nat Commun* **8**, 14586 (2017).

27. Fei H, *et al.* General synthesis and definitive structural identification of MN_4C_4 single-atom catalysts with tunable electrocatalytic activities. *Nat Catal* **1**, 63-72 (2018).

28. Li H, *et al.* Earth-abundant iron diboride (FeB_2) nanoparticles as highly active bifunctional electrocatalysts for overall water splitting. *Adv Energy Mater* **7**, 1700513 (2017).

29. Wang Z, *et al.* Porous nickel–iron selenide nanosheets as highly efficient electrocatalysts for oxygen evolution reaction. *ACS Appl Mater Interfaces* **8**, 19386-19392 (2016).

30. Yu F, *et al.* Three-dimensional nanoporous iron nitride film as an efficient electrocatalyst for

water oxidation. *ACS Catal* **7**, 2052-2057 (2017).

31. Wang Y, Liu D, Liu Z, Xie C, Huo J, Wang S. Porous cobalt-iron nitride nanowires as excellent bifunctional electrocatalysts for overall water splitting. *Chem Commun* **52**, 12614-12617 (2016).

32. Jia X, *et al.* Ni_3FeN nanoparticles derived from ultrathin NiFe-layered double hydroxide nanosheets: An efficient overall water splitting electrocatalyst. *Adv Energy Mater* **6**, 1502585 (2016).

33. Jiang H, *et al.* Iron carbide nanoparticles encapsulated in mesoporous Fe–N-doped graphene-like carbon hybrids as efficient bifunctional oxygen electrocatalysts. *ACS Appl Mater Interfaces* **7**, 21511-21520 (2015).

34. Lv C, Yang Q, Huang Q, Huang Z, Xia H, Zhang C. Phosphorus doped single wall carbon nanotubes loaded with nanoparticles of iron phosphide and iron carbide for efficient hydrogen evolution. *J Mater Chem A* **4**, 13336-13343 (2016).

35. lei y, *et al.* Fe/Fe_3C@C nanoparticles encapsulated in N-doped graphene-CNTs framework as an efficient bifunctional oxygen electrocatalyst for robust rechargeable Zn-air batteries. *J Mater Chem A* **6**, 516-526 (2018).

36. Yang Y, *et al.* Three-dimensional nanoporous Fe_2O_3/Fe_3C-graphene heterogeneous thin films for lithium-ion batteries. *ACS Nano* **8**, 3939-3946 (2014).

37. Kibsgaard J, Jaramillo TF. Molybdenum phosphosulfide: An active, acid-stable, earth-abundant catalyst for the hydrogen evolution reaction. *Angew Chem Int Ed* **53**, 14433-14437 (2014).

38. Dudarev SL, Botton GA, Savrasov SY, Humphreys CJ, Sutton AP. Electron-energy-loss spectra and the structural stability of nickel oxide: An LSDA+U study. *Phys Rev B* **57**, 1505-1509 (1998).

39. Kresse G, Joubert D. From ultrasoft pseudopotentials to the projector augmented-wave method. *Phys Rev B* **59**, 1758-1775 (1999).

40. Blöchl PE. Projector augmented-wave method. *Phys Rev B* **50**, 17953-17979 (1994).

41. Kresse G, Hafner J. *Ab initio* molecular dynamics for liquid metals. *Phys Rev B* **47**, 558-561 (1993).

42. Kresse G, Furthmüller J. Efficient iterative schemes for *ab initio* total-energy calculations using a plane-wave basis set. *Phys Rev B* **54**, 11169-11186 (1996).

43. Perdew JP, Burke K, Ernzerhof M. Generalized gradient approximation made simple. *Phys Rev Lett* **77**, 3865-3868 (1996).

44. López-Moreno S, Romero A, Mejía-López J, Muñoz A, Roshchin IV. First-principles study of electronic, vibrational, elastic, and magnetic properties of FeF_2 as a function of pressure. *Phys Rev B* **85**, 134110 (2012).

45. Zhou H, Ruther RE, Adcock J, Zhou W, Dai S, Nanda J. Controlled formation of mixed nanoscale domains of high capacity Fe_2O_3–FeF_3 conversion compounds by direct fluorination. *ACS Nano* **9**, 2530-2539 (2015).

46. Li C, *et al.* An FeF_3 $0.5H_2O$ polytype: A microporous framework compound with intersecting tunnels for Li and Na batteries. *J Am Chem Soc* **135**, 11425-11428 (2013).

47. Zhang J, *et al.* Interface engineering of MoS_2/Ni_3S_2 heterostructures for highly enhanced electrochemical overall-water-splitting activity. *Angew Chem Int Ed* **55**, 6702–6707 (2016).
